# Supplementary material for: Unplanned nursing home admission among discharged polymedicated older inpatients: a single-centre, registry-based study in Switzerland
Source: BMJ Open. 2022 Mar 4;12(3):e057444. doi: 10.1136/bmjopen-2021-057444 (PMC8900032; doi:10.1136/bmjopen-2021-057444)
Supplement: Supplementary data [file bmjopen-2021-057444supp001.pdf]

Supplementary Table 1. Descriptive statistics of the older adult inpatients' health status (N = 14,705 observations for 9,430 different subjects).

**Variables**	**Population description** (N = 14,705)
Sex	
Men n (%)	8,088 (55)
Women n (%)	6,617 (45)
Age (years)	
Mean (SD)	78.16 (7.65)
Hospital length of stay (days)	
Mean (SD)	8.63 (7.58)
Mobility	
Full ability n (%)	6,825 (63.2)
Impairment n (%)	7,880 (36.8)
Activities of Daily Living	
Full ability n (%)	12,131 (87.4)
Impairment n (%)	2,574 (12.6)
Cognitive status	
Full ability n (%)	12,622 (89.8)
Impairment n (%)	2,083 (10.2)
ICD-10 diseases (number)[1]	
Mean (SD)	4.59 (0.91)
Surgical interventions performed (CHOP) Mean (SD)	1.80 (1.77)
Most prevalent ICD-10	
Circulatory diseases n (%)	4,788 (23.4)
Infectious n (%)	559 (2.7)
Respiratory diseases n (%)	2,111 (10.3)
Traumatic injuries n (%)	2,385 (11.7)
Tumours n (%)	2,041 (10.0)

[1] Each older adult's number of ICD-10 diseases was entered into the model as a proxy for multimorbidity.
