# Supplementary material for: Unplanned nursing home admission among discharged polymedicated older inpatients: a single-centre, registry-based study in Switzerland
Source: BMJ Open. 2022 Mar 4;12(3):e057444. doi: 10.1136/bmjopen-2021-057444 (PMC8900032; doi:10.1136/bmjopen-2021-057444)
Supplement: Supplementary data [file bmjopen-2021-057444supp002.pdf]

Supplementary Table 2. Descriptive statistics of prescribed drugs at discharge based on the ATC among the polymedicated older inpatients (N = 14,705 observations for 9,430 different subjects).

**Drugs by ATC, level 2**	**Number of drugs per patient**	
	**Min–Max**	**Mean (SD)**
First level, anatomical main group		
Blood and blood-forming organ drugs (B)	0–6	1.16 (0.86)
Dermatologicals (D)	0–3	0.04 (0.22)
Genito urinary system and sex hormones (G)	0–4	0.21 (0.47)
Systemic hormonal preparations, excl. sex hormones and insulins (H)	0–4	0.20 (0.46)
Anti-infective for systemic use (J)	0–4	0.23 (0.46)
Antineoplastic and immunomodulating agents (L)	0–5	0.05 (0.23)
Musculo skeletal system drugs (M)	0–3	0.15 (0.39)
Antiparasitic products, insecticides and repellents (P)	0–2	0.02 (0.13)
Respiratory system drugs (R)	0–7	0.27 (0.72)
Sensory organ drugs (S)	0–6	0.10 (0.40)
Second level, therapeutic subgroup		
Stomatological preparations (A01)	0–1	0.01 (0.06)
Drugs for acid related disorders (A02)	0–3	0.56 (0.52)
Drugs for functional gastrointestinal disorders (A03)	0–3	0.07 (0.28)
Antiemetics and antinauseants (A04)	0–1	0.01 (0.08)
Bile and liver therapy drugs (A05)	0–1	0.01 (0.05)
Drugs for constipation (A06)	0–4	0.17 (0.42)
Anti-diarrhoeal, intestinal anti-inflammatory/anti-infective agents (A07)	0–2	0.03 (0.18)
Digestives, incl. enzymes (A09)	0–2	0.02 (0.13)
Drugs used in diabetes (A10)	0–5	0.25 (0.63)
Vitamins (A11)	0–4	0.15 (0.44)
Mineral supplements (A12)	0–3	0.30 (0.51)
Other alimentary tract and metabolism products (A16)	0–1	0.01 (0.05)
Cardiac therapy drugs (C01)	0–4	0.14 (0.41)
Antihypertensives (C02)	0–2	0.02 (0.17)
Diuretics (C03)	0–3	0.28 (0.54)
Peripheral vasodilators (C04)	0–1	0.01 (0.06)
Vaso-protectives (C05)	0–3	0.02 (0.14)
Beta-blocking agents (C07)	0–2	0.45 (0.51)
Calcium channel blockers (C08)	0–2	0.16 (0.37)
Agents acting on the Renin-Angiotensin system (C09)	0–3	0.63 (0.62)
Lipid Modifying agents (C10)	0-3	0.41 (0.52)
Anaesthetics (N01)	0–1	0.01 (0.05)
Analgesics (N02)	0–7	1.03 (0.91)
Antiepileptics (N03)	0–5	0.11 (0.36)
Anti-Parkinson drugs (N04)	0–5	0.04 (0.25)
Psycholeptics (N05)	0–7	0.57 (0.77)
Psychoanaleptics (N06)	0–3	0.21 (0.45)
Other nervous system drugs (N07)	0–3	0.03 (0.19)
Total number of drugs	5–32	9.07 (3.32)
N valid - listwise		14.70
