# Supplementary material for: Unplanned nursing home admission among discharged polymedicated older inpatients: a single-centre, registry-based study in Switzerland
Source: BMJ Open. 2022 Mar 4;12(3):e057444. doi: 10.1136/bmjopen-2021-057444 (PMC8900032; doi:10.1136/bmjopen-2021-057444)
Supplement: Supplementary data [file bmjopen-2021-057444supp003.pdf]

Supplementary Table 3. Baseline, GEE logistic regression model with unplanned nursing home admission as the dependent variable associated with sociodemographic, hospitalisation, and independent clinical and medical variables (N = 14,705 observations for 9,430 different subjects).

**Variables**	**Odds Ratio**	***p* > z**	**95%Confidence Interval**
Sex [1]	0.62	< 0.000	0.52–0.74
Age in years	1.07	< 0.000	1.05–1.08
Hospital length of stay (LOS) in days	1.02	< 0.000	1.02–1.03
Mobility [2]	3.22	< 0.000	2.67–3.87
Dependency in the activities of daily living [2]	4.62	< 0.000	3.76–5.67
Mental status [2]	3.75	< 0.000	3.06–4.59
ICD-10 principal diagnosis: circulatory problems [3]	0.78	0.030	0.63–0.98
ICD-10 principal diagnosis: infection [3]	0.38	0.002	0.20–0.70
ICD-10 principal diagnosis: respiratory problems [3]	0.91	0.511	0.70–1.19
ICD-10 principal diagnosis: injuries [3]	1.58	< 0.000	1.25–2.01
ICD-10 principal diagnosis: tumour [3]	1.33	0.071	0.98–1.80
Number of ICD-10 diagnoses	1.11	0.090	0.98–1.24
Number of surgical interventions (CHOP)	0.95	0.042	0.90–0.99
Number of prescribed drugs	1.17	0.000	1.15–1.19
Year of hospitalisation: 2015 to 2018	0.88	< 0.000	0.82–0.94

Note. 1: 0 = woman, 1 = man; 2: 0 = normal status, 1 = poor status; 3: 0 = no, 1 = yes
