# Supplementary material for: Unplanned nursing home admission among discharged polymedicated older inpatients: a single-centre, registry-based study in Switzerland
Source: BMJ Open. 2022 Mar 4;12(3):e057444. doi: 10.1136/bmjopen-2021-057444 (PMC8900032; doi:10.1136/bmjopen-2021-057444)
Supplement: Supplementary data [file bmjopen-2021-057444supp004.pdf]

Supplementary Table 4. GEE logistic regression model of the drugs prescribed to older adults at discharge with significant predictive values (odds ratios) for unplanned nursing home admission (N = 14,705 observations for 9,430 different subjects).

**Drugs**	**Odds Ratio**	***p* > z**	**95% Confidence Interval**
Antiemetics and antinauseants (A04)	2.53	0.014	1.21–5.30
Digestives, including enzymes (A09)	1.78	0.021	1.09–2.90
Psycholeptics (N05)	1.76	0.000	1.60–1.93
Antiepileptics (N03)	1.49	0.000	1.25–1.79
Anti-Parkinson drugs (N04)	1.40	0.003	1.12–1.75
Drugs for constipation (A06)	1.39	0.000	1.19–1.62
Mineral Supplements (A12)	1.28	0.001	1.10–1.49
Analgesics (N02)	1.24	0.000	1.13–1.37
Drugs for acid-related disorders (A02)	1.23	0.013	1.05–1.45
Diuretics (C03)	1.20	0.019	1.03–1.39
Psychoanaleptics (N06)	1.19	0.032	1.01–1.40
Blood and blood-forming organ drugs (B)	1.15	0.008	1.04–1.27
Drugs for the musculoskeletal system (M)	0.77	0.046	0.60–0.99
Lipid-modifying agents (C10)	0.73	0.003	0.60–0.90
