# Supplementary material for: Unplanned nursing home admission among discharged polymedicated older inpatients: a single-centre, registry-based study in Switzerland
Source: BMJ Open. 2022 Mar 4;12(3):e057444. doi: 10.1136/bmjopen-2021-057444 (PMC8900032; doi:10.1136/bmjopen-2021-057444)
Supplement: Supplementary data [file bmjopen-2021-057444supp005.pdf]

Supplementary Table 5. Factors associated with a higher and lower probability of unplanned nursing home admission among polymedicated hospitalised older adults (N = 14,705): summary of the predictive analysis.

**Risk factors for a higher probability of unplanned nursing home admission**
- Dependency in the activities of daily living (OR = 4.62, 95% CI: 3.76–5.67)
- Cognitive impairment (OR = 3.75, 95% CI: 3.06–4.59)
- Functional mobility impairment (OR = 3.22, 95% CI: 2.67–3.87)
- Antiemetics/antinauseants (OR = 2.53, 95% CI: 1.21–5.30)
- Digestives (OR = 1.78, 95% CI: 1.09–2.90)
- Psycholeptics (OR = 1.76, 95% CI: 1.60–1.93)
- Injuries (OR = 1.58, 95% CI: 1.25–2.01)
- Antiepileptics (OR = 1.49, 95% CI: 1.25–1.79)
- Anti-Parkinson's drugs (OR = 1.40, 95% CI: 1.12–1.75)
- Number of drugs prescribed (OR = 1.17, 95% CI: 1.15–1.19)
- Older age (OR = 1.07, 95% CI: 1.05–1.08)
Combined intake of:<br>- cardiac and psychoanaleptic drugs (OR = 1.87, 95% CI: 1.11–3.16)<br>- psychoanaleptic and diabetes drugs (OR = 1.75, 95% CI: 1.03–2.98)<br>- psycholeptic drugs and vitamins (OR = 1.71, 95% CI: 1.03–2.84)
Combined intake of two or more:<br>- antiemetics and antinauseants (OR = 2.65, 95% CI: 1.26–5.58)<br>- psycholeptics (OR = 1.64, 95% CI: 1.46–1.85)<br>- antiepileptics (OR = 1.55, 95% CI: 1.23–1.96)<br>- anti-Parkinson's drugs (OR = 1.44, 95% CI: 1.13–1.83)
**Protective factors for a lower probability of unplanned nursing home admission**
- Surgical interventions (OR = 0.95, 95% CI: 0.90–0.99)
- Circulatory diseases (OR = 0.78, 95% CI: 0.63–0.98)
- Lipid metabolism modifying agents (OR = 0.73, 95% CI: 0.60–0.90)
- Male sex (OR = 0.62; 95% CI: 0.52–0.73)
- Combined intake of beta-blocking agents and antiepileptics (OR = 0.39, 95% CI: 0.23–0.67)
- Infectious diseases (OR = 0.38, 95% CI: 0.20–0.70)
