# Supplementary material for: Unplanned nursing home admission among discharged polymedicated older inpatients: a single-centre, registry-based study in Switzerland
Source: BMJ Open. 2022 Mar 4;12(3):e057444. doi: 10.1136/bmjopen-2021-057444 (PMC8900032; doi:10.1136/bmjopen-2021-057444)
Supplement: Author's manuscript [file bmjopen-2021-057444.draft_revisions.pdf]

BMJ Open

BMJ Open is committed to open peer review. As part of this commitment we make the peer review history of every article we publish publicly available.

When an article is published we post the peer reviewers’ comments and the authors’ responses online. We also post the versions of the paper that were used during peer review. These are the versions that the peer review comments apply to.

The versions of the paper that follow are the versions that were submitted during the peer review process. They are not the versions of record or the final published versions. They should not be cited or distributed as the published version of this manuscript.

BMJ Open is an open access journal and the full, final, typeset and author-corrected version of record of the manuscript is available on our site with no access controls, subscription charges or pay-per-view fees (http://bmjopen.bmj.com).

If you have any questions on BMJ Open’s open peer review process please email info.bmjopen@bmj.com

BMJ Open

Unplanned institutionalization among discharged polymedicated older inpatients: a registry-based study

Journal:	*BMJ Open*
Manuscript ID	bmjopen-2021-057444
Article Type:	Original research
Date Submitted by the Author:	15-Sep-2021
Complete List of Authors:	Pereira, Filipa; University of Porto, Institute of Biomedical Sciences Abel Salazar; HES-SO Valais Wallis, School of Health Sciences<br>Verloo, Henk; HES-SO Valais Wallis, School of Health Sciences<br>Von-Gunten, Armin; University of Lausanne Hospital Centre<br>Meyer-Massetti, Carla; University of Bern, Institute for Primary Health Care (BIHAM)<br>del Río Carral, María; University of Lausanne, Institute of Psychology, Research Center for the Psychology of Health, Aging and Sports Examination<br>Martins, Maria Manuela; Escola Superior de Enfermagem do Porto, Formação&gestão<br>Wernli, Boris; University of Lausanne, FORS, Swiss Centre of Expertise in the Social Sciences
Keywords:	GERIATRIC MEDICINE, CLINICAL PHARMACOLOGY, EPIDEMIOLOGY

SCHOLARONE™
Manuscripts

I, the Submitting Author has the right to grant and does grant on behalf of all authors of the Work (as defined in the below author licence), an exclusive licence and/or a non-exclusive licence for contributions from authors who are: i) UK Crown employees; ii) where BMJ has agreed a CC-BY licence shall apply, and/or iii) in accordance with the terms applicable for US Federal Government officers or employees acting as part of their official duties; on a worldwide, perpetual, irrevocable, royalty-free basis to BMJ Publishing Group Ltd ("BMJ") its licensees and where the relevant Journal is co-owned by BMJ to the co-owners of the Journal, to publish the Work in this journal and any other BMJ products and to exploit all rights, as set out in our licence.

The Submitting Author accepts and understands that any supply made under these terms is made by BMJ to the Submitting Author unless you are acting as an employee on behalf of your employer or a postgraduate student of an affiliated institution which is paying any applicable article publishing charge ("APC") for Open Access articles. Where the Submitting Author wishes to make the Work available on an Open Access basis (and intends to pay the relevant APC), the terms of reuse of such Open Access shall be governed by a Creative Commons licence – details of these licences and which Creative Commons licence will apply to this Work are set out in our licence referred to above.

Other than as permitted in any relevant BMJ Author's Self Archiving Policies, I confirm this Work has not been accepted for publication elsewhere, is not being considered for publication elsewhere and does not duplicate material already published. I confirm all authors consent to publication of this Work and authorise the granting of this licence.

For peer review only

Unplanned institutionalization among discharged polymedicated older inpatients: a registry-based study

Filipa Pereira[1,2], Henk Verloo[1,3], Armin von Gunten[c], María del Río Carral[4], Carla Meyer-Massetti[5], Maria Manuela Martins[6], Boris Wernli[7]

[1] School of Health Sciences, HES-SO Valais/ Wallis, University of Applied Sciences and Arts Western Switzerland, Sion, Switzerland

[2] Institute of Biomedical Sciences Abel Salazar, University of Porto, Porto, Portugal

[3] Service of Old Age Psychiatry, Lausanne University Hospital, Lausanne, Switzerland

[4] Institute of Psychology, Research Center for the Psychology of Health, Aging and Sports Examination, University of Lausanne, Lausanne, Switzerland

[5] Institute for Primary Health Care (BIHAM), University of Bern, Bern, Switzerland

[6] Higher School of Nursing of Porto, Porto, Portugal

[7] FORS, Swiss Centre of Expertise in the Social Sciences, University of Lausanne, Switzerland

Corresponding Author:

Filipa Pereira
School of Health Sciences, HES-SO Valais / Wallis
University of Applied Sciences and Arts Western Switzerland
5 Chemin de l'Agasse, CH-1950 Sion, Switzerland
Institute of Biomedical Sciences Abel Salazar, University of Porto
228 Rua de Jorge Viterbo Ferreira, 4050-313 Porto, Portugal
Phone: +41 78 666 17 00
Email: filipa.pereira@hevs.ch
ORCID: https://orcid.org/0000-0001-9207-4856

For peer review only

Abstract

Objective: To investigate patient characteristics and the available health and drug data associated with unplanned institutionalization following an acute hospital admission or readmission.

Design: A population-based hospital registry study.

Setting: A public hospital in southern Switzerland (Valais Hospital).

Participants: We explored a population-based longitudinal dataset of 14,705 hospital admissions from 2015–2018.

Outcome measures: Sociodemographic, health, and drug data and their interactions predicting the risk of unplanned institutionalization.

Results: The mean prevalence of unplanned institutionalization after hospital discharge was 6.1%. Our predictive analysis revealed that the oldest adults (OR=1.07 for each additional year of age; 95% CI 1.05 to 1.08) presenting with impaired functional mobility (OR=3.22; 95% CI 2.67 to 3.87), dependency in the activities of daily living (OR=4.62; 95% CI 3.76 to 5.67), cognitive impairment (OR=3.75; 95% CI 3.06 to 4.59), and traumatic injuries had a higher probability of unplanned institutionalization (OR=1.58; 95% CI 1.25 to 2.01). The number of ICD-10 diagnoses had no significant impact on institutionalization, contrarily to the number of prescribed drugs (OR=1.17; 95% CI 1.15 to 1.19). Antiemetics/antinauseants (OR=2.53; 95% CI 1.21 to 5.30), digestives (OR=1.78; 95% CI 1.09 to 2.90), psycholeptics (OR=1.76; 95% CI 1.60 to 1.93), antiepileptics (OR=1.49; 95% CI 1.25 to 1.79), and anti-Parkinson's drugs (OR=1.40; 95% CI 1.12 to 1.75) were strongly linked to unplanned institutionalization.

Conclusions: Numerous determinants of unplanned institutionalization were identified. To prevent the adverse health outcomes that precipitate acute hospitalizations and unplanned institutionalizations, ambulatory care providers should consider these determinants in their care planning for older adults before they reach a state requiring hospitalization.

Keywords: population-based sample; functional decline; hospital discharge; risk factors; nursing home

For peer review only - http://bmjopen.bmj.com/site/about/guidelines.xhtml

Strengths and limitations of this study:

- A hospital registry of 14,705 hospital admissions, involving 9,430 different polymedicated older adults admitted from their homes, was analyzed to determine the risk of unplanned institutionalization.
- Bivariate analyses were conducted on independent variables, and generalized estimating equations were computed to predict how sets of predictors influenced the probability of unplanned institutionalization.
- Causality analysis was not feasible based on the nature of the routinely collected data.
- Although the study considered statistical associations between drugs and unplanned institutionalization, it did not use clinically diagnosed drug–drug interactions.
- Our data were unable to identify hospitalizations that might have been triggered by limited home-care options or those that became necessary while older adults awaited a place in a long-term care facility.

Introduction

The hospitalization of home-dwelling older adults, for any reason and even for a short admission, can lead to substantial functional decline [1, 2]. Both their health disorder itself and the hospital environment can foster such functional decline, increase the risk of future illness, and irreversibly diminish their quality of life [1, 2]. Most hospitalized older adult inpatients wish to return home and continue their everyday life as before. However, these different factors may hinder this wish at discharge [3, 4]. Unmet patient needs related to functional decline and safety after returning home can lead to a higher risk of hospital and emergency department readmissions and thus to subsequent unplanned institutionalization [5]. After hospitalization, an unplanned institutionalization can be a devastating and overwhelming experience for older adults and their relatives, and it increases overall health-care system costs [6].

Whether planned or unplanned, institutionalization commonly follows two paths: (i) within the community, directly from home, or (ii) from hospital, directly transitioning from hospital discharge [2]. In the community, transitions to nursing homes are generally the result of thoughtful decisions made by home-dwelling older adults, their families, and health- and social-care providers based on their knowledge of the evolution of the person’s long-term health and functional state or on an acute decline and corresponding increase in care needs that cannot be met at home. Recent findings have suggested that the predictors of institutionalization are mainly based on underlying cognitive

For peer review only - http://bmjopen.bmj.com/site/about/guidelines.xhtml

and functional impairments combined with a lack of support and assistance in daily living at home [7].

The causes of unplanned institutionalization directly after acute hospital discharge are heterogeneous. There are several reasons why older adults may require long-term care—that cannot be provided in a community setting—following acute hospital admission, e.g., a new medical problem or the worsening of existing chronic disease(s) entailing dependency and requiring complex forms of care. Furthermore, there may be a breakdown of family circumstances and/or lack of social support. Some studies have noted that hospitalized older inpatients had already suffered a significant deterioration in their individual scores for mobility, transfer, toileting, feeding, grooming, and cognitive status by their second day in hospital [1, 4, 8].

Bellelli *et al*. showed that advanced age (OR=4.8; 95% CI 2.6 to 8.9, $p < 0.001$), cognitive impairment (OR=2.3; 95% CI 1.4 to 3.9, $p < 0.001$), and poor functional status (OR=10.2; 95% CI 4.7 to 22.5, $p < 001$) at discharge from a rehabilitation unit were the main predictors of subsequent institutionalization [9]. The integrative review by Fogg *et al*. found a similar result for cognitive impairment (OR=2.14; 95% CI 1.24 to 3.70, $p < 0.001$) [10]. A randomized controlled trial by Landefeld *et al*. found that older inpatients in an acute care medical unit with a decline in their ability to perform one or more of the basic activities of daily living (ADL) were more often discharged to a nursing home than those with less functional decline (22% and 14 %, respectively; $p < 0.01$) [11]. Ferrucci *et al*. identified stroke, cancer, congestive heart failure, pneumonia, coronary heart disease, and hip fractures as the leading medical precipitators of functional decline and institutionalization [12]. Older adult inpatients are frequently subject to iatrogenic events during hospitalization, including adverse drug reactions, nosocomial infections, and the consequences of falls, fractures, and using chemical or physical restraints [13]. Such events can lengthen hospitalization, produce cognitive changes, and lessen the ability to perform the ADL, all potentially leading to unplanned institutionalization [13].

Kasper suggested that repeated cycles of atrophy and recovery may lead an older adult to lose their ability to restore skeletal muscle mass, thus becoming permanently disabled and unable to remain at home [14]. Indeed, functional decline may cause significant sarcopenia—which occurs more rapidly in older patients—and can lead to falls, frailty, and unplanned institutionalization [1]. Using Fried's criteria, Rosenberg *et al*. found that frail older adults were at greater risk of adverse drug events generated by prescriptions of potentially inappropriate medication (PIM), falls, and institutionalization [15]. In a prospective cohort study of 210 frail older adult inpatients (mean age 89.4; SD = 4.6; 69.5% female), Chong *et al*. found a high risk of institutionalization (OR=3.69; 95% CI

For peer review only - http://bmjopen.bmj.com/site/about/guidelines.xhtml

2.31 to 5.88; $p < 0.001$), adjusted for age, sex, and severity of illness [16]. In their prospective cohort study (N = 140), Troester *et al*. confirmed the significant risk of institutionalization among frail older inpatients (mean age 84.1; SD = 8.6) after a mean hospital stay of about 30 days (SD = 16.5) [17]. Other investigators have found that patients with the greatest loss of independence in the ADL during hospitalization were the most likely to be admitted to a nursing home [4, 15].

In addition to functional decline, cognitive impairment is among the strongest factors predicting institutionalization [1]. Indeed, hospitalization also causes an increased risk of the onset of acute cognitive decline in the form of delirium, with a prevalence of up to 60% on some surgical wards [18], often leading to unplanned institutionalization [19]. Dementia, Parkinson's disease and its associated risk of falls, and behavioral changes are common reasons for deciding to transfer inpatients from hospital to long-term care [20, 21].

Polypharmacy has been associated with adverse health outcomes among home-dwelling older adults [22]. Some prospective studies with small samples have established relationships between drug treatments during acute hospitalization and unplanned institutionalization [23]. Cardiovascular drugs (particularly vasodilators, diuretics, and anticoagulants), drugs against diabetes, steroids, non-steroidal anti-inflammatory drugs, opiates, antibiotics, anticholinergics, and benzodiazepines have all been associated with unplanned institutionalization [23].

To the best of our knowledge, and despite more frequent post-discharge institutionalization in Switzerland than in other countries, there is scarce research exploring how unplanned admissions to nursing homes are related to prior hospitalization [24]. The present study aimed to investigate the associations between polymedicated older inpatients' sociodemographic and clinical characteristics, drug data, and their interactions, and their unplanned institutionalization following an acute care hospital stay.

Materials and Methods

Study design

The present population-based hospital registry study was conducted with close regard to the REporting of studies Conducted using Observational Routinely collected health Data (RECORD) statement.

Population and data collection

Our four-year, longitudinal, population-based hospital registry of electronic health records included polymedicated (five and more drugs prescribed) home-dwelling older adults admitted and

For peer review only - http://bmjopen.bmj.com/site/about/guidelines.xhtml

readmitted to the Valais hospital, a multisite public teaching hospital in southern Switzerland with a mean annual number of hospitalizations of approximately 39,000. This registry continues to be analyzed as part of a larger project [25]. Ethical approval was obtained from the Human Research Ethics Committee of the Canton of Vaud (2018-02196), and this permitted the partnering hospital's data warehouse to provide the appropriate dataset. Our study defined 'unplanned institutionalization' as the impossibility for a formerly home-dwelling older inpatient to return there after hospital discharge, and this included any new institutionalization in a long-term residential care facility following an acute care admission [2]. All the patients included in the study followed a home to hospital to long-term residential care facility pathway. Long-term residential care facilities do not expect their residents to return to independent living in the community. The extracted patient data contained sociodemographic characteristics, medical and surgical diagnoses, routinely assessed clinical data (such as gait, falls risk, hearing, or pain), and the drugs prescribed. The medical and surgical diagnoses encoded diagnostic data using the WHO International Classification of Diseases, tenth version (ICD-10), and the Swiss Classification of Surgical Interventions (CHOP) [26]. The hospital dataset showed that discharged patients had been prescribed 2,370 different medicines. The Anatomical Therapeutic Chemical (ATC) classification system's 14 top-level codes was used to structure that dataset of prescribed medicines [27]. The extracted data, from multiple dataset sources, were transformed and synthesized using best practices [28]. Our dataset was composed of 14,705 hospital admissions from home settings between 2015 and 2018. Data were without missing values, and there were similar numbers of annual hospital admissions: 3,777, 3,534, 3,724, and 3,670, respectively.

For peer review only

Patient and public involvement

Patients were not involved in the development of the research questions, study design, outcome measures, or the conduct of the study.

Dataset customizing for predictive analysis

Synthetizing the extracted data

The dataset was recoded and customized to identify the number of older inpatients admitted straight from their home and then discharged to a nursing home, as presented in a previous paper [29]. Each subject's unique identifier was used to distinguish different observations from 2015 to 2018 and to account for hospital readmissions. Cases involved 9,430 different older adults, with an average of 1.56 hospital stays per person. Sociodemographic and clinical data were considered independent variables and used to compute the predictive models [29]. Unplanned

For peer review only - http://bmjopen.bmj.com/site/about/guidelines.xhtml

institutionalization after discharge from our participating hospital between 2015 and 2018 was identified by the difference between the abode of origin (home) and the abode of destination at discharge (a long-term residential care facility), and this was recoded as the dependent variable of interest.

Sociodemographic and Hospital Variables

The analysis included two sociodemographic control variables: age and sex. Fifty-five percent of the population sample were women, and the total sample's mean age was 78.16 years old (SD = 7.65). Age was considered a continuous variable; its progressive impact was conclusive in preliminary investigations and previous studies [30]. Mean hospital length of stay was 8.63 days (SD = 7.58) [29].

Health Variables

Numerous variables were used to describe older adults' health status at the end of their hospital stay. The modeling analysis included three of the six hierarchical clusters preliminarily computed as confounding variables: mobility, dependency in the ADL, and cognitive status [29]. Cognitive status was measured at an ordinal level using five categorical variables (perception–alertness, orientation, attention, decision making process, and ability to learn).

At discharge, 28% of subjects presented with impaired mobility, and 6% were impaired in their ADL and cognitive status. The mean number of ICD-10 diseases per subject was 4.59 (SD = 0.91), and each older adult's number of ICD-10 diseases was entered into the model as a proxy for multimorbidity. The mean number of surgical interventions performed (based on the Swiss surgery coding system CHOP) [26] was 1.80 per hospitalization (SD = 1.77). The most prevalent medical diagnoses among older inpatients were circulatory (24% of the population sample), infectious (3%), and respiratory diseases (10%), as well as traumatic injuries (8%) and tumors (11%). Finally, the year of hospitalization was introduced as a control variable, based on the fact that hospital admissions occurring earlier in the four-year study were associated with a higher probability of unplanned institutionalization [29].

Drugs

The WHO ATC Classification System [27] was used to select frequently prescribed drugs at discharge as independent variables for the predictive model. The selection of drug class interactions was based on a literature review and expert opinions [31]. A cut-off point of at least 30 subjects per drug category prescribed was necessary to have a critical mass of data for computing robust statistics. The number of drugs prescribed at hospital discharge was considered continuous, with an average of

For peer review only - http://bmjopen.bmj.com/site/about/guidelines.xhtml

9.07 (SD = 3.32). Supplementary Table 1 presents the descriptive statistics of selected drugs based on ATC.

Data analysis strategy

Data were extracted into a Microsoft Excel spreadsheet (Microsoft, Redmond, Washington, United States) and subsequently imported into SPSS software, version 26.0 (IBM Corp, Armonk, New York, United States). Associations with unplanned institutionalization were examined based on previous studies: patient age and sex, hospital length of stay, the principal and secondary ICD-10 diagnoses, surgical interventions (CHOP), and prescribed drugs. No causality analyses were possible because data analysis was retrospective and based on routine data: there was no way of knowing medication regimens or functional status before hospitalization and how these might be associated with unplanned institutionalization. A bivariate analysis was conducted using cross-tabulations relating to the independent variables of unplanned institutionalizations from 2015 to 2018. In a second stage, a series of generalized estimating equations (GEE or population-averaged logistic regression models) were computed to predict how sets of predictors influenced the probability of unplanned institutionalization. The model estimated each predictor's impact, other things being equal, by estimating its net impact controlling for confounding factors (adjusted odds ratios). This GEE model is generally considered very robust and efficient at dealing with panel or correlated data because it makes few explicit assumptions and is less vulnerable to misspecification [32]. A GEE model predicts for the entire population and not a specific individual. Since the data are based on a whole population, not a sample, the odds ratios' confidence intervals and statistical tests were used to indicate the robustness of relationships since they normally only make sense for statistical inference.

Results

Unplanned institutionalization, sociodemographic characteristics, and the prevalence of clinical and medical conditions

We found a prevalence of older adults discharged to unplanned institutionalization of 6.1% over the whole time period, with a slight decrease in prevalence going forward (7.3% in 2015 to 5.9% in 2018). Bivariate associations showed that men had a lower prevalence of unplanned institutionalization than women (4.0% vs. 8.8%), as did 65–69-year-old subjects (2.2%) compared with those 70–79 years old, 80–89 years old, and especially the oldest group, aged 90 or more (3.2%, 8.3%, and 19.7%, respectively).

For peer review only - http://bmjopen.bmj.com/site/about/guidelines.xhtml

Subjects with unimpaired mobility (2.0%), less dependency in their activities of daily living (3.4%), and a good cognitive status (3.8%) showed much lower probabilities of unplanned institutionalization than those with poor mobility, greater dependency, and a poor cognitive status (16.7%, 44.8%, and 41.3%, respectively). However, a higher prevalence of unplanned institutionalization was observed among older adults without a circulatory disease (6.7% vs. 4.3%), unaffected by an infection (6.2% vs. 2.7%), or without a tumor (6.4% vs. 4.3%). Those affected by traumatic injuries showed a significantly higher prevalence (14.9% vs. 5.3%). Being jointly affected by several diseases increased the prevalence of unplanned institutionalization, from 1.8% for older adults with a single disease (ICD-10) to 6.8% for those with five diseases. Furthermore, the number of surgical interventions was negatively associated with the prevalence of unplanned institutionalization. Patients who had not undergone surgery showed a higher probability of unplanned institutionalization (7.8%) than those who had undergone several interventions (3.5% for four interventions, 4.2% for five interventions) (Table 1). The number of drugs prescribed at hospital discharge showed a positive linear relationship with unplanned institutionalization (g= .368) (Fig. 1).

[Insert Table 1]

[Insert Figure 1]

Unplanned institutionalization and drugs

Bivariate associations showed that drugs were also related to unplanned institutionalization (Table 2). In general, older adults whose discharge to an institution was unplanned had more prescribed drugs than those returning home (10.9 drugs vs. 8.9). Psycholeptics (antipsychotics, anxiolytics, hypnotics, and sedatives) and psychoanaleptic drugs (antidepressants, psychostimulants, nootropics, and anti-dementia drugs), antiemetics and antinauseants, anti-Parkinson's disease drugs, and drugs treating constipation and the sensory organs were significantly associated with unplanned institutionalization. On the contrary, patients taking lipid-modifying agents were less prone to unplanned institutionalization.

[Insert Table 2]

Multivariate baseline model

A baseline, GEE logistic regression model, including sociodemographic information, clinical data, and diseases, was computed to predict unplanned institutionalization among discharged polymedicated older adult patients (Fig. 2); prescribed drugs at hospital discharge were not included. If the 95% confidence interval (CI) does not overlap the null value (e.g., OR = 1), then the higher the odds ratio, the more the variable contributes to unplanned institutionalization. Men had a lower probability of

For peer review only - http://bmjopen.bmj.com/site/about/guidelines.xhtml

unplanned institutionalization than women (OR=0.62; 95% CI 0.52 to 0.73). Patients' probability of unplanned institutionalization increased with age (OR=1.07 for each additional year of age; 95% CI 1.05 to 1.08). Impaired mobility, dependency in the ADL, and cognitive impairment revealed their substantial impacts on unplanned institutionalization (OR=3.22; 95% CI 2.67 to 3.87; OR=4.62; 95% CI 3.76 to 5.67; and OR=3.75; 95% CI 3.06 to 4.59, respectively). Circulatory and infectious diseases were related to lower probabilities of unplanned institutionalization (OR=0.78; 95% CI 0.63 to 0.98, and OR=0.38; 95% CI 0.20 to 0.70, respectively), whereas traumatic injuries were related to higher probabilities (OR=1.58; 95% CI 1.25 to 2.01). The number of ICD-10 diagnoses alone had no significant impact on the odds of unplanned institutionalization (OR=1.11; 95% CI 0.98 to 1.24), in contrast to the number of surgical interventions undergone (CHOP), which was a protective factor against unplanned hospitalization (OR=0.95; 95% CI 0.90 to 0.99). The year of hospital stay also had a significant impact, with more recent stays having lower probabilities of unplanned institutionalization (OR=0.88; 95% CI 0.82 to 0.94, per ensuing year).

[Insert Figure 2]

Prediction of unplanned institutionalization and drug prescription

A higher number of prescribed drugs was associated with a higher probability of unplanned institutionalization (OR=1.17; 95% CI 1.15 to 1.19). Figure 3 presents the baseline GEE logistic regression model from Figure 2, completed with those drugs prescribed to older adults at discharge that had a significant statistical association ($p < 0.05$) with unplanned institutionalization. Drugs without a significant statistical association are not presented in Figure 3 for simplification purposes. Antiemetics and antinauseants (OR=2.53; 95% CI 1.21 to 5.30 for each additional unit), digestives (OR=1.78; 95% CI 1.09 to 2.90), psycholeptics (OR=1.76; 95% CI 1.60 to 1.93), antiepileptics (OR=1.49; 95% CI 1.25 to 1.79), and anti-Parkinson's disease drugs (OR=1.40; 95% CI 1.12 to 1.75) were strongly linked to unplanned institutionalization after controlling for other parameters. On the contrary, taking lipid metabolism modifying agents was associated with lower probabilities of unplanned institutionalization (OR=0.73; 95% CI 0.60 to 0.90, for each extra drug from this class prescribed).

[Insert Figure 3]

Combined drug intake and probabilities of unplanned institutionalization

To reduce collinearity and simplify the results, the combined intake of different ATC drug classes was recoded as a dichotomized variable for each drug pairing and added to the previous model [27]. Only the drugs and drug combinations prescribed to older adults at discharge that had significant

associations ($p < 0.05$) with unplanned institutionalization are presented. The combined intake of cardiac therapy and psychoanaleptic drugs was significantly associated with unplanned institutionalization (OR=1.87; 95% CI 1.11 to 3.16), as were psychoanaleptics and diabetes drugs combined (OR=1.75; 95% CI 1.03 to 2.98), and psycholeptic drugs and vitamins combined (OR=1.71; 95% CI 1.03 to 2.84). On the contrary, the combined intake of beta-blocking agents and antiepileptics strongly diminished the odds of unplanned institutionalization (OR=0.39; 95% CI 0.23 to 0.67).

We also investigated the risk of unplanned institutionalization for combined drug intake within the same drug class. The combined intake of two or more antiemetic and antinauseants (OR=2.65; 95% CI 1.26 to 5.58), psycholeptics (OR=1.64; 95% CI 1.46 to 1.85), antiepileptics (OR=1.55; 95% CI 1.23 to 1.96), or anti-Parkinson's disease drugs (OR=1.44; 95% CI 1.13 to 1.83) were strongly associated with a higher probability of unplanned institutionalization.

Table 3 summarizes the main findings from our predictive analysis.

[Insert Table 3]

Discussion

This population-based hospital registry study used longitudinal data to examine the unplanned institutionalization of hospitalized polymedicated older inpatients, revealing a 6.1% prevalence rate over the four-year dataset, in agreement with previous work by Luppa *et al*. (men: 5.4%; women: 6.0%) and Goodwin *et al*. (5.5%) [7, 33]. The slight decrease in prevalence over the four years of the study may be explained by improvements in the regional home-care services' contribution to maintaining older adults at home, but also to planned institutionalizations without the requirement for intermediate hospitalization [34]. Furthermore, the number of places in the region's long-term care facilities increased in that period [35], allowing people for whom care at home became impossible to be institutionalized more promptly.

Our predictive analysis revealed that the group of the oldest adults, presenting functional mobility impairments, dependency in the ADL, and cognitive impairment were also at a high risk of unplanned institutionalization, which is consistent with previous retrospective and prospective studies [8, 36]. Very old inpatients (≥ 90 years old) had an almost tenfold higher risk of unplanned institutionalization than those aged 65–69. This was expected and matched with previous research [37], bearing in mind that the very oldest group presented with a high prevalence of multimorbidity and advanced functional and cognitive impairments. Unexpectedly, regardless of age, our results showed that older women had a higher prevalence and probability of unplanned institutionalization

For peer review only - http://bmjopen.bmj.com/site/about/guidelines.xhtml

than men [38]. Yet our data could not entirely explain this result. Previous publications have indicated that social and life-expectancy factors may play roles in the different rates of institutionalization between older adult men and women[38].

Our findings highlighted that functional and cognitive impairments were strong risk factors for unplanned institutionalization, which is in line with the studies by Luppa *et al*. and Goodwin *et al*. [33, 38]. Likewise, our results emphasized a high risk of unplanned institutionalization among non-surgically treated and trauma patients This could be explained by the relationship between orthopedic guidelines on traumatic injuries among older adults that suggest avoiding surgery, for several medical reasons (number and severity of multimorbidities), and which may lead to increased functional impairment and unplanned institutionalization, as suggested by Gardner *et al*. and Cutugno [39, 40].

As might be expected, older adults who underwent an unplanned institutionalization had more prescribed drugs than those returning home. Our results were in line with the retrospective study by Lucchetti *et al*., which demonstrated a relationship between the prescription of cardiovascular, gastrointestinal, and metabolic drugs and unplanned institutionalization [41].

Our findings indicated that patients prescribed more than one drug from the same class of drugs—from the classes of antiemetics and antinauseants, psycholeptics, antiepileptics, or anti-Parkinson's disease drugs—had a higher risk of discharge to an institution. Although this phenomenon is still under-investigated, our findings are not in line with the few existing studies in this area, which have presented no significant relationships between drug interactions and unplanned institutionalization [42]. However, in hospital settings, a recent systematic review reported drug–drug interactions among 80% or more of older inpatients [43]. Since polymedicated older inpatients should be considered as a population at a high risk of adverse outcomes, further studies should investigate how drug–drug interactions might predict the risks of institutionalization.

Our findings undeniably mirrored existing evidence that chronic conditions and debilitating comorbidities are significant determinants of unplanned institutionalization [3, 7]. However, they also raised questions regarding hospitalization's effects on the individual aging process, which likely interact to produce a cascade of factors towards functional decline and dependency [1]. The adverse effects of hospitalization begin immediately and progress rapidly [1]. Harrison *et al*. and Haaksma *et al*. described ways in which acute and exacerbated acute and chronic disorders, reinforced by existing undiagnosed geriatric syndromes (frailty, delirium, pressure sores, functional incontinence), contributed to hospitalized older patients being unable to return home and needing to be discharged to a long-term residential care facility [2, 21]. Previous studies suggested that silent

For peer review only - http://bmjopen.bmj.com/site/about/guidelines.xhtml

geriatric syndromes such as frailty and functional decline, together with polypharmacy, are not only clinically characteristic of older adults but also potential predictors of being at risk of a further loss of independence and subsequent institutionalization. Montes *et al*. pointed out the dramatic rise in numbers of frail hospitalized older adults. This increase generates concerns about whether long-term residential care facilities—already suffering from long admission waiting lists of home-dwelling older adults—will be able to cope with older adults' complex care needs [44].

Although some of the predisposing predictors identified cannot be treated (i.e., sex, age), they may still contribute to an older adult's risk of being discharged to a long-term residential care facility and subsequently exacerbate their situation there. Given that hospitalization introduces stressors that may increase the chances of unplanned institutionalization [45, 46], using patients' electronic hospital data could help identify the high-risk older adults who would benefit from specific preventive interventions. Being able to rapidly identify inpatients at a high risk of unplanned institutionalization may help professional caregivers to provide them with the appropriate community-health resources, such as community-based rehabilitation programs. This would help older people to remain in their community for longer.

Study strengths and limitations

Although our population-based study's findings could be generalized to other regions of Switzerland, any interpretations should be made with caution. Nevertheless, our findings could provide information to help better define which integrated health-care approaches could be implemented to attenuate the risk factors associated with unplanned institutionalization following an acute hospital admission or readmission. The numerous predictors revealed in our study enabled us to conceptualize an overview of hospitalized older adults' health conditions before their unplanned institutionalization. As health-care moves towards ever-more personalized medicine, this result could help to create more refined, tailored, future interventions via 'risk profiles' defined using each older adult's personal predictors.

Our study had some limitations. The absence of data on patients' functional status before hospital admission meant that we could not assess changes to that status during hospitalization, such as the influence of the development or deterioration of functional and cognitive impairment. We did not compute analysis on specific disorders such as neurodegenerative diseases like dementia and Parkinson's disease because this was beyond the scope of our study protocol. However, further analyses could confirm earlier studies showing that these diseases significantly affect a person's risk of institutionalization after hospitalization, with almost 90% of patients with dementia being admitted into a long-term residential care facility before dying [20, 21]. Although the study

For peer review only - http://bmjopen.bmj.com/site/about/guidelines.xhtml

considered statistical associations between drugs and unplanned institutionalization, it did not use clinically diagnosed drug–drug interactions. Lastly, our data were unable to identify hospitalizations that might have been triggered by limited care options at home or hospitalizations that were necessary while awaiting a place in a long-term care facility. These cases of planned institutionalization could not be distinguished from the unplanned institutionalization considered in the study. In addition, some patients may not have been transferred directly from hospital to long-term care facilities and may have had to stay in an intermediate structure while awaiting a place. These patients were not included in the study due to the unavailability of this information in the database.

Conclusion

The sociodemographic characteristics of hospitalized older inpatients, together with their clinical and medical conditions and their prescribed drugs, can provide us with a significant set of risk determinants of individuals' potential for unplanned institutionalization, sustaining our stated hypotheses. Identifying the risk factors for unplanned institutionalization could be of great assistance in developing predictive tools and tailored intervention programs aimed at reducing the number of older adults placed in long-term residential care facilities. Our results showed that the patient-related risk factors leading to institutionalization were based on declines in physical and cognitive function. Treatment with single drugs and combinations of drugs were also associated with unplanned institutionalization, indicating that multiple chronic health conditions are important determinants of a non-return home. Our findings may help to identify those older inpatients at the greatest risk of unplanned institutionalization, enabling their care to be optimized by counterbalancing those risk factors. Further research is required across large samples of older inpatients to investigate whether tailored interventions at early stages in chronic diseases could delay physical and cognitive dysfunction and reduce unplanned institutionalizations among this growing segment of the population.

Acknowledgement

The authors thank the partner hospital, including the hospital's data warehouse, for its valuable contributions.

Ethics approval and patient consent

Ethical approval was obtained from the Human Research Ethics Committee of the Canton of Vaud (CER-VD, 2018-02196), thus permitting our partner hospital's data warehouse to provide the appropriate dataset. Given the retrospective data source, obtaining consent from the patients

For peer review only - http://bmjopen.bmj.com/site/about/guidelines.xhtml

concerned was impossible or posed disproportionate difficulties. The present study respects the legal requirements for research projects involving data re-use without consent, as set out in Art. 34 from the Swiss Human Research Act (HTA).

Conflict of Interest Statement

The authors have no conflicts of interest to declare.

Funding Sources

This work was supported by Swiss National Science Foundation grant number 407440_183434/1.

Author Contributions

BW, FP, and HV had the original idea. BW, MdRC, MMM and HV provided conceptual and methodological expertise to the study design and BW, FP, CMM, AvG, and HV to data analysis and interpretation. BW, FP, and HV were major contributors to writing the manuscript. All authors read, edited, and approved the final manuscript.

Data Availability Statement

As part of the Data Use Agreement, authors are not allowed to provide raw data. Upon a reasonable request, the corresponding author will provide statistical programming code used to generate results.

Figure Legends

Fig. 1. Relationship between unplanned institutionalization and number of prescribed drugs at discharge.

Fig. 2. Baseline, GEE logistic regression model with unplanned institutionalization as the dependent variable associated with sociodemographic, hospitalization, and independent clinical and medical variables (N = 14,705 observations for 9,430 different subjects).

Fig. 3. The GEE logistic regression model of the drugs prescribed to older adults at discharge with significant predictive values (odds ratios) for unplanned institutionalization (N = 14,705 observations for 9,430 different subjects).

For peer review only

References

1. Tasheva P, Vollenweider P, Kraege V, Roulet G, Lamy O, Marques-Vidal P, et al. Association Between Physical Activity Levels in the Hospital Setting and Hospital-Acquired Functional Decline in Elderly Patients. JAMA Netw Open. 2020;3(1):e1920185.
2. Harrison JK, Garrido AG, Rhynas SJ, Logan G, MacLullich AM, MacArthur J, et al. New institutionalisation following acute hospital admission: a retrospective cohort study. Age Ageing. 2017;46(2):238-44.
3. Gaugler JE, Duval S, Anderson KA, Kane RL. Predicting nursing home admission in the U.S: a meta-analysis. BMC Geriatr. 2007;7:13.
4. Oseran AS, Lage DE, Jernigan MC, Metlay JP, Shah SJ. A "Hospital-Day-1" Model to Predict the Risk of Discharge to a Skilled Nursing Facility. J Am Med Dir Assoc. 2019;20(6):689-95 e5.
5. Huang H-H, Chang JC-Y, Tseng C-C, Yang Y-J, Fan J-S, Chen Y-C, et al. Comprehensive geriatric assessment in the emergency department for the prediction of readmission among older patients: A 3-month follow-up study. Archives of Gerontology and Geriatrics. 2021;92:104255.
6. Middleton A, Li S, Kuo YF, Ottenbacher KJ, Goodwin JS. New Institutionalization in Long-Term Care After Hospital Discharge to Skilled Nursing Facility. J Am Geriatr Soc. 2018;66(1):56-63.
7. Luppa M, Luck T, Weyerer S, Konig HH, Brahler E, Riedel-Heller SG. Prediction of institutionalization in the elderly. A systematic review. Age Ageing. 2010;39(1):31-8.
8. Salminen M, Laine J, Vahlberg T, Viikari P, Wuorela M, Viitanen M, et al. Factors associated with institutionalization among home-dwelling patients of Urgent Geriatric Outpatient Clinic: a 3-year follow-up study. Eur Geriatr Med. 2020;11(5):745-51.
9. Bellelli G, Magnifico F, Trabucchi M. Outcomes at 12 months in a population of elderly patients discharged from a rehabilitation unit. J Am Med Dir Assoc. 2008;9(1):55-64.
10. Fogg C, Griffiths P, Meredith P, Bridges J. Hospital outcomes of older people with cognitive impairment: An integrative review. Int J Geriatr Psychiatry. 2018;33(9):1177-97.
11. Landefeld CS, Palmer RM, Kresevic DM, Fortinsky RH, Kowal J. A randomized trial of care in a hospital medical unit especially designed to improve the functional outcomes of acutely ill older patients. N Engl J Med. 1995;332(20):1338-44.
12. Ferrucci L, Guralnik JM, Pahor M, Corti MC, Havlik RJ. Hospital diagnoses, Medicare charges, and nursing home admissions in the year when older persons become severely disabled. JAMA. 1997;277(9):728-34.
13. Ferrucci L, Bandinelli S, Benvenuti E, Di Iorio A, Macchi C, Harris TB, et al. Subsystems contributing to the decline in ability to walk: bridging the gap between epidemiology and geriatric practice in the InCHIANTI study. J Am Geriatr Soc. 2000;48(12):1618-25.
14. Kasper C. Skeletal muscle atrophy. In: V. C-K, editor. Pathophysiological phenomena in nursing: human responses to illness. St. Louis: Saunders; 2003. p. p. 389-412.
15. Rosenberg T, Montgomery P, Hay V, Lattimer R. Using frailty and quality of life measures in clinical care of the elderly in Canada to predict death, nursing home transfer and hospitalisation - the frailty and ageing cohort study. BMJ Open. 2019;9(11):e032712.
16. Chong E, Ho E, Baldevarona-Llego J, Chan M, Wu L, Tay L, et al. Frailty in Hospitalized Older Adults: Comparing Different Frailty Measures in Predicting Short- and Long-term Patient Outcomes. J Am Med Dir Assoc. 2018;19(5):450-7 e3.
17. Troster T, Thalmann M, Fischer K, Bieri-Bruning G, Beeler PE, Bischoff-Ferrari HA, et al. Frailty, underweight and impaired mobility are associated with institutionalisation after post-acute care. Swiss Med Wkly. 2020;150:w20276.
18. Koirala B, Hansen BR, Hosie A, Budhathoki C, Seal S, Beaman A, et al. Delirium point prevalence studies in inpatient settings: A systematic review and meta-analysis. J Clin Nurs. 2020;n/a(n/a).

For peer review only - http://bmjopen.bmj.com/site/about/guidelines.xhtml

19. Welch C, McCluskey L, Wilson D, Chapman GE, Jackson TA, Treml J, et al. Delirium is prevalent in older hospital inpatients and associated with adverse outcomes: results of a prospective multi-centre study on World Delirium Awareness Day. BMC Medicine. 2019;17(1):229.
20. Zekry D, Herrmann FR, Grandjean R, Vitale AM, De Pinho MF, Michel JP, et al. Does dementia predict adverse hospitalization outcomes? A prospective study in aged inpatients. Int J Geriatr Psychiatry. 2009;24(3):283-91.
21. Haaksma ML, Rizzuto D, Ramakers I, Garcia-Ptacek S, Marengoni A, van der Flier WM, et al. The Impact of Frailty and Comorbidity on Institutionalization and Mortality in Persons With Dementia: A Prospective Cohort Study. J Am Med Dir Assoc. 2019;20(2):165-70 e2.
22. Fried TR, O'Leary J, Towle V, Goldstein MK, Trentalange M, Martin DK. Health outcomes associated with polypharmacy in community-dwelling older adults: a systematic review. Journal of the American Geriatrics Society. 2014;62(12):2261-72.
23. Wimmer BC, Dent E, Visvanathan R, Wiese MD, Johnell K, Chapman I, et al. Polypharmacy and medication regimen complexity as factors associated with hospital discharge destination among older people: a prospective cohort study. Drugs Aging. 2014;31(8):623-30.
24. Koppitz AL, Dreizler J, Altherr J, Bosshard G, Naef R, Imhof L. Relocation experiences with unplanned admission to a nursing home: a qualitative study. Int Psychogeriatr. 2017;29(3):517-27.
25. Pereira F, Roux P, Santiago-Delefosse M, von Gunten A, Wernli B, Martins MM, et al. Optimising medication management for polymedicated home-dwelling older adults with multiple chronic conditions: a mixed-methods study protocol. BMJ Open. 2019;9(10):e030030.
26. Swiss Federal Statistical Office. Swiss Classification of Surgical Procedures (CHOP). 2017. Report No.: 9783303142851.
27. WHO. The Anatomical Therapeutic Chemical Classification System with Defined Daily Doses (ATC/DDD) Geneva: World Health Organization; 2014 [24 March 2020]. Available from: http://www.who.int/classifications/atcddd/en/.
28. Olsen J. Register-based research: some methodological considerations. Scand J Public Health. 2011;39(3):225-9.
29. Taushanov Z, Verloo H, Wernli B, Giovanni SD, Gunten Av, Pereira F. Transforming a Patient Registry Into a Customized Data Set for the Advanced Statistical Analysis of Health Risk Factors and for Medication-Related Hospitalization Research: Retrospective Hospital Patient Registry Study. JMIR Medical Informatics. 2021;9(5).
30. Andrade C. Age as a variable: Continuous or categorical? Indian J Psychiatry. 2017;59(4):524-5.
31. Haider SI, Johnell K, Thorslund M, Fastbom J. Trends in polypharmacy and potential drug-drug interactions across educational groups in elderly patients in Sweden for the period 1992 - 2002. Int J Clin Pharmacol Ther. 2007;45(12):643-53.
32. Yang HZ, Qiao XG, Das S, Paul MC. Thermal regenerated grating operation at temperatures up to 1400 degrees C using new class of multimaterial glass-based photosensitive fiber. Opt Lett. 2014;39(22):6438-41.
33. Goodwin JS, Howrey B, Zhang DD, Kuo YF. Risk of continued institutionalization after hospitalization in older adults. J Gerontol A Biol Sci Med Sci. 2011;66(12):1321-7.
34. Gillès De Pélichy E, Verloo H, Gasser J, Monod S. Intérêts et nécessité du renfort des soins à domicile dans le cadre des situations de crise psychique-Le résultat d'une politique de santé publique forte. Rev Med Suisse. 2020:1741-4.
35. Observatoire valaisan de la santé. Statistique cantonale des établissements médico-sociaux (EMS). OVS; 2020.
36. Yoo JW, Nakagawa S, Kim S. Delirium and transition to a nursing home of hospitalized older adults: a controlled trial of assessing the interdisciplinary team-based "geriatric" care and care coordination by non-geriatrics specialist physicians. Geriatr Gerontol Int. 2013;13(2):342-50.

For peer review only - http://bmjopen.bmj.com/site/about/guidelines.xhtml

37. Del DucaI GF, da Silva SG, ThuméI E, Santos IS, Hallal PC. Predictive factors for institutionalization of the elderly: a case-control study. Rev Saúde Pública 2012;46(1).
38. Luppa M, Luck T, Weyerer S, Konig HH, Riedel-Heller SG. Gender differences in predictors of nursing home placement in the elderly: a systematic review. Int Psychogeriatr. 2009;21(6):1015-25.
39. Gardner RL, Harris F, Vittinghoff E, Cummings SR. The risk of fracture following hospitalization in older women and men. Arch Intern Med. 2008;168(15):1671-7.
40. Cutugno CL. The 'Graying' of Trauma Care: Addressing Traumatic Injury in Older Adults. American Journal of Nursing. 2011;111(11):40-8.
41. Lucchetti G, Granero AL, Pires SL, Gorzoni ML. Fatores associados à polifarmácia em idosos institucionalizados. Revista Brasileira de Geriatria e Gerontologia. 2010;13:51-8.
42. Castilho ECD, Reis A, Borges T, Siqueira L, Miasso A. Potential drug–drug interactions and polypharmacy in institutionalized elderly patients in a public hospital in Brazil. Journal of psychiatric and mental health nursing. 2018;25(1):3-13.
43. de Oliveira LM, Diel JdAC, Nunes A, Dal Pizzol TdS. Prevalence of drug interactions in hospitalised elderly patients: a systematic review. European Journal of Hospital Pharmacy. 2021;28(1):4-9.
44. Montes Reula L, Cañete Lairla M, Navarro López J, Pelegrín Valero C, Galindo Ortiz de Landázuri J, Marijuán Fernández P, et al. Predominant factors of institutionalization in the elderly: a comparative study between home nursing and community dwelling2020.
45. Carvalho TC, Valle APd, Jacinto AF, Mayoral VFdS, Boas PJFV. Impact of hospitalization on the functional capacity of the elderly: A cohort study. Revista Brasileira de Geriatria e Gerontologia. 2018;21(2):134-42.
46. Covinsky KE, Pierluissi E, Johnston CB. Hospitalization-associated disability:"She was probably able to ambulate, but I'm not sure". Jama. 2011;306(16):1782-93.

For peer review only - http://bmjopen.bmj.com/site/about/guidelines.xhtml

Table 1. Prevalence of unplanned institutionalizations with regards to associations with sociodemographic characteristics and clinical and medical conditions among polymedicated hospitalized older adults (N = 14,705)

**Variables**	**Unplanned institutionalization %**	***p*-value**
Overall sample of older adults (n=14,705)	6.1% (n= 897)	
Sex		$p < 0.001$
Female/Male	8.8%/4.0%	
Age in years		$p < 0.001$
65–69 years	2.2%	
70–79 years	3.2%	
80–89 years	8.3%	
90 years or more	19.7%	
Mobility		$p < 0.001$
Full ability (0) / impairment (1)	2.0%/16.7%	
Dependence in the activities of daily living		$p < 0.001$
Full ability (0)/impairment (1)	3.4%/44.8%	
Mental status		$p < 0.001$
Full ability (0)/impairment (1)	3.8%/41.3%	
ICD-10 principal diagnosis: circulatory problems		$p < 0.001$
No (0)/Yes (1)	6.7%/4.3%	
ICD-10 principal diagnosis: infection		$p < 0.01$
No (0)/Yes (1)	6.2%/2.7%	
ICD-10 principal diagnosis: respiratory problems		Ns
No (0)/Yes (1)	6.1%/6.8%	
ICD-10 principal diagnosis: traumatic injuries		$p < 0.001$
No (0)/Yes (1)	5.3%/14.9%	
ICD-10 principal diagnosis: tumor		$p < 0.01$
No (0)/Yes (1)	6.4%/4.3%	
Number of ICD-10 diseases		$p < 0.001$
1	1.8%	
2	2.9%	
3	3.9%	
4	3.9%	
5 or more	6.8%	
Number of surgical interventions (CHOP)		$p < 0.001$
0	7.8%	
1	6.4%	
2	5.8%	
3	5.2%	
4	3.5%	
5 or more	4.2%	
Year of hospitalization		$p < 0.01$
2015/2016/2017/2018	7.3%/6.1%/5.2%/5.9%	

Note. Ns = non significant

For peer review only

For peer review only - http://bmjopen.bmj.com/site/about/guidelines.xhtml

589 Table 2. Prevalence of unplanned institutionalization among polymedicated hospitalized older adults
590 (N = 14,705) with regards to associations with different classes of prescribed drugs
591

Drugs (ATC code)	Unplanned institutionalization		
	**No drugs in this class %**	**Drugs in this class %**	***p*-value**
First level, anatomical main group			
Blood and blood-forming organ drugs (B)	5.4%	6.4%	ns
Dermatologicals (D)	5.8%	14.1%	$p < 0.001$
Genito-urinary system and sex hormones (G)	6.1%	6.3%	ns
Systemic hormonal preparations, excluding sex hormones and insulins (H)	6.1%	6.5%	ns
Anti-infectives for systemic use (J)	6.4%	5.3%	$p < 0.05$
Antineoplastic and immunomodulating agents (L)	6.3%	3.5%	$p < 0.01$
Drugs for the musculoskeletal system (M)	6.4%	4.3%	$p < .001$
Antiparasitic products, insecticides, and repellents (P)	6.2%	4.0%	Ns
Respiratory system drugs (R)	6.3%	5.5%	Ns
Sensory organ drugs (S)	5.5%	13.4%	$p < 0.001$
Second level, therapeutic subgroup			
Stomatological preparations (A01)	6.1%	7.5%	ns
Drugs for acid-related disorders (A02)	5.8%	6.4%	ns
Drugs for functional gastrointestinal disorders (A03	5.9%	9.8%	$p < 0.001$
Antiemetics and antinauseants (A04)	6.1%	18.6%	$p < 0.001$
Bile and liver therapy drugs (A05)	6.1%	7.9%	ns
Drugs for constipation (A06)	4.8%	13.5%	$p < 0.001$
Antidiarrheals, intestinal anti-inflammatory/anti-infective agents (A07)	6.0%	9.4%	$p < 0.01$
Digestives, including enzymes (A09)	6.1%	8.4%	Ns
Diabetes drugs (A10)	6.6%	3.9%	$p < 0.001$
Vitamins (A11)	6.2%	5.9%	ns
Mineral supplements (A12)	4.8%	9.6%	$p < 0.001$
Other alimentary tract and metabolism products (A16)	6.1%	5.9%	ns
Cardiac therapy drugs (C01)	6.1%	6.3%	ns
Antihypertensives (C02)	6.2%	4.6%	ns
Diuretics (C03)	5.5%	8.1%	$p < 0.001$
Peripheral vasodilators (C04)	6.1%	4.2%	ns
Vasoprotectives (C05)	6.1%	7.2%	ns
Beta blocking agents (C07)	7.2%	4.8%	$p < 0.001$
Calcium channel blockers (C08)	6.1%	6.1%	ns
Agents acting on the renin-angiotensin system (C09)	7.2%	5.3%	$p < 0.001$
Lipid-modifying agents (C10)	8.2%	3.1%	$p < 0.001$
Anesthetics (N01)	6.1%	13.5%	ns
Analgesics (N02)	3.6%	7.2%	$p < 0.001$
Antiepileptics (N03)	5.7%	10.3%	$p < 0.001$
Drugs against Parkinson's disease (N04)	5.7%	18.1%	$p < 0.001$
Psycholeptics (N05)	2.4%	11.0%	$p < 0.001$
Psychoanaleptics (N06)	4.8%	11.9%	$p < 0.001$
Other nervous system drugs (N07)	6.1%	5.9%	ns

592 Note. Ns = non significant

For peer review only - http://bmjopen.bmj.com/site/about/guidelines.xhtml

Table 3. Factors associated with a higher probability of unplanned institutionalization among polymedicated hospitalized older adults (N = 14,705): summary of the predictive analysis.

**Determinants of a higher probability of unplanned institutionalization (risk factors)**
- Dependency in the activities of daily living (OR = 4.62, 95% CI: 3.76–5.67)
- Cognitive impairment (OR = 3.75, 95% CI: 3.06–4.59)
- Functional mobility impairment (OR = 3.22, 95% CI: 2.67–3.87)
- Antiemetics/antinauseants (OR = 2.53, 95% CI: 1.21–5.30)
- Digestives (OR = 1.78, 95% CI: 1.09–2.90)
- Psycholeptics (OR = 1.76, 95% CI: 1.60–1.93)
- Traumatic injuries (OR = 1.58, 95% CI: 1.25–2.01)
- Antiepileptics (OR = 1.49, 95% CI: 1.25–1.79)
- Anti-Parkinson's drugs (OR = 1.40, 95% CI: 1.12–1.75)
- Number of prescribed drugs (OR = 1.17, 95% CI: 1.15–1.19)
- Older age (OR = 1.07, 95% CI: 1.05–1.08)
Combined intake of:<br>- cardiac and psychoanaleptic drugs (OR = 1.87, 95% CI: 1.11–3.16)<br>- psychoanaleptic and diabetes drugs (OR = 1.75, 95% CI: 1.03–2.98)<br>- psycholeptic drugs and vitamins (OR = 1.71, 95% CI: 1.03–2.84)
Combined intake of two or more:<br>- antiemetics and antinauseants (OR = 2.65, 95% CI: 1.26–5.58)<br>- psycholeptics (OR = 1.64, 95% CI: 1.46–1.85)<br>- antiepileptics (OR = 1.55, 95% CI: 1.23–1.96)<br>- anti-Parkinson's drugs (OR = 1.44, 95% CI: 1.13–1.83)
**Determinants of a lower probability of unplanned institutionalization (protective factors)**
- Surgical interventions (OR = 0.95, 95% CI: 0.90–0.99)
- Circulatory diseases (OR = 0.78, 95% CI: 0.63–0.98)
- Lipid metabolism modifying agents (OR = 0.73, 95% CI: 0.60–0.90)
- Male sex (OR = 0.62; 95% CI: 0.52–0.73)
- Combined intake of beta-blocking agents and antiepileptics (OR = 0.39, 95% CI: 0.23–0.67)
- Infectious diseases (OR = 0.38, 95% CI: 0.20–0.70)

For peer review only - http://bmjopen.bmj.com/site/about/guidelines.xhtml

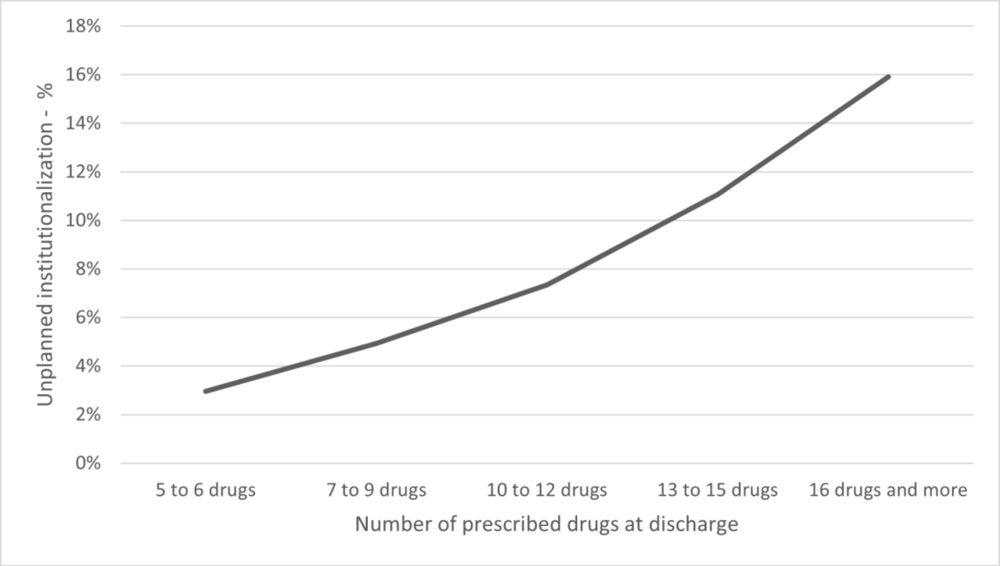

Relationship between unplanned institutionalization and number of prescribed drugs at discharge.

386x219mm (130 x 130 DPI)

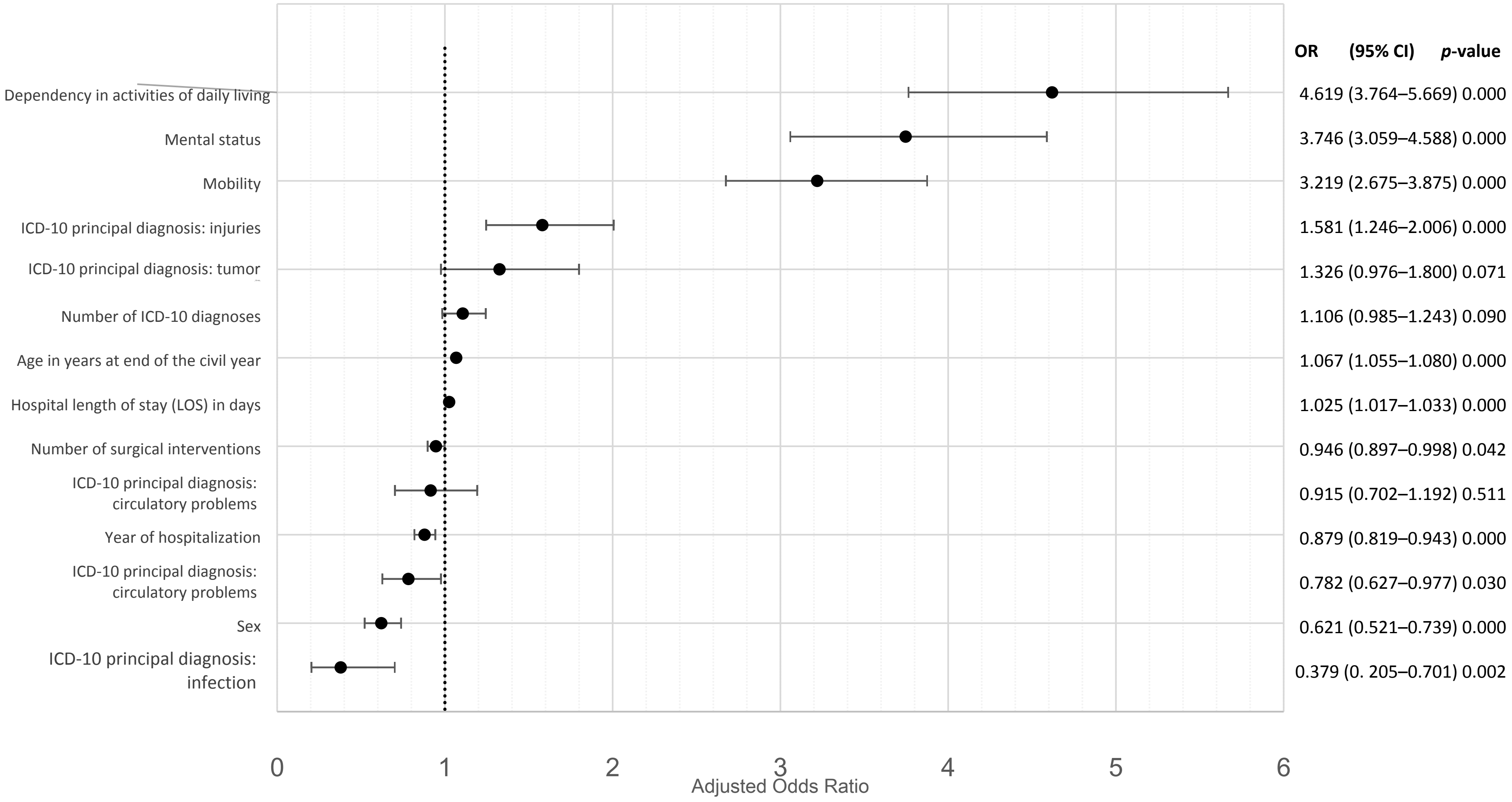
OR (95% CI) p-value
Dependency in activities of daily living
4.619 (3.764–5.669) 0.000
Mental status
3.746 (3.059–4.588) 0.000
Mobility
3.219 (2.675–3.875) 0.000
ICD-10 principal diagnosis: injuries
1.581 (1.246–2.006) 0.000
ICD-10 principal diagnosis: tumor
1.326 (0.976–1.800) 0.071
Number of ICD-10 diagnoses
1.106 (0.985–1.243) 0.090
Age in years at end of the civil year
1.067 (1.055–1.080) 0.000
Hospital length of stay (LOS) in days
1.025 (1.017–1.033) 0.000
Number of surgical interventions
0.946 (0.897–0.998) 0.042
ICD-10 principal diagnosis: circulatory problems
0.915 (0.702–1.192) 0.511
Year of hospitalization
0.879 (0.819–0.943) 0.000
ICD-10 principal diagnosis: circulatory problems
0.782 (0.627–0.977) 0.030
Sex
0.621 (0.521–0.739) 0.000
ICD-10 principal diagnosis: infection
0.379 (0. 205–0.701) 0.002
0
1
2
3
4
5
6
Adjusted Odds Ratio

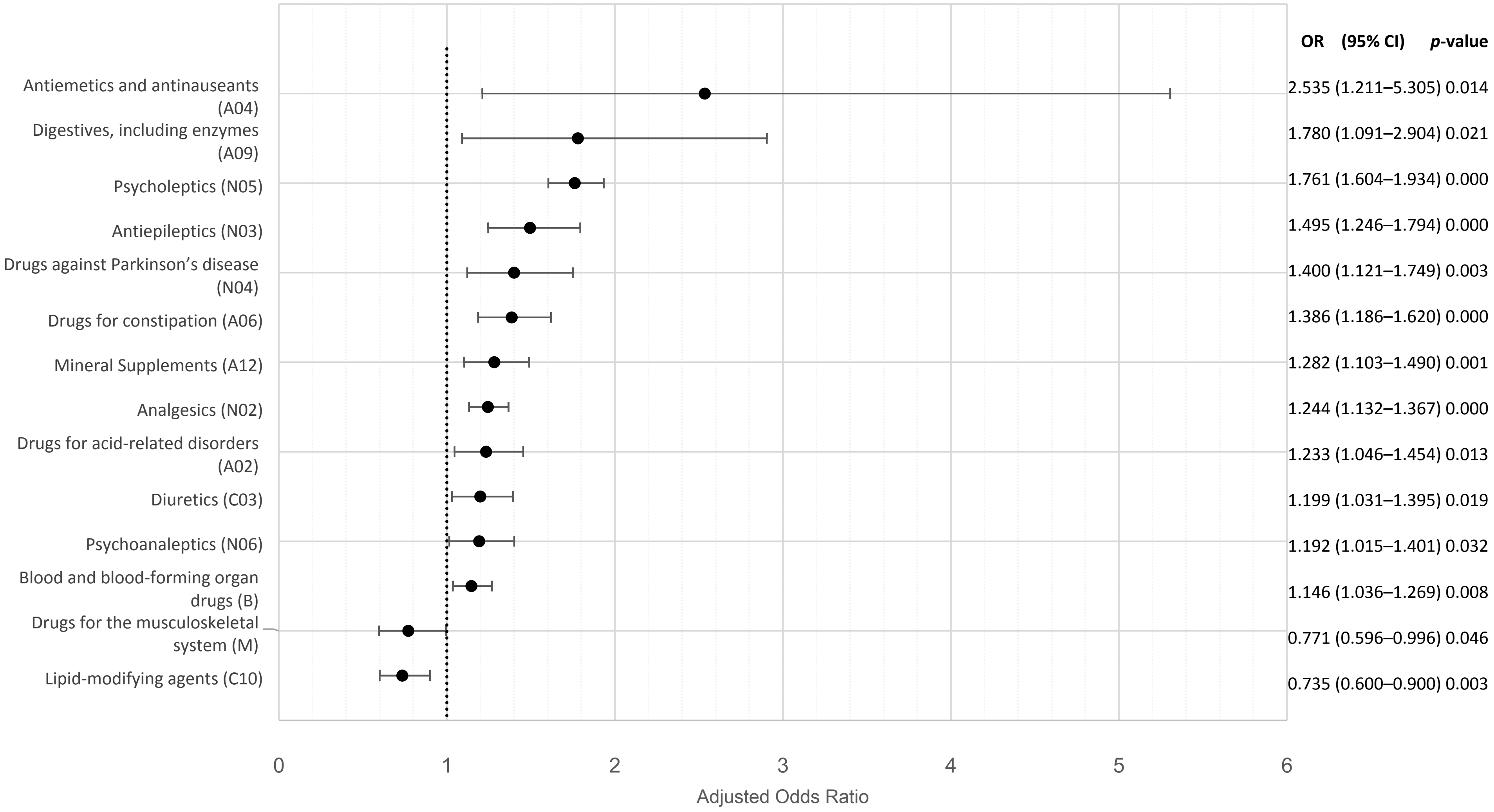
OR (95% CI) p-value
Antiemetics and antinauseants (A04)
2.535 (1.211–5.305) 0.014
Digestives, including enzymes (A09)
1.780 (1.091–2.904) 0.021
Psycholeptics (N05)
1.761 (1.604–1.934) 0.000
Antiepileptics (N03)
1.495 (1.246–1.794) 0.000
Drugs against Parkinson’s disease (N04)
1.400 (1.121–1.749) 0.003
Drugs for constipation (A06)
1.386 (1.186–1.620) 0.000
Mineral Supplements (A12)
1.282 (1.103–1.490) 0.001
Analgesics (N02)
1.244 (1.132–1.367) 0.000
Drugs for acid-related disorders (A02)
1.233 (1.046–1.454) 0.013
Diuretics (C03)
1.199 (1.031–1.395) 0.019
Psychoanaleptics (N06)
1.192 (1.015–1.401) 0.032
Blood and blood-forming organ drugs (B)
1.146 (1.036–1.269) 0.008
Drugs for the musculoskeletal system (M)
0.771 (0.596–0.996) 0.046
Lipid-modifying agents (C10)
0.735 (0.600–0.900) 0.003
0
1
2
3
4
5
6
Adjusted Odds Ratio

Supplementary Table 1. Descriptive statistics of prescribed drugs at discharge based on the ATC among the polymedicated older inpatients (N = 14,705)

**Drugs by ATC, level 2**	**Number of drugs per patient**	
	**Min-Max**	**Mean (S.D.)**
First level, anatomical main group		
Blood and blood-forming organ drugs (B)	0-6	1.16 (0.86)
Dermatologicals (D)	0-3	0.04 (0.22)
Genito urinary system and sex hormones (G)	0-4	0.21 (0.47)
Systemic hormonal preparations, excl. sex hormones and insulins (H)	0-4	0.20 (0.46)
Anti-infective for systemic use (J)	0-4	0.23 (0.46)
Antineoplastic and immunomodulating agents (L)	0-5	0.05 (0.23)
Musculo skeletal system drugs (M)	0-3	0.15 (0.39)
Antiparasitic products, insecticides and repellents (P)	0-2	0.02 (0.13)
Respiratory system drugs (R)	0-7	0.27 (0.72)
Sensory organ drugs (S)	0-6	0.10 (0.40)
Second level, therapeutic subgroup		
Stomatological preparations (A01)	0-1	0.01 (0.06)
Drugs for acid related disorders (A02)	0-3	0.56 (0.52)
Drugs for functional gastrointestinal disorders (A03)	0-3	0.07 (0.28)
Antiemetics and antinauseants (A04)	0-1	0.01 (0.08)
Bille and liver therapy drugs (A05)	0-1	0.01 (0.05)
Drugs for constipation (A06)	0-4	0.17 (0.42)
Antidiarrheals, intestinal antiinflammatory/antiinfective agents (A07)	0-2	0.03 (0.18)
Digestives, incl. enzymes (A09)	0-2	0.02 (0.13)
Drugs used in diabetes (A10)	0-5	0.25 (0.63)
Vitamins (A11)	0-4	0.15 (0.44)
Mineral supplements (A12)	0-3	0.30 (0.51)
Other alimentary tract and metabolism products (A16)	0-1	0.01 (0.05)
Cardio-therapy drugs (C01)	0-4	0.14 (0.41)
Antihypertensives (C02)	0-2	0.02 (0.17)
Diuretics (C03)	0-3	0.28 (0.54)
Peripheral vasodilators (C04)	0-1	0.01 (0.06)
Vaso-protectives (C05)	0-3	0.02 (0.14)
Beta-blocking agents (C07)	0-2	0.45 (0.51)
Calcium channel blockers (C08)	0-2	0.16 (0.37)
Agents acting on the Renin-Angiotensin system (C09)	0-3	0.63 (0.62)
Lipid Modifying agents (C10)	0-3	0.41 (0.52)
Anesthetics (N01)	0-1	0.01 (0.05)
Analgesics (N02)	0-7	1.03 (0.91)
Antiepileptics (N03)	0-5	0.11 (0.36)
Anti-Parkinson drugs (N04)	0-5	0.04 (0.25)
Psycholeptics (N05)	0-7	0.57 (0.77)
Psychoanaleptics (N06)	0-3	0.21 (0.45)
Other nervous system drugs (N07)	0-3	0.03 (0.19)
Total number of drugs	5-32	9.07 (3.32)
N valid - listwise		14,70

For peer review only - http://bmjopen.bmj.com/site/about/guidelines.xhtml

The RECORD statement – checklist of items, extended from the STROBE statement, that should be reported in observational studies using routinely collected health data.

	Item No.	STROBE items	Location in manuscript where items are reported	RECORD items	Location in manuscript where items are reported
**Title and abstract**					
	1	(a) Indicate the study's design with a commonly used term in the title or the abstract (b) Provide in the abstract an informative and balanced summary of what was done and what was found		RECORD 1.1: The type of data used should be specified in the title or abstract. When possible, the name of the databases used should be included.<br><br>RECORD 1.2: If applicable, the geographic region and timeframe within which the study took place should be reported in the title or abstract.<br><br>RECORD 1.3: If linkage between databases was conducted for the study, this should be clearly stated in the title or abstract.	Title<br>Abstract (line 34)<br><br>Line 6<br><br>Lines 35<br><br>Not applicable, only one hospital register
**Introduction**					
Background rationale	2	Explain the scientific background and rationale for the investigation being reported			Lines 75-148
Objectives	3	State specific objectives, including any prespecified hypotheses			Lines 145-148
**Methods**					
Study Design	4	Present key elements of study design early in the paper			Lines 151-153
Setting	5	Describe the setting, locations, and relevant dates, including periods of recruitment, exposure, follow-up, and data collection			Lines 155-177

For peer review only

Participants	6	*(a) Cohort study* - Give the eligibility criteria, and the sources and methods of selection of participants. Describe methods of follow-up<br>*Case-control study* - Give the eligibility criteria, and the sources and methods of case ascertainment and control selection. Give the rationale for the choice of cases and controls<br>*Cross-sectional study* - Give the eligibility criteria, and the sources and methods of selection of participants<br><br>*(b) Cohort study* - For matched studies, give matching criteria and number of exposed and unexposed<br>*Case-control study* - For matched studies, give matching criteria and the number of controls per case		RECORD 6.1: The methods of study population selection (such as codes or algorithms used to identify subjects) should be listed in detail. If this is not possible, an explanation should be provided.<br><br>RECORD 6.2: Any validation studies of the codes or algorithms used to select the population should be referenced. If validation was conducted for this study and not published elsewhere, detailed methods and results should be provided.<br><br>RECORD 6.3: If the study involved linkage of databases, consider use of a flow diagram or other graphical display to demonstrate the data linkage process, including the number of individuals with linked data at each stage.	Reported in a previous study: https://pubmed.ncbi.nlm.nih.gov/33973865/<br><br>Reported in a previous study: https://pubmed.ncbi.nlm.nih.gov/33973865/<br><br>Not applicable, only one hospital register
Variables	7	Clearly define all outcomes, exposures, predictors, potential confounders, and effect modifiers. Give diagnostic criteria, if applicable.		RECORD 7.1: A complete list of codes and algorithms used to classify exposures, outcomes, confounders, and effect modifiers should be provided. If these cannot be reported, an explanation should be provided.	Lines 181-222 and reported in a previous study: https://pubmed.ncbi.nlm.nih.gov/33973865/
Data sources/ measurement	8	For each variable of interest, give sources of data and details of methods of assessment (measurement).			Lines 181-222 and reported in a previous study:

For peer review only

		Describe comparability of assessment methods if there is more than one group			https://pubmed.ncbi.nlm.nih.gov/33973865/
Bias	9	Describe any efforts to address potential sources of bias			Lines 224-241 and reported in a previous study: https://pubmed.ncbi.nlm.nih.gov/33973865/
Study size	10	Explain how the study size was arrived at			Lines 155-177
Quantitative variables	11	Explain how quantitative variables were handled in the analyses. If applicable, describe which groupings were chosen, and why			Lines 224-241 and reported in a previous study: https://pubmed.ncbi.nlm.nih.gov/33973865/
Statistical methods	12	(a) Describe all statistical methods, including those used to control for confounding<br>(b) Describe any methods used to examine subgroups and interactions<br>(c) Explain how missing data were addressed<br>(d) *Cohort study* - If applicable, explain how loss to follow-up was addressed<br>*Case-control study* - If applicable, explain how matching of cases and controls was addressed<br>*Cross-sectional study* - If applicable, describe analytical			Lines 224-241

For peer review only

		methods taking account of sampling strategy<br>(e) Describe any sensitivity analyses			
Data access and cleaning methods		..		RECORD 12.1: Authors should describe the extent to which the investigators had access to the database population used to create the study population.<br><br>RECORD 12.2: Authors should provide information on the data cleaning methods used in the study.	Lines 224-241 and reported in a previous study: https://pubmed.ncbi.nlm.nih.gov/33973865/
Linkage		..		RECORD 12.3: State whether the study included person-level, institutional-level, or other data linkage across two or more databases. The methods of linkage and methods of linkage quality evaluation should be provided.	Reported in a previous study: https://pubmed.ncbi.nlm.nih.gov/33973865/
**Results**					
Participants	13	(a) Report the numbers of individuals at each stage of the study (*e.g.*, numbers potentially eligible, examined for eligibility, confirmed eligible, included in the study, completing follow-up, and analysed)<br>(b) Give reasons for non-participation at each stage.<br>(c) Consider use of a flow diagram		RECORD 13.1: Describe in detail the selection of the persons included in the study (*i.e.,* study population selection) including filtering based on data quality, data availability and linkage. The selection of included persons can be described in the text and/or by means of the study flow diagram.	Lines 170-177
Descriptive data	14	(a) Give characteristics of study participants (*e.g.*, demographic, clinical, social) and information on exposures and potential confounders			Lines 243-264

For peer review only - http://bmjopen.bmj.com/site/about/guidelines.xhtml

		(b) Indicate the number of participants with missing data for each variable of interest<br>(c) *Cohort study* - summarise follow-up time (*e.g.*, average and total amount)			
Outcome data	15	*Cohort study* - Report numbers of outcome events or summary measures over time<br>*Case-control study* - Report numbers in each exposure category, or summary measures of exposure<br>*Cross-sectional study* - Report numbers of outcome events or summary measures			Lines 243-264
Main results	16	(a) Give unadjusted estimates and, if applicable, confounder-adjusted estimates and their precision (e.g., 95% confidence interval). Make clear which confounders were adjusted for and why they were included<br>(b) Report category boundaries when continuous variables were categorized<br>(c) If relevant, consider translating estimates of relative risk into absolute risk for a meaningful time period			Lines 277-327
Other analyses	17	Report other analyses done—e.g., analyses of subgroups and interactions, and sensitivity analyses			Lines 268-280
**Discussion**					
Key results	18	Summarise key results with reference to study objectives			Lines 339-342

For peer review only

Limitations	19	Discuss limitations of the study, taking into account sources of potential bias or imprecision. Discuss both direction and magnitude of any potential bias		RECORD 19.1: Discuss the implications of using data that were not created or collected to answer the specific research question(s). Include discussion of misclassification bias, unmeasured confounding, missing data, and changing eligibility over time, as they pertain to the study being reported.	Lines 405-421
Interpretation	20	Give a cautious overall interpretation of results considering objectives, limitations, multiplicity of analyses, results from similar studies, and other relevant evidence			Lines 333-394
Generalisability	21	Discuss the generalisability (external validity) of the study results			Lines 396-400
**Other Information**					
Funding	22	Give the source of funding and the role of the funders for the present study and, if applicable, for the original study on which the present article is based			Line 451
Accessibility of protocol, raw data, and programming code		..		RECORD 22.1: Authors should provide information on how to access any supplemental information such as the study protocol, raw data, or programming code.	Lines 185, 188, 200-201<br><br>https://pubmed.ncbi.nlm.nih.gov/33973865/

*Reference: Benchimol EI, Smeeth L, Guttmann A, Harron K, Moher D, Petersen I, Sørensen HT, von Elm E, Langan SM, the RECORD Working Committee. The REporting of studies Conducted using Observational Routinely-collected health Data (RECORD) Statement. *PLoS Medicine* 2015; in press.

*Checklist is protected under Creative Commons Attribution (CC BY) license.

BMJ Open

Unplanned nursing home admission among discharged polymedicated older inpatients: a single-centre, registry-based study in Switzerland

Journal:	*BMJ Open*
Manuscript ID	bmjopen-2021-057444.R1
Article Type:	Original research
Date Submitted by the Author:	07-Dec-2021
Complete List of Authors:	Pereira, Filipa; University of Porto, Institute of Biomedical Sciences Abel Salazar; HES-SO Valais Wallis, School of Health Sciences<br>Verloo, Henk; HES-SO Valais Wallis, School of Health Sciences<br>von Gunten, Armin; Centre Hospitalier Universitaire Vaudois Département de Psychiatrie<br>del Río Carral, María; University of Lausanne, Institute of Psychology, Research Center for the Psychology of Health, Aging and Sports Examination<br>Meyer-Massetti, Carla; University of Bern, Institute for Primary Health Care (BIHAM)<br>Martins, Maria Manuela; Escola Superior de Enfermagem do Porto, Formação&gestão<br>Wernli, Boris; University of Lausanne, FORS, Swiss Centre of Expertise in the Social Sciences
**Primary Subject Heading**:	Geriatric medicine
Secondary Subject Heading:	Pharmacology and therapeutics, Health informatics
Keywords:	GERIATRIC MEDICINE, CLINICAL PHARMACOLOGY, Health informatics < BIOTECHNOLOGY & BIOINFORMATICS, EPIDEMIOLOGY

SCHOLARONE™
Manuscripts

I, the Submitting Author has the right to grant and does grant on behalf of all authors of the Work (as defined in the below author licence), an exclusive licence and/or a non-exclusive licence for contributions from authors who are: i) UK Crown employees; ii) where BMJ has agreed a CC-BY licence shall apply, and/or iii) in accordance with the terms applicable for US Federal Government officers or employees acting as part of their official duties; on a worldwide, perpetual, irrevocable, royalty-free basis to BMJ Publishing Group Ltd ("BMJ") its licensees and where the relevant Journal is co-owned by BMJ to the co-owners of the Journal, to publish the Work in this journal and any other BMJ products and to exploit all rights, as set out in our licence.

The Submitting Author accepts and understands that any supply made under these terms is made by BMJ to the Submitting Author unless you are acting as an employee on behalf of your employer or a postgraduate student of an affiliated institution which is paying any applicable article publishing charge ("APC") for Open Access articles. Where the Submitting Author wishes to make the Work available on an Open Access basis (and intends to pay the relevant APC), the terms of reuse of such Open Access shall be governed by a Creative Commons licence – details of these licences and which Creative Commons licence will apply to this Work are set out in our licence referred to above.

Other than as permitted in any relevant BMJ Author's Self Archiving Policies, I confirm this Work has not been accepted for publication elsewhere, is not being considered for publication elsewhere and does not duplicate material already published. I confirm all authors consent to publication of this Work and authorise the granting of this licence.

For peer review only

Unplanned nursing home admission among discharged polymedicated older inpatients: a single-centre, registry-based study in Switzerland

Filipa Pereira[1,2], Henk Verloo[1,3], Armin von Gunten[c], María del Río Carral[4], Carla Meyer-Massetti[5], Maria Manuela Martins[6], Boris Wernli[7]

[1] School of Health Sciences, HES-SO Valais/ Wallis, University of Applied Sciences and Arts Western Switzerland, Sion, Switzerland

[2] Institute of Biomedical Sciences Abel Salazar, University of Porto, Porto, Portugal

[3] Service of Old Age Psychiatry, Lausanne University Hospital, Lausanne, Switzerland

[4] Institute of Psychology, Research Center for the Psychology of Health, Aging and Sports Examination, University of Lausanne, Lausanne, Switzerland

[5] Institute for Primary Health Care (BIHAM), University of Bern, Bern, Switzerland

[6] Higher School of Nursing of Porto, Porto, Portugal

[7] FORS, Swiss Centre of Expertise in the Social Sciences, University of Lausanne, Switzerland

Corresponding Author:

Filipa Pereira

School of Health Sciences, HES-SO Valais / Wallis

University of Applied Sciences and Arts Western Switzerland

5 Chemin de l'Agasse, CH-1950 Sion, Switzerland

Institute of Biomedical Sciences Abel Salazar, University of Porto

228 Rua de Jorge Viterbo Ferreira, 4050-313 Porto, Portugal

Phone: +41 78 666 17 00

Email: filipa.pereira@hevs.ch

ORCID: https://orcid.org/0000-0001-9207-4856

For peer review only

For peer review only - http://bmjopen.bmj.com/site/about/guidelines.xhtml

Abstract

Objective: To investigate patient characteristics and the available health and drug data associated with unplanned nursing home admission following an acute hospital admission or readmission.

Design: A population-based hospital registry study.

Setting: A public hospital in southern Switzerland (Valais Hospital).

Participants: We explored a population-based longitudinal dataset of 14,705 hospital admissions from 2015–2018.

Outcome measures: Sociodemographic, health and drug data, and their interactions predicting the risk of unplanned nursing home admission.

Results: The mean prevalence of unplanned nursing home admission after hospital discharge was 6.1%. Our predictive analysis revealed that the oldest adults (OR = 1.07 for each additional year of age; 95%CI 1.05 to 1.08) presenting with impaired functional mobility (OR = 3.22; 95%CI 2.67 to 3.87), dependency in the activities of daily living (OR = 4.62; 95%CI 3.76 to 5.67), cognitive impairment (OR=3.75; 95%CI 3.06 to 4.59), and traumatic injuries (OR=1.58; 95%CI 1.25 to 2.01) had a higher probability of unplanned nursing home admission. The number of ICD-10 diagnoses had no significant impact on nursing home admissions, contrarily to the number of prescribed drugs (OR=1.17; 95%CI 1.15 to 1.19). Antiemetics/antinauseants (OR=2.53; 95%CI 1.21 to 5.30), digestives (OR=1.78; 95%CI 1.09 to 2.90), psycholeptics (OR=1.76; 95%CI 1.60 to 1.93), antiepileptics (OR=1.49; 95%CI 1.25 to 1.79) and anti-Parkinson's drugs (OR=1.40; 95%CI 1.12 to 1.75) were strongly linked to unplanned nursing home admission.

Conclusions: Numerous risk factors for unplanned nursing home admission were identified. To prevent the adverse health outcomes that precipitate acute hospitalisations and unplanned nursing home admissions, ambulatory-care providers should consider these risk factors in their care planning for older adults before they reach a state requiring hospitalisation.

Keywords: population-based sample; functional decline; hospital discharge; risk factors; nursing home

Strengths and limitations of this study:

For peer review only - http://bmjopen.bmj.com/site/about/guidelines.xhtml

- A hospital registry of 14,705 hospital admissions, involving 9,430 different polymedicated older adults admitted from their homes, was analysed to determine the risk of unplanned nursing home admission.
- Bivariate analyses were conducted on independent variables, and generalised estimating equations were computed to predict how sets of predictors influenced the probability of unplanned nursing home admission.
- Causality analysis was not feasible based on the nature of the routinely collected data.
- Although the study considered statistical associations between drugs and unplanned nursing home admission, it did not use clinically diagnosed drug–drug interactions.
- Our data were unable to identify hospitalisations that might have been triggered by limited home-care options or those that became necessary while older adults awaited a place in anursing home.

Introduction

The hospitalisation of home-dwelling older adults, for any reason and even for a short admission, can lead to substantial functional decline [1, 2]. Both their health disorder itself and the hospital environment can foster such functional decline, increase the risk of future illness and irreversibly diminish their quality of life [1, 2]. Most hospitalised older adult inpatients wish to return home and continue their everyday life as before. However, these different factors may hinder this wish at discharge [3, 4]. The unmet patient needs related to functional decline and safety after returning home can lead to a higher risk of hospital and emergency department readmissions and thus to subsequent unplanned nursing home admission [5]. After hospitalisation, an unplanned nursing home admission can be a devastating and overwhelming experience for older adults and their relatives, and it increases overall healthcare system costs [6].

Whether planned or unplanned, nursing home admission commonly follows two paths: (i) within the community, directly from home, or (ii) from hospital, directly transitioning from hospital discharge [2]. In the community, transitions to nursing homes are generally the result of thoughtful decisions made by home-dwelling older adults, their families, and health- and social-care providers based on the evolution of the person's long-term health and functional state or on an acute decline and corresponding increase in care needs that cannot be met at home. Recent findings have suggested that the predictors of nursing home admission are mainly based on underlying cognitive and functional impairments combined with a lack of support and assistance in daily living at home [7].

For peer review only - http://bmjopen.bmj.com/site/about/guidelines.xhtml

The causes of unplanned nursing home admission directly after acute hospital discharge are heterogeneous. There are several reasons why older adults may require long-term care—that cannot be provided in a community setting—following acute hospital admission, e.g. a new medical problem or the worsening of existing chronic disease(s) entailing dependency and requiring complex forms of care. Furthermore, there may be a breakdown of family circumstances and/or lack of social support.

Bellelli *et al*. showed that advanced age (OR = 4.8; 95% CI 2.6 to 8.9, $p < 0.001$), cognitive impairment (OR = 2.3; 95% CI 1.4 to 3.9, $p < 0.001$) and poor functional status (OR = 10.2; 95% CI 4.7 to 22.5, $p < 001$) at discharge from a rehabilitation unit were the main predictors of subsequent nursing home admission [8]. The integrative review by Fogg *et al*. found a similar result for cognitive impairment (OR = 2.14; 95% CI 1.24 to 3.70, $p < 0.001$) [9]. A randomised controlled trial by Landefeld *et al*. found that older inpatients in an acute care medical unit with a decline in their ability to perform one or more of the basic activities of daily living (ADL) were more often discharged to a nursing home than those with less functional decline (22% and 14 %, respectively; $p < 0.01$) [10]. Ferrucci *et al*. identified stroke, cancer, congestive heart failure, pneumonia, coronary heart disease and hip fractures as the leading medical precipitators of functional decline and nursing home admission [11]. Older adult inpatients are frequently subject to iatrogenic events during hospitalisation, including adverse drug reactions, nosocomial infections, and the consequences of falls, fractures, and using chemical or physical restraints [12]. Such events can lengthen hospitalisation, produce cognitive changes and lessen the ability to perform the ADL, all potentially leading to unplanned nursing home admission [12]. Indeed, hospitalisation causes an increased risk of the onset of acute cognitive decline in the form of delirium, with a prevalence of up to 60% on some surgical wards [13], often leading to unplanned nursing home admission [14]. Dementia, Parkinson's disease and its associated risk of falls, and behavioural changes are common reasons for deciding to transfer inpatients from hospital to long-term care [15, 16].

Polypharmacy has been associated with adverse health outcomes among home-dwelling older adults [17]. Some prospective studies with small samples have established relationships between drug treatments during acute hospitalisation and unplanned nursing home admission [18]. Cardiovascular drugs (particularly vasodilators, diuretics and anticoagulants), drugs against diabetes, steroids, non-steroidal anti-inflammatory drugs, opiates, antibiotics, anticholinergics and benzodiazepines have all been associated with unplanned nursing home admission [18].

To the best of our knowledge, and despite more frequent post-discharge nursing home admissions in Switzerland than in other countries, there is scarce research exploring how unplanned admissions to nursing homes are related to prior hospitalisation [19]. The present study aimed to investigate the

For peer review only - http://bmjopen.bmj.com/site/about/guidelines.xhtml

associations between polymedicated older inpatients' sociodemographic and clinical characteristics, drug data and their interactions, and their unplanned nursing home admission following an acute care hospital stay.

For peer review only

For peer review only - http://bmjopen.bmj.com/site/about/guidelines.xhtml

Materials and Methods

Study design

The present population-based hospital registry study was conducted with close regard to the REporting of studies Conducted using Observational Routinely collected health Data (RECORD) statement.

Population and data collection

Our four-year, longitudinal, population-based hospital registry of electronic health records included polymedicated (five and more drugs prescribed) home-dwelling older adults admitted and readmitted to the Valais hospital, a multisite public teaching hospital (1,074 beds) in southern Switzerland with a mean annual number of hospitalisations of approximately 39,000. This registry continues to be analysed as part of a larger project [20]. Ethical approval was obtained from the Human Research Ethics Committee of the Canton of Vaud (2018-02196), and this permitted the partnering hospital's data warehouse to provide the appropriate dataset. Our study defined 'unplanned nursing home admission' as the impossibility for a formerly home-dwelling older inpatient to return there after hospital discharge, and this included any new admission to a nursing home following an acute care admission [2]. All the patients included in the study followed a home to hospital to long-term residential care facility pathway. Nursing homes do not expect their residents to return to independent living in the community. The extracted patient data contained sociodemographic characteristics, medical and surgical diagnoses, routinely assessed clinical data (such as gait, falls risk, hearing or pain) and the drugs prescribed. The medical and surgical diagnoses encoded diagnostic data using the WHO International Classification of Diseases, tenth version (ICD-10), and the Swiss Classification of Surgical Interventions (CHOP) [21]. The hospital dataset showed that discharged patients had been prescribed 2,370 different medicines. The Anatomical Therapeutic Chemical (ATC) classification system's 14 top-level codes were used to structure that dataset of prescribed medicines [22]. The extracted data, from multiple dataset sources, were transformed and synthesised using best practices [23]. Our dataset was composed of 14,705 hospital admissions from home settings between 2015 and 2018. Data were without missing values, and there were similar numbers of annual hospital admissions: 3,777, 3,534, 3,724 and 3,670, respectively.

Patient and public involvement

Patients were not involved in the development of the research questions, study design, outcome measures, or the conduct of the study.

Dataset customising for predictive analysis

For peer review only - http://bmjopen.bmj.com/site/about/guidelines.xhtml

Synthesising the extracted data

Since where patients had arrived from and where they were discharged to were two distinct variables, the dataset was recoded and customised to identify the number of older inpatients admitted straight from their home and then discharged to a nursing home, as presented in a previous paper [24]. Each subject's unique identifier was used to distinguish between different observations from 2015 to 2018 and to account for hospital readmissions. Cases involved 9,430 different older adults, with an average of 1.56 hospital stays per person. Sociodemographic and clinical data were considered independent variables and used to compute the predictive models [24]. Unplanned nursing home admission after discharge from our participating hospital between 2015 and 2018 was identified by the difference between the original abode (home) and the destination at discharge (a nursing home), and this was recoded as the dependent variable of interest.

Sociodemographic and Hospital Variables

The analysis included two sociodemographic control variables: age and sex. Age was considered a continuous variable; its progressive impact was conclusive in preliminary investigations and previous studies [25].

Health Variables

Numerous variables were used to describe older adults' health status at the end of their hospital stay. The modelling analysis included three of the six hierarchical clusters preliminarily computed as being variables significantly associated with more unplanned nursing home admissions in the descriptive analysis: mobility, dependency in the ADL and cognitive status [24]. Cognitive status was measured at an ordinal level using five categorical variables (perception–alertness, orientation, attention, decision-making process and ability to learn). Finally, the year of hospitalisation was introduced as a control variable, based on the fact that hospital admissions occurring earlier in the four-year study were associated with a higher probability of unplanned nursing home admission [24].

Drugs

The WHO ATC Classification System [22] was used to select frequently prescribed drugs at discharge as independent variables for the predictive model. The selection of drug class interactions was based on a literature review and expert opinions [26]. A cut-off point of at least 30 subjects per drug category prescribed was necessary to have a critical mass of data for computing robust statistics. The number of drugs prescribed at hospital discharge was considered continuous.

For peer review only - http://bmjopen.bmj.com/site/about/guidelines.xhtml

Data analysis strategy

Data were extracted into a Microsoft Excel spreadsheet (Microsoft, Redmond, Washington, United States) and subsequently imported into SPSS software, version 26.0 (IBM Corp, Armonk, New York, United States). Associations with unplanned nursing home admission were examined based on previous studies: patient age and sex, hospital length of stay, the principal and secondary ICD-10 diagnoses, surgical interventions (CHOP) and prescribed drugs. No causality analyses were possible because data analysis was retrospective and based on routine data: there was no way of knowing medication regimens or functional status before hospitalisation and how these might be associated with unplanned nursing home admission. A multiple bivariate logistic regression analysis was conducted using cross-tabulations to investigate whether the sociodemographic, health and drugs data (more than one independent variable) significantly predicted unplanned nursing home admission from 2015 to 2018 (our single dichotomous outcome). In a second stage, a series of generalised estimating equations (GEE or population-averaged logistic regression models) were computed to predict how sets of predictors influenced the probability of unplanned nursing home admission. The variables included were derived from the significant associations between sociodemographic characteristics, clinical and medical conditions and unplanned nursing home admission (Table 1). This baseline model was completed using the drugs prescribed to older inpatients who underwent unplanned nursing home admission. Lastly, based on our literature review, known drug–drug interactions between different ATC drug classes were added to the baseline model. The model estimated each predictor's impact, other things being equal, by estimating its net impact controlling for confounding factors (adjusted odds ratios). This GEE model is generally considered very robust and efficient at dealing with panel or correlated data because it makes few explicit assumptions and is less vulnerable to misspecification [27]. A GEE model predicts for the entire population and not a specific individual. Since the data are based on a whole population, not a sample, the odds ratios' confidence intervals and statistical tests were used to indicate the robustness of relationships since they normally only make sense for statistical inference.

Results

Population description

Fifty-five per cent of the population sample were men, and the total sample's mean age was 78.16 years old (SD = 7.65). Mean hospital length of stay was 8.63 days (SD = 7.58) [24].

At discharge, 36.8% (n = 7,880) of subjects presented with impaired mobility, with 12.6% (n = 2,574) impaired in their ADL and 10.2% (n = 2,083) having an impaired cognitive status. The mean number of

For peer review only - http://bmjopen.bmj.com/site/about/guidelines.xhtml

ICD-10 diseases per subject was 4.59 (SD = 0.91), and each older adult's number of ICD-10 diseases was entered into the model as a proxy for multimorbidity. The mean number of surgical interventions performed (based on Switzerland's CHOP surgery coding system) [21] was 1.80 per hospitalisation (SD = 1.77). The most prevalent medical diagnoses among older inpatients were circulatory (23.4%; n = 4,788), infectious (2.7%; n = 559) and respiratory diseases (10.3%; n = 2,111), as well as traumatic injuries (11.7%; n = 2,385) and tumours (10.0%; n = 2,041).

The mean number of drugs prescribed at hospital discharge was 9.07 (SD = 3.32). Supplementary Table 1 presents descriptive statistics of the drugs prescribed at discharge.

Associations between unplanned nursing home admission, sociodemographic characteristics, and the prevalence of clinical and medical conditions

We found a prevalence of older adults discharged to unplanned nursing home admission of 6.1% (n = 897/N = 14,705) over the whole time period, with a slight decrease in prevalence going forward [7.3% (n = 276) in 2015 to 5.9% (n = 217) in 2018]. Bivariate associations showed that men had a lower prevalence of unplanned nursing home admission than women [4.0% (n = 328) vs 8.8% (n = 575)], as did 65–69-year-old subjects (2.2%; n = 49) compared with those 70–79 years old, 80–89 years old, and especially the oldest group, aged 90 or more [3.2% (n = 192), 8.3% (n = 437) and 19.7% (n = 225), respectively].

Being jointly affected by several diseases increased the prevalence of unplanned nursing home admission, from 1.8% (n = 5) for older adults with a single disease (ICD-10) to 6.8% (n = 797) for those with five diseases. Furthermore, the number of surgical interventions was negatively associated with the prevalence of unplanned nursing home admission. Patients who had not undergone surgery showed a higher probability of unplanned nursing home admission (7.8%; n = 379) than those who had undergone several interventions [3.5% (n = 39) for four interventions, 4.2% (n = 84) for five interventions] (Table 1). The number of drugs prescribed at hospital discharge showed a positive linear relationship with unplanned nursing home admission (gamma = 0.368) (Figure 1).

[Insert Table 1]

[Insert Figure 1]

Associations between unplanned nursing home admission and drugs

Bivariate associations showed that drugs were also related to unplanned nursing home admission (Table 2). On average, older adults whose discharge to a nursing home was unplanned had more prescribed drugs than those returning home [10.9 (SD = 3.9) drugs vs 8.9 (SD = 3.2)]. Psycholeptics

For peer review only - http://bmjopen.bmj.com/site/about/guidelines.xhtml

(antipsychotics, anxiolytics, hypnotics and sedatives) and psychoanaleptic drugs (antidepressants, psychostimulants, nootropics and anti-dementia drugs), antiemetics and antinauseants, anti-Parkinson's disease drugs, and drugs treating constipation and the sensory organs were significantly associated with unplanned nursing home admission. On the contrary, patients taking lipid-modifying agents were less prone to unplanned nursing home admission.

[Insert Table 2]

Multivariate baseline model

A baseline, GEE logistic regression model, including sociodemographic information, clinical data, and diseases, was computed to predict unplanned nursing home admission among discharged polymedicated older adult patients (Figure 2 and Supplementary Table 2); prescribed drugs at hospital discharge were not included. If the 95% confidence interval (CI) does not overlap the null value (e.g. OR = 1), then the higher the odds ratio, the more the variable contributes to unplanned nursing home admission. Men had a lower probability of unplanned nursing home admission than women (OR = 0.62; 95% CI 0.52 to 0.73). Patients' probability of unplanned nursing home admission increased with age (OR = 1.07 for each additional year of age; 95% CI 1.05 to 1.08). Impaired mobility, dependency in the ADL and cognitive impairment revealed their substantial impacts on unplanned nursing home admission (OR = 3.22; 95% CI 2.67 to 3.87; OR = 4.62; 95% CI 3.76 to 5.67; and OR = 3.75; 95% CI 3.06 to 4.59, respectively). Circulatory and infectious diseases were related to lower probabilities of unplanned nursing home admission (OR = 0.78; 95% CI 0.63 to 0.98, and OR = 0.38; 95% CI 0.20 to 0.70, respectively), whereas traumatic injuries were related to higher probabilities (OR = 1.58; 95% CI 1.25 to 2.01). The number of ICD-10 diagnoses alone had no significant impact on the odds of unplanned nursing home admission (OR = 1.11; 95% CI 0.98 to 1.24), in contrast to the number of surgical interventions undergone (CHOP), which was a protective factor against unplanned hospitalisation (OR = 0.95; 95% CI 0.90 to 0.99). The year of hospital stay also had a significant impact, with more recent stays having lower probabilities of unplanned nursing home admission (OR = 0.88; 95% CI 0.82 to 0.94, per ensuing year).

[Insert Figure 2]

Prediction of unplanned nursing home admission and drug prescription

A higher number of prescribed drugs was associated with a higher probability of unplanned nursing home admission (OR = 1.17; 95% CI 1.15 to 1.19). Figure 3 and Supplementary Table 3 present the baseline GEE logistic regression model shown in Figure 2 completed with those drugs prescribed to older adults at discharge that had a significant statistical association ($p < 0.05$) with unplanned nursing

For peer review only - http://bmjopen.bmj.com/site/about/guidelines.xhtml

home admission. Drugs without a significant statistical association are not presented in Figure 3 for simplification purposes. Antiemetics and antinauseants (OR = 2.53; 95% CI 1.21 to 5.30 for each additional unit), digestives (OR = 1.78; 95% CI 1.09 to 2.90), psycholeptics (OR = 1.76; 95% CI 1.60 to 1.93), antiepileptics (OR = 1.49; 95% CI 1.25 to 1.79) and anti-Parkinson's disease drugs (OR = 1.40; 95% CI 1.12 to 1.75) were strongly linked to unplanned nursing home admission after controlling for other parameters. On the contrary, taking lipid-metabolism-modifying agents was associated with lower probabilities of unplanned nursing home admission (OR = 0.73; 95% CI 0.60 to 0.90, for each extra drug from this class prescribed).

[Insert Figure 3]

Combined drug intake and probabilities of unplanned nursing home admission

To reduce collinearity and simplify the results, the combined intake of different ATC drug classes was recoded as a dichotomised variable for each drug pairing and added to the previous model [22]. Only the drugs and drug combinations prescribed to older adults at discharge that had significant associations ($p < 0.05$) with unplanned nursing home admission are presented. The combined intake of cardiac therapy and psychoanaleptic drugs was significantly associated with unplanned nursing home admission (OR = 1.87; 95% CI 1.11 to 3.16), as were psychoanaleptics and diabetes drugs combined (OR = 1.75; 95% CI 1.03 to 2.98), and psycholeptic drugs and vitamins combined (OR = 1.71; 95% CI 1.03 to 2.84). On the contrary, the combined intake of beta-blocking agents and antiepileptics strongly diminished the odds of unplanned nursing home admission (OR = 0.39; 95% CI 0.23 to 0.67).

We also investigated the risk of unplanned nursing home admission for combined drug intake within the same drug class. The combined intake of two or more antiemetic and antinauseants (OR = 2.65; 95% CI 1.26 to 5.58), psycholeptics (OR = 1.64; 95% CI 1.46 to 1.85), antiepileptics (OR = 1.55; 95% CI 1.23 to 1.96) or anti-Parkinson's disease drugs (OR = 1.44; 95% CI 1.13 to 1.83) were strongly associated with a higher probability of unplanned nursing home admission.

Supplementary Table 4 summarises the main findings from our predictive analysis.

Discussion

This population-based hospital registry study used longitudinal data to examine the unplanned nursing home admission of hospitalised polymedicated older inpatients, revealing a 6.1% prevalence rate over the four-year dataset, in agreement with previous work by Luppa *et al*. (men: 5.4%; women: 6.0%) and Goodwin *et al*. (5.5%) [7, 28]. The slight decrease in prevalence over the four years of the study may be explained by improvements in the regional home-care services' contribution to maintaining older adults at home, but also to planned nursing home admissions without the requirement for

For peer review only - http://bmjopen.bmj.com/site/about/guidelines.xhtml

intermediate hospitalisation [29]. Furthermore, the number of places in the region's nursing homes increased in that period [30], allowing people for whom care at home became impossible to be admitted to a nursing home more promptly.

Our predictive analysis revealed that the group of the oldest adults, presenting functional mobility impairments, dependency in the ADL and cognitive impairment, was also at a high risk of unplanned nursing home admission, which is consistent with previous retrospective and prospective studies [31, 32]. Very old inpatients (≥ 90 years old) had an almost tenfold higher risk of unplanned nursing home admission than those aged 65–69. This was expected and matched with previous research [33], bearing in mind that the very oldest group presented with a high prevalence of multimorbidity and advanced functional and cognitive impairments. Unexpectedly, regardless of age, our results showed that older women had a higher prevalence and probability of unplanned nursing home admission than men [34]. Yet, our data could not entirely explain this result. Previous publications have indicated that social and life-expectancy factors may play roles in the different rates of nursing home admission between older adult men and women [34].

Our findings highlighted that functional and cognitive impairments were strong risk factors for unplanned nursing home admission, which is in line with the studies by Luppa *et al*. and Goodwin *et al*. [28, 34]. Likewise, our results emphasised a high risk of unplanned nursing home admission among non-surgically treated and trauma patients. This could be explained by the relationship between orthopaedic guidelines on traumatic injuries among older adults that suggest avoiding surgery, for several medical reasons (number and severity of multimorbidities), and which may lead to increased functional impairment and unplanned nursing home admission, as suggested by Gardner *et al*. and Cutugno [35, 36].

As might be expected, older adults who underwent an unplanned nursing home admission had more prescribed drugs than those returning home. Our results were in line with the retrospective study by Lucchetti *et al*., which demonstrated a relationship between the prescription of cardiovascular, gastrointestinal, and metabolic drugs and unplanned nursing home admission [37].

Our findings indicated that patients prescribed more than one drug from the same class of drugs—from the classes of antiemetics and antinauseants, psycholeptics, antiepileptics, or anti-Parkinson's disease drugs—had a higher risk of discharge to a nursing home. Although this phenomenon is still under-investigated, our findings are not in line with the few existing studies in this area, which have presented no significant relationships between drug interactions and unplanned nursing home admission [38]. However, in hospital settings, a recent systematic review reported drug–drug interactions among 80% or more of older inpatients [39]. Since polymedicated older inpatients should

be considered as a population at a high risk of adverse outcomes, further studies should investigate how drug–drug interactions might predict the risks of nursing home admission.

Our findings undeniably mirrored existing evidence that chronic conditions and debilitating comorbidities are significant risk factors for unplanned nursing home admission [3, 7]. However, they also raised questions regarding hospitalisation's effects on the individual ageing process, which likely interact to produce a cascade of factors towards functional decline and dependency [1]. The adverse effects of hospitalisation begin immediately and progress rapidly [1]. Harrison *et al*. and Haaksma *et al*. described ways in which acute and exacerbated acute and chronic disorders, reinforced by existing undiagnosed geriatric syndromes (frailty, delirium, pressure sores, functional incontinence), contributed to hospitalised older patients being unable to return home and needing to be discharged to a nursing home [2, 16]. Previous studies suggested that silent geriatric syndromes such as frailty and functional decline, together with polypharmacy, are not only clinically characteristic of older adults but also potential predictors of being at risk of a further loss of independence and subsequent nursing home admission. Montes *et al*. pointed out the dramatic rise in numbers of frail, hospitalised older adults. This increase generates concerns about whether nursing homes—already suffering from long admission waiting lists of home-dwelling older adults—will be able to cope with older adults' complex care needs [40].

Although some of the predisposing predictors identified cannot be treated (i.e. sex, age), they may still contribute to an older adult's risk of being discharged to a nursing home and subsequently exacerbate their situation there. Given that hospitalisation introduces stressors that may increase the chances of unplanned nursing home admission [41, 42], using patients' electronic hospital data could help identify the high-risk older adults who would benefit from specific preventive interventions. Being able to rapidly identify inpatients at a high risk of unplanned nursing home admission may help professional caregivers to provide them with the appropriate community-health resources, such as community-based rehabilitation programmes. This would help older people to remain in their community for longer.

Study strengths and limitations

Although our population-based study's findings could be generalised to other regions of Switzerland, any interpretations should be made with caution. Nevertheless, our findings could provide information to help better define which integrated healthcare approaches could be implemented to attenuate the risk factors associated with unplanned nursing home admission following an acute hospital admission or readmission. The numerous predictors revealed in our study enabled us to conceptualise an overview of hospitalised older adults' health conditions before their unplanned

For peer review only - http://bmjopen.bmj.com/site/about/guidelines.xhtml

nursing home admission. As healthcare moves towards ever-more personalised medicine, this result could help create more refined, tailored, future interventions via 'risk profiles' defined using each older adult's personal predictors.

Our study had some limitations. The absence of data on patients' functional status before hospital admission meant that we could not assess changes to that status during hospitalisation, such as the influence of the development or deterioration of functional and cognitive impairment. We did not compute analysis on specific disorders such as neurodegenerative diseases like dementia and Parkinson's disease because this was beyond the scope of our study protocol. However, further analyses could confirm earlier studies showing that these diseases significantly affect a person's risk of nursing home admission after hospitalisation, with almost 90% of patients with dementia being admitted into a nursing home before dying [15, 16]. Although the study considered statistical associations between drugs and unplanned nursing home admission, it did not use clinically diagnosed drug–drug interactions. Lastly, our data were unable to identify hospitalisations that might have been triggered by limited care options at home or hospitalisations that were necessary while awaiting a place in a nursing home. These cases of planned nursing home admissions could not be distinguished from the unplanned nursing home admissions considered in the study. In addition, some patients may not have been transferred directly from hospital to nursing homes and may have had to stay in an intermediate structure while awaiting a place. These patients were not included in the study due to the unavailability of this information in the database.

Conclusion

The sociodemographic characteristics of hospitalised older inpatients, together with their clinical and medical conditions and their prescribed drugs, can provide us with a significant set of risk factors for unplanned nursing home admission, sustaining our stated hypotheses. Identifying these risk factors for unplanned nursing home admission could be of great assistance in developing predictive tools and tailored intervention programmes aimed at reducing the number of older adults placed in nursing homes. Our results showed that the patient-related risk factors leading to nursing home admission were based on declines in physical and cognitive function. Treatment with single drugs and combinations of drugs were also associated with unplanned nursing home admission, indicating that multiple chronic health conditions are important risk factors of a non-return home. Our findings may help to identify those older inpatients at the greatest risk of unplanned nursing home admission, enabling their care to be optimised by counterbalancing those risk factors. Further research is required across large samples of older inpatients to investigate whether tailored interventions at early stages

For peer review only - http://bmjopen.bmj.com/site/about/guidelines.xhtml

in chronic diseases could delay physical and cognitive dysfunction and reduce unplanned nursing home admissions among this growing segment of the population.

Acknowledgement

The authors thank the partner hospital, including the hospital's data warehouse, for its valuable contributions.

Ethics approval and patient consent

Ethical approval was obtained from the Human Research Ethics Committee of the Canton of Vaud (CER-VD, 2018-02196), thus permitting our partner hospital's data warehouse to provide the appropriate dataset. Given the retrospective data source, obtaining consent from the patients concerned was impossible or posed disproportionate difficulties. The present study respects the legal requirements for research projects involving data re-use without consent, as set out in Art. 34 from the Swiss Human Research Act (HTA).

Conflict of Interest Statement

The authors have no conflicts of interest to declare.

Funding Sources

This work was supported by Swiss National Science Foundation grant number 407440_183434/1.

Author Contributions

BW, FP, and HV had the original idea. BW, MdRC, MMM and HV provided conceptual and methodological expertise to the study design and BW, FP, CMM, AvG, and HV to data analysis and interpretation. BW, FP, and HV were major contributors to writing the manuscript. All authors read, edited, and approved the final manuscript.

Data Availability Statement

As part of the Data Use Agreement, authors are not allowed to provide raw data. Upon a reasonable request, the corresponding author will provide statistical programming code used to generate results.

Figure Legends

Figure 1. Relationship between unplanned nursing home admission and number of prescribed drugs at discharge.

For peer review only - http://bmjopen.bmj.com/site/about/guidelines.xhtml

Figure 2. Baseline GEE logistic regression model with unplanned nursing home admission as the dependent variable associated with sociodemographic, hospitalisation, and independent clinical and medical variables (N = 14,705 observations for 9,430 different subjects).

Figure 3. The GEE logistic regression model of the drugs prescribed to older adults at discharge with significant predictive values (odds ratios) for unplanned nursing home admission (N = 14,705 observations for 9,430 different subjects).

441

For peer review only

For peer review only - http://bmjopen.bmj.com/site/about/guidelines.xhtml

References

1. Tasheva P, Vollenweider P, Kraege V, Roulet G, Lamy O, Marques-Vidal P, et al. Association Between Physical Activity Levels in the Hospital Setting and Hospital-Acquired Functional Decline in Elderly Patients. JAMA Netw Open. 2020;3(1):e1920185.
2. Harrison JK, Garrido AG, Rhynas SJ, Logan G, MacLullich AM, MacArthur J, et al. New institutionalisation following acute hospital admission: a retrospective cohort study. Age Ageing. 2017;46(2):238-44.
3. Gaugler JE, Duval S, Anderson KA, Kane RL. Predicting nursing home admission in the U.S: a meta-analysis. BMC Geriatr. 2007;7:13.
4. Oseran AS, Lage DE, Jernigan MC, Metlay JP, Shah SJ. A "Hospital-Day-1" Model to Predict the Risk of Discharge to a Skilled Nursing Facility. J Am Med Dir Assoc. 2019;20(6):689-95 e5.
5. Huang H-H, Chang JC-Y, Tseng C-C, Yang Y-J, Fan J-S, Chen Y-C, et al. Comprehensive geriatric assessment in the emergency department for the prediction of readmission among older patients: A 3-month follow-up study. Archives of Gerontology and Geriatrics. 2021;92:104255.
6. Middleton A, Li S, Kuo YF, Ottenbacher KJ, Goodwin JS. New Institutionalization in Long-Term Care After Hospital Discharge to Skilled Nursing Facility. J Am Geriatr Soc. 2018;66(1):56-63.
7. Luppa M, Luck T, Weyerer S, Konig HH, Brahler E, Riedel-Heller SG. Prediction of institutionalization in the elderly. A systematic review. Age Ageing. 2010;39(1):31-8.
8. Bellelli G, Magnifico F, Trabucchi M. Outcomes at 12 months in a population of elderly patients discharged from a rehabilitation unit. J Am Med Dir Assoc. 2008;9(1):55-64.
9. Fogg C, Griffiths P, Meredith P, Bridges J. Hospital outcomes of older people with cognitive impairment: An integrative review. Int J Geriatr Psychiatry. 2018;33(9):1177-97.
10. Landefeld CS, Palmer RM, Kresevic DM, Fortinsky RH, Kowal J. A randomized trial of care in a hospital medical unit especially designed to improve the functional outcomes of acutely ill older patients. N Engl J Med. 1995;332(20):1338-44.
11. Ferrucci L, Guralnik JM, Pahor M, Corti MC, Havlik RJ. Hospital diagnoses, Medicare charges, and nursing home admissions in the year when older persons become severely disabled. JAMA. 1997;277(9):728-34.
12. Ferrucci L, Bandinelli S, Benvenuti E, Di Iorio A, Macchi C, Harris TB, et al. Subsystems contributing to the decline in ability to walk: bridging the gap between epidemiology and geriatric practice in the InCHIANTI study. J Am Geriatr Soc. 2000;48(12):1618-25.
13. Koirala B, Hansen BR, Hosie A, Budhathoki C, Seal S, Beaman A, et al. Delirium point prevalence studies in inpatient settings: A systematic review and meta-analysis. J Clin Nurs. 2020;n/a(n/a).
14. Welch C, McCluskey L, Wilson D, Chapman GE, Jackson TA, Treml J, et al. Delirium is prevalent in older hospital inpatients and associated with adverse outcomes: results of a prospective multi-centre study on World Delirium Awareness Day. BMC Medicine. 2019;17(1):229.
15. Zekry D, Herrmann FR, Grandjean R, Vitale AM, De Pinho MF, Michel JP, et al. Does dementia predict adverse hospitalization outcomes? A prospective study in aged inpatients. Int J Geriatr Psychiatry. 2009;24(3):283-91.
16. Haaksma ML, Rizzuto D, Ramakers I, Garcia-Ptacek S, Marengoni A, van der Flier WM, et al. The Impact of Frailty and Comorbidity on Institutionalization and Mortality in Persons With Dementia: A Prospective Cohort Study. J Am Med Dir Assoc. 2019;20(2):165-70 e2.
17. Fried TR, O'Leary J, Towle V, Goldstein MK, Trentalange M, Martin DK. Health outcomes associated with polypharmacy in community-dwelling older adults: a systematic review. Journal of the American Geriatrics Society. 2014;62(12):2261-72.
18. Wimmer BC, Dent E, Visvanathan R, Wiese MD, Johnell K, Chapman I, et al. Polypharmacy and medication regimen complexity as factors associated with hospital discharge destination among older people: a prospective cohort study. Drugs Aging. 2014;31(8):623-30.

For peer review only - http://bmjopen.bmj.com/site/about/guidelines.xhtml

19. Koppitz AL, Dreizler J, Altherr J, Bosshard G, Naef R, Imhof L. Relocation experiences with unplanned admission to a nursing home: a qualitative study. Int Psychogeriatr. 2017;29(3):517-27.

20. Pereira F, Roux P, Santiago-Delefosse M, von Gunten A, Wernli B, Martins MM, et al. Optimising medication management for polymedicated home-dwelling older adults with multiple chronic conditions: a mixed-methods study protocol. BMJ Open. 2019;9(10):e030030.

21. Swiss Federal Statistical Office. Swiss Classification of Surgical Procedures (CHOP). 2017. Report No.: 9783303142851.

22. WHO. The Anatomical Therapeutic Chemical Classification System with Defined Daily Doses (ATC/DDD) Geneva: World Health Organization; 2014 [24 March 2020]. Available from: http://www.who.int/classifications/atcddd/en/.

23. Olsen J. Register-based research: some methodological considerations. Scand J Public Health. 2011;39(3):225-9.

24. Taushanov Z, Verloo H, Wernli B, Giovanni SD, Gunten Av, Pereira F. Transforming a Patient Registry Into a Customized Data Set for the Advanced Statistical Analysis of Health Risk Factors and for Medication-Related Hospitalization Research: Retrospective Hospital Patient Registry Study. JMIR Medical Informatics. 2021;9(5).

25. Andrade C. Age as a variable: Continuous or categorical? Indian J Psychiatry. 2017;59(4):524-5.

26. Haider SI, Johnell K, Thorslund M, Fastbom J. Trends in polypharmacy and potential drug-drug interactions across educational groups in elderly patients in Sweden for the period 1992 - 2002. Int J Clin Pharmacol Ther. 2007;45(12):643-53.

27. Yang HZ, Qiao XG, Das S, Paul MC. Thermal regenerated grating operation at temperatures up to 1400 degrees C using new class of multimaterial glass-based photosensitive fiber. Opt Lett. 2014;39(22):6438-41.

28. Goodwin JS, Howrey B, Zhang DD, Kuo YF. Risk of continued institutionalization after hospitalization in older adults. J Gerontol A Biol Sci Med Sci. 2011;66(12):1321-7.

29. Gillès De Pélichy E, Verloo H, Gasser J, Monod S. Intérêts et nécessité du renfort des soins à domicile dans le cadre des situations de crise psychique-Le résultat d'une politique de santé publique forte. Rev Med Suisse. 2020:1741-4.

30. Observatoire valaisan de la santé. Statistique cantonale des établissements médico-sociaux (EMS). OVS; 2020.

31. Salminen M, Laine J, Vahlberg T, Viikari P, Wuorela M, Viitanen M, et al. Factors associated with institutionalization among home-dwelling patients of Urgent Geriatric Outpatient Clinic: a 3-year follow-up study. Eur Geriatr Med. 2020;11(5):745-51.

32. Yoo JW, Nakagawa S, Kim S. Delirium and transition to a nursing home of hospitalized older adults: a controlled trial of assessing the interdisciplinary team-based "geriatric" care and care coordination by non-geriatrics specialist physicians. Geriatr Gerontol Int. 2013;13(2):342-50.

33. Del Ducal GF, da Silva SG, ThuméI E, Santos IS, Hallal PC. Predictive factors for institutionalization of the elderly: a case-control study. Rev Saúde Pública 2012;46(1).

34. Luppa M, Luck T, Weyerer S, Konig HH, Riedel-Heller SG. Gender differences in predictors of nursing home placement in the elderly: a systematic review. Int Psychogeriatr. 2009;21(6):1015-25.

35. Gardner RL, Harris F, Vittinghoff E, Cummings SR. The risk of fracture following hospitalization in older women and men. Arch Intern Med. 2008;168(15):1671-7.

36. Cutugno CL. The 'Graying' of Trauma Care: Addressing Traumatic Injury in Older Adults. American Journal of Nursing. 2011;111(11):40-8.

37. Lucchetti G, Granero AL, Pires SL, Gorzoni ML. Fatores associados à polifarmácia em idosos institucionalizados. Revista Brasileira de Geriatria e Gerontologia. 2010;13:51-8.

38. Castilho ECD, Reis A, Borges T, Siqueira L, Miasso A. Potential drug–drug interactions and polypharmacy in institutionalized elderly patients in a public hospital in Brazil. Journal of psychiatric and mental health nursing. 2018;25(1):3-13.

For peer review only

For peer review only - http://bmjopen.bmj.com/site/about/guidelines.xhtml

39. de Oliveira LM, Diel JdAC, Nunes A, Dal Pizzol TdS. Prevalence of drug interactions in hospitalised elderly patients: a systematic review. European Journal of Hospital Pharmacy. 2021;28(1):4-9.
40. Montes Reula L, Cañete Lairla M, Navarro López J, Pelegrín Valero C, Galindo Ortiz de Landázuri J, Marijuán Fernández P, et al. Predominant factors of institutionalization in the elderly: a comparative study between home nursing and community dwelling2020.
41. Carvalho TC, Valle APd, Jacinto AF, Mayoral VFdS, Boas PJFV. Impact of hospitalization on the functional capacity of the elderly: A cohort study. Revista Brasileira de Geriatria e Gerontologia. 2018;21(2):134-42.
42. Covinsky KE, Pierluissi E, Johnston CB. Hospitalization-associated disability:"She was probably able to ambulate, but I'm not sure". Jama. 2011;306(16):1782-93.

For peer review only

Table 1. Prevalence of unplanned nursing home admissions with regards to associations with sociodemographic characteristics and clinical and medical conditions among polymedicated hospitalised older adults (N = 14,705)

**Variables**	**Unplanned nursing home admission, n (%)**	***p*-value**
Overall sample of older adults (n = 14,705)	897 (6.1)	
Sex		$p < 0.001$
Female/Male	575 (8.8)/328 (4.0)	
Age in years		$p < 0.001$
65–69 years	49 (2.2)	
70–79 years	192 (3.2)	
80–89 years	437 (8.3)	
90 years or more	225 (19.7)	
Mobility		$p < 0.001$
Full ability (0) / impairment (1)	214 (2.0)/689 (16.7)	
Dependence in the activities of daily living		$p < 0.001$
Full ability (0)/impairment (1)	472 (3.4)/431 (44.8)	
Mental status		$p < 0.001$
Full ability (0)/impairment (1)	531 (3.8)/ 372 (41.3)	
ICD-10 principal diagnosis: circulatory problems		$p < 0.001$
No (0)/Yes (1)	752 (6.7)/ 151 (4.3)	
ICD-10 principal diagnosis: infection		$p < 0.01$
No (0)/Yes (1)	892 (6.2)/ 11 (2.7)	
ICD-10 principal diagnosis: respiratory problems		Ns
No (0)/Yes (1)	797 (6.1)/106 (6.8)	
ICD-10 principal diagnosis: traumatic injuries		$p < 0.001$
No (0)/Yes (1)	720 (5.3)/183 (14.9)	
ICD-10 principal diagnosis: tumor		$p < 0.01$
No (0)/Yes (1)	835 (6.4)/ 68 (4.3)	
Number of ICD-10 diseases		$p < 0.001$
1	5 (1.8)	
2	17 (2.9)	
3	37 (3.9)	
4	47 (3.9)	
5 or more	797 (6.8)	
Number of surgical interventions (CHOP)		$p < 0.001$
0	379 (7.8)	
1	187 (6.4)	
2	135 (5.8)	
3	79 (5.2)	
4	39 (3.5)	
5 or more	84 (4.2)	
Year of hospitalisation		$p < 0.01$
2015	276 (7.3)	
2016	216 (6.1)	

For peer review only

For peer review only - http://bmjopen.bmj.com/site/about/guidelines.xhtml

2017	194 (5.2)	
2018	217 (5.9)	

Note. Ns = non significant

Table 2. Prevalence of unplanned nursing home admission among polymedicated hospitalised older adults (N = 14,705) with regards to associations with different classes of prescribed drugs

**Drugs (ATC code)**	**Unplanned nursing home admission**		
	**No drugs in this class n (%)**	**Drugs in this class n (%)**	***p*-value**
First level, main anatomical group			
Blood and blood-forming organ drugs (B)	180 (5.4)	723 (6.4)	ns
Dermatologicals (D)	828 (5.8)	75 (14.1)	$p < 0.001$
Genito-urinary system and sex hormones (G)	737 (6.1)	6.3%	ns
Systemic hormonal preparations, excluding sex hormones and insulins (H)	737 (6.1)	6.5%	ns
Anti-infectives for systemic use (J)	736 (6.4)	167 (5.3)	$p < 0.05$
Antineoplastic and immunomodulating agents (L)	881 (6.3)	22 (3.5)	$p < 0.01$
Drugs for the musculoskeletal system (M)	815 (6.4)	88 (4.3)	$p < .001$
Antiparasitic products, insecticides, and repellents (P)	893 (6.2)	10 (4.0)	Ns
Respiratory system drugs (R)	771 (6.3)	132 (5.5)	Ns
Sensory organ drugs (S)	752 (5.5)	151 (13.4)	$p < 0.001$
Second level, therapeutic subgroup			
Stomatological preparations (A01)	899 (6.1)	4 (7.5)	ns
Drugs for acid-related disorders (A02)	384 (5.8)	519 (6.4)	ns
Drugs for functional gastrointestinal disorders (A03	805 (5.9)	98 (9.8)	$p < 0.001$
Antiemetics and antinauseants (A04)	884 (6.1)	19 (18.6)	$p < 0.001$
Bile and liver therapy drugs (A05)	900 (6.1)	3 (7.9)	ns
Drugs for constipation (A06)	605 (4.8)	298 (13.5)	$p < 0.001$
Antidiarrheals, intestinal anti-inflammatory/anti-infective agents (A07)	863 (6.0)	40 (9.4)	$p < 0.01$
Digestives, including enzymes (A09)	883 (6.1)	20 (8.4)	Ns
Diabetes drugs (A10)	804 (6.6)	99 (3.9)	$p < 0.001$
Vitamins (A11)	801 (6.2)	102 (5.9)	ns
Mineral supplements (A12)	513 (4.8)	390 (9.6)	$p < 0.001$
Other alimentary tract and metabolism products (A16)	901 (6.1)	2 (5.9)	ns
Cardiac therapy drugs (C01)	792 (6.1)	111 (6.3)	ns
Antihypertensives (C02)	888 (6.2)	15 (4.6)	ns
Diuretics (C03)	621 (5.5)	282 (8.1)	$p < 0.001$
Peripheral vasodilators (C04)	901 (6.1)	2 (4.2)	ns
Vasoprotectives (C05)	884 (6.1)	19 (7.2)	ns
Beta blocking agents (C07)	588 (7.2)	315 (4.8)	$p < 0.001$
Calcium channel blockers (C08)	762 (6.1)	141 (6.1)	ns
Agents acting on the renin-angiotensin system (C09)	472 (7.2)	431 (5.3)	$p < 0.001$
Lipid-modifying agents (C10)	720 (8.2)	183 (3.1)	$p < 0.001$
Anesthetics (N01)	898 (6.1)	5 (13.5)	ns
Analgesics (N02)	158 (3.6)	745 (7.2)	$p < 0.001$
Antiepileptics (N03)	753 (5.7)	150 (10.3)	$p < 0.001$
Drugs against Parkinson's disease (N04)	814 (5.7)	89 (18.1)	$p < 0.001$
Psycholeptics (N05)	201 (2.4)	702 (11.0)	$p < 0.001$

For peer review only

For peer review only - http://bmjopen.bmj.com/site/about/guidelines.xhtml

Psychoanaleptics (N06)	565 (4.8)	338 (11.9)	$p < 0.001$
Other nervous system drugs (N07)	881 (6.1)	22 (5.9)	ns

Note. Ns = non significant

For peer review only - http://bmjopen.bmj.com/site/about/guidelines.xhtml

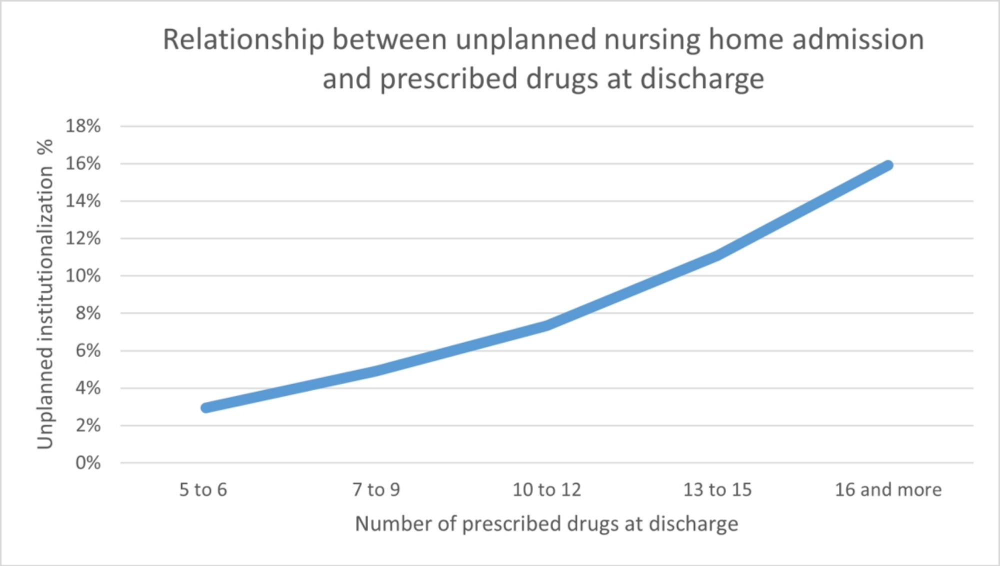

Relationship between unplanned nursing home admission and number of prescribed drugs at discharge.

152x86mm (300 x 300 DPI)

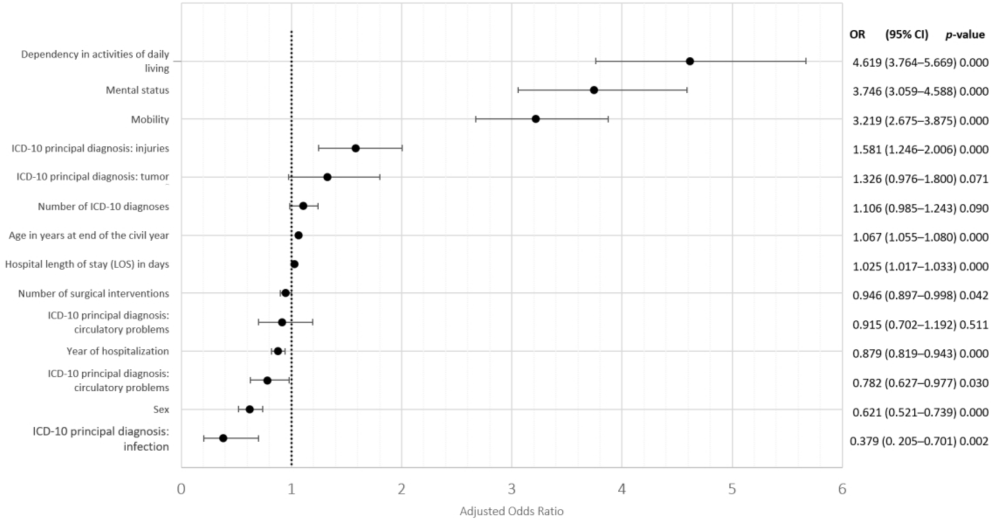

Baseline GEE logistic regression model with unplanned nursing home admission as the dependent variable associated with sociodemographic, hospitalisation, and independent clinical and medical variables (N = 14,705 observations for 9,430 different subjects).

299x157mm (144 x 144 DPI)

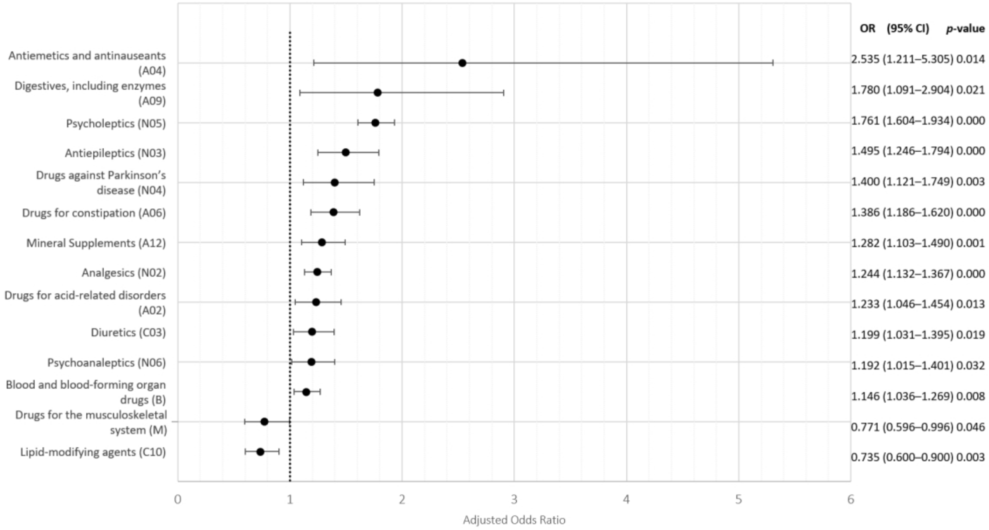

The GEE logistic regression model of the drugs prescribed to older adults at discharge with significant predictive values (odds ratios) for unplanned nursing home admission (N = 14,705 observations for 9,430 different subjects).

303x161mm (144 x 144 DPI)

Supplementary Table 1. Descriptive statistics of prescribed drugs at discharge based on the ATC among the polymedicated older inpatients (N = 14,705)

**Drugs by ATC, level 2**	**Number of drugs per patient**	
	**Min-Max**	**Mean (S.D.)**
First level, anatomical main group		
Blood and blood-forming organ drugs (B)	0-6	1.16 (0.86)
Dermatologicals (D)	0-3	0.04 (0.22)
Genito urinary system and sex hormones (G)	0-4	0.21 (0.47)
Systemic hormonal preparations, excl. sex hormones and insulins (H)	0-4	0.20 (0.46)
Anti-infective for systemic use (J)	0-4	0.23 (0.46)
Antineoplastic and immunomodulating agents (L)	0-5	0.05 (0.23)
Musculo skeletal system drugs (M)	0-3	0.15 (0.39)
Antiparasitic products, insecticides and repellents (P)	0-2	0.02 (0.13)
Respiratory system drugs (R)	0-7	0.27 (0.72)
Sensory organ drugs (S)	0-6	0.10 (0.40)
Second level, therapeutic subgroup		
Stomatological preparations (A01)	0-1	0.01 (0.06)
Drugs for acid related disorders (A02)	0-3	0.56 (0.52)
Drugs for functional gastrointestinal disorders (A03)	0-3	0.07 (0.28)
Antiemetics and antinauseants (A04)	0-1	0.01 (0.08)
Bille and liver therapy drugs (A05)	0-1	0.01 (0.05)
Drugs for constipation (A06)	0-4	0.17 (0.42)
Antidiarrheals, intestinal antiinflammatory/antiinfective agents (A07)	0-2	0.03 (0.18)
Digestives, incl. enzymes (A09)	0-2	0.02 (0.13)
Drugs used in diabetes (A10)	0-5	0.25 (0.63)
Vitamins (A11)	0-4	0.15 (0.44)
Mineral supplements (A12)	0-3	0.30 (0.51)
Other alimentary tract and metabolism products (A16)	0-1	0.01 (0.05)
Cardio-therapy drugs (C01)	0-4	0.14 (0.41)
Antihypertensives (C02)	0-2	0.02 (0.17)
Diuretics (C03)	0-3	0.28 (0.54)
Peripheral vasodilators (C04)	0-1	0.01 (0.06)
Vaso-protectives (C05)	0-3	0.02 (0.14)
Beta-blocking agents (C07)	0-2	0.45 (0.51)
Calcium channel blockers (C08)	0-2	0.16 (0.37)
Agents acting on the Renin-Angiotensin system (C09)	0-3	0.63 (0.62)
Lipid Modifying agents (C10)	0-3	0.41 (0.52)
Anesthetics (N01)	0-1	0.01 (0.05)
Analgesics (N02)	0-7	1.03 (0.91)
Antiepileptics (N03)	0-5	0.11 (0.36)
Anti-Parkinson drugs (N04)	0-5	0.04 (0.25)
Psycholeptics (N05)	0-7	0.57 (0.77)
Psychoanaleptics (N06)	0-3	0.21 (0.45)
Other nervous system drugs (N07)	0-3	0.03 (0.19)
Total number of drugs	5-32	9.07 (3.32)
N valid - listwise		14,70

For peer review only

Supplementary Table 2. Baseline, GEE logistic regression model with unplanned institutionalization as the dependent variable associated with sociodemographic, hospitalization, and independent clinical and medical variables (N = 14,705 observations for 9,430 different subjects).

**Variables**	**Odds Ratio**	***p* > z**	**95%Confidence Interval**
Sex [1]	0.62	< 0.000	0.52–0.74
Age in years	1.07	< 0.000	1.05–1.08
Hospital length of stay (LOS) in days	1.02	< 0.000	1.02–1.03
Mobility [2]	3.22	< 0.000	2.67–3.87
Dependency in the activities of daily living [2]	4.62	< 0.000	3.76–5.67
Mental status [2]	3.75	< 0.000	3.06–4.59
ICD-10 principal diagnosis: circulatory problems [3]	0.78	0.030	0.63–0.98
ICD-10 principal diagnosis: infection [3]	0.38	0.002	0.20–0.70
ICD-10 principal diagnosis: respiratory problems [3]	0.91	0.511	0.70–1.19
ICD-10 principal diagnosis: injuries [3]	1.58	< 0.000	1.25–2.01
ICD-10 principal diagnosis: tumor [3]	1.33	0.071	0.98–1.80
Number of ICD-10 diagnoses	1.11	0.090	0.98–1.24
Number of surgical interventions (CHOP)	0.95	0.042	0.90–0.99
Number of prescribed drugs	1.17	0.000	1.15–1.19
Year of hospitalization: 2015 to 2018	0.88	< 0.000	0.82–0.94

Note. 1: 0 = woman, 1 = man; 2: 0 = normal status, 1 = poor status; 3: 0 = no, 1 = yes

For peer review only

Supplementary Table 3. GEE logistic regression model of the drugs prescribed to older adults at discharge with significant predictive values (odds ratios) for unplanned institutionalization (N = 14,705 observations for 9,430 different subjects).

**Drugs**	**Odds Ratio**	***p* > z**	**95%Confidence Interval**
Antiemetics and antinauseants (A04)	2.53	0.014	1.21–5.30
Digestives, including enzymes (A09)	1.78	0.021	1.09–2.90
Psycholeptics (N05)	1.76	0.000	1.60–1.93
Antiepileptics (N03)	1.49	0.000	1.25–1.79
Anti-Parkinson drugs (N04)	1.40	0.003	1.12–1.75
Drugs for constipation (A06)	1.39	0.000	1.19–1.62
Mineral Supplements (A12)	1.28	0.001	1.10–1.49
Analgesics (N02)	1.24	0.000	1.13–1.37
Drugs for acid related disorders (A02)	1.23	0.013	1.05–1.45
Diuretics (C03)	1.20	0.019	1.03–1.39
Psychoanaleptics (N06)	1.19	0.032	1.01–1.40
Blood and blood-forming organ drugs (B)	1.15	0.008	1.04–1.27
Drugs for the musculoskeletal system (M)	0.77	0.046	0.60–0.99
Lipid-modifying agents (C10)	0.73	0.003	0.60–0.90

For peer review only

Supplementary Table 4. Factors associated with a higher and lower probability of unplanned nursing home admission among polymedicated hospitalised older adults (N = 14,705): summary of the predictive analysis.

**Risk factors for a higher probability of unplanned nursing home admission**
- Dependency in the activities of daily living (OR = 4.62, 95% CI: 3.76–5.67)
- Cognitive impairment (OR = 3.75, 95% CI: 3.06–4.59)
- Functional mobility impairment (OR = 3.22, 95% CI: 2.67–3.87)
- Antiemetics/antinauseants (OR = 2.53, 95% CI: 1.21–5.30)
- Digestives (OR = 1.78, 95% CI: 1.09–2.90)
- Psycholeptics (OR = 1.76, 95% CI: 1.60–1.93)
- Injuries (OR = 1.58, 95% CI: 1.25–2.01)
- Antiepileptics (OR = 1.49, 95% CI: 1.25–1.79)
- Anti-Parkinson's drugs (OR = 1.40, 95% CI: 1.12–1.75)
- Number of prescribed drugs (OR = 1.17, 95% CI: 1.15–1.19)
- Older age (OR = 1.07, 95% CI: 1.05–1.08)
Combined intake of:<br>- cardiac and psychoanaleptic drugs (OR = 1.87, 95% CI: 1.11–3.16)<br>- psychoanaleptic and diabetes drugs (OR = 1.75, 95% CI: 1.03–2.98)<br>- psycholeptic drugs and vitamins (OR = 1.71, 95% CI: 1.03–2.84)
Combined intake of two or more:<br>- antiemetics and antinauseants (OR = 2.65, 95% CI: 1.26–5.58)<br>- psycholeptics (OR = 1.64, 95% CI: 1.46–1.85)<br>- antiepileptics (OR = 1.55, 95% CI: 1.23–1.96)<br>- anti-Parkinson's drugs (OR = 1.44, 95% CI: 1.13–1.83)
**Protective factors for a lower probability of unplanned nursing home admission**
- Surgical interventions (OR = 0.95, 95% CI: 0.90–0.99)
- Circulatory diseases (OR = 0.78, 95% CI: 0.63–0.98)
- Lipid metabolism modifying agents (OR = 0.73, 95% CI: 0.60–0.90)
- Male sex (OR = 0.62; 95% CI: 0.52–0.73)
- Combined intake of beta-blocking agents and antiepileptics (OR = 0.39, 95% CI: 0.23–0.67)
- Infectious diseases (OR = 0.38, 95% CI: 0.20–0.70)

For peer review only

The RECORD statement – checklist of items, extended from the STROBE statement, that should be reported in observational studies using routinely collected health data.

	Item No.	STROBE items	Location in manuscript where items are reported	RECORD items	Location in manuscript where items are reported
**Title and abstract**					
	1	(a) Indicate the study's design with a commonly used term in the title or the abstract (b) Provide in the abstract an informative and balanced summary of what was done and what was found		RECORD 1.1: The type of data used should be specified in the title or abstract. When possible, the name of the databases used should be included.<br><br>RECORD 1.2: If applicable, the geographic region and timeframe within which the study took place should be reported in the title or abstract.<br><br>RECORD 1.3: If linkage between databases was conducted for the study, this should be clearly stated in the title or abstract.	Title<br>Abstract (line 34)<br><br>Line 6<br><br>Lines 35<br><br>Not applicable, only one hospital register
**Introduction**					
Background rationale	2	Explain the scientific background and rationale for the investigation being reported			Lines 75-148
Objectives	3	State specific objectives, including any prespecified hypotheses			Lines 145-148
**Methods**					
Study Design	4	Present key elements of study design early in the paper			Lines 151-153
Setting	5	Describe the setting, locations, and relevant dates, including periods of recruitment, exposure, follow-up, and data collection			Lines 155-177

For peer review only

Participants	6	*(a) Cohort study* - Give the eligibility criteria, and the sources and methods of selection of participants. Describe methods of follow-up<br>*Case-control study* - Give the eligibility criteria, and the sources and methods of case ascertainment and control selection. Give the rationale for the choice of cases and controls<br>*Cross-sectional study* - Give the eligibility criteria, and the sources and methods of selection of participants<br><br>*(b) Cohort study* - For matched studies, give matching criteria and number of exposed and unexposed<br>*Case-control study* - For matched studies, give matching criteria and the number of controls per case		RECORD 6.1: The methods of study population selection (such as codes or algorithms used to identify subjects) should be listed in detail. If this is not possible, an explanation should be provided.<br><br>RECORD 6.2: Any validation studies of the codes or algorithms used to select the population should be referenced. If validation was conducted for this study and not published elsewhere, detailed methods and results should be provided.<br><br>RECORD 6.3: If the study involved linkage of databases, consider use of a flow diagram or other graphical display to demonstrate the data linkage process, including the number of individuals with linked data at each stage.	Reported in a previous study: https://pubmed.ncbi.nlm.nih.gov/33973865/<br><br>Reported in a previous study: https://pubmed.ncbi.nlm.nih.gov/33973865/<br><br>Not applicable, only one hospital register
Variables	7	Clearly define all outcomes, exposures, predictors, potential confounders, and effect modifiers. Give diagnostic criteria, if applicable.		RECORD 7.1: A complete list of codes and algorithms used to classify exposures, outcomes, confounders, and effect modifiers should be provided. If these cannot be reported, an explanation should be provided.	Lines 181-222 and reported in a previous study: https://pubmed.ncbi.nlm.nih.gov/33973865/
Data sources/ measurement	8	For each variable of interest, give sources of data and details of methods of assessment (measurement).			Lines 181-222 and reported in a previous study:

For peer review only

		Describe comparability of assessment methods if there is more than one group			https://pubmed.ncbi.nlm.nih.gov/33973865/
Bias	9	Describe any efforts to address potential sources of bias			Lines 224-241 and reported in a previous study: https://pubmed.ncbi.nlm.nih.gov/33973865/
Study size	10	Explain how the study size was arrived at			Lines 155-177
Quantitative variables	11	Explain how quantitative variables were handled in the analyses. If applicable, describe which groupings were chosen, and why			Lines 224-241 and reported in a previous study: https://pubmed.ncbi.nlm.nih.gov/33973865/
Statistical methods	12	(a) Describe all statistical methods, including those used to control for confounding<br>(b) Describe any methods used to examine subgroups and interactions<br>(c) Explain how missing data were addressed<br>(d) *Cohort study* - If applicable, explain how loss to follow-up was addressed<br>*Case-control study* - If applicable, explain how matching of cases and controls was addressed<br>*Cross-sectional study* - If applicable, describe analytical			Lines 224-241

For peer review only

		methods taking account of sampling strategy<br>(e) Describe any sensitivity analyses			
Data access and cleaning methods		..		RECORD 12.1: Authors should describe the extent to which the investigators had access to the database population used to create the study population.<br><br>RECORD 12.2: Authors should provide information on the data cleaning methods used in the study.	Lines 224-241 and reported in a previous study: https://pubmed.ncbi.nlm.nih.gov/33973865/
Linkage		..		RECORD 12.3: State whether the study included person-level, institutional-level, or other data linkage across two or more databases. The methods of linkage and methods of linkage quality evaluation should be provided.	Reported in a previous study: https://pubmed.ncbi.nlm.nih.gov/33973865/
**Results**					
Participants	13	(a) Report the numbers of individuals at each stage of the study (*e.g.*, numbers potentially eligible, examined for eligibility, confirmed eligible, included in the study, completing follow-up, and analysed)<br>(b) Give reasons for non-participation at each stage.<br>(c) Consider use of a flow diagram		RECORD 13.1: Describe in detail the selection of the persons included in the study (*i.e.,* study population selection) including filtering based on data quality, data availability and linkage. The selection of included persons can be described in the text and/or by means of the study flow diagram.	Lines 170-177
Descriptive data	14	(a) Give characteristics of study participants (*e.g.*, demographic, clinical, social) and information on exposures and potential confounders			Lines 243-264

For peer review only

		(b) Indicate the number of participants with missing data for each variable of interest<br>(c) *Cohort study* - summarise follow-up time (*e.g.*, average and total amount)			
Outcome data	15	*Cohort study* - Report numbers of outcome events or summary measures over time<br>*Case-control study* - Report numbers in each exposure category, or summary measures of exposure<br>*Cross-sectional study* - Report numbers of outcome events or summary measures			Lines 243-264
Main results	16	(a) Give unadjusted estimates and, if applicable, confounder-adjusted estimates and their precision (e.g., 95% confidence interval). Make clear which confounders were adjusted for and why they were included<br>(b) Report category boundaries when continuous variables were categorized<br>(c) If relevant, consider translating estimates of relative risk into absolute risk for a meaningful time period			Lines 277-327
Other analyses	17	Report other analyses done—e.g., analyses of subgroups and interactions, and sensitivity analyses			Lines 268-280
**Discussion**					
Key results	18	Summarise key results with reference to study objectives			Lines 339-342

For peer review only

Limitations	19	Discuss limitations of the study, taking into account sources of potential bias or imprecision. Discuss both direction and magnitude of any potential bias		RECORD 19.1: Discuss the implications of using data that were not created or collected to answer the specific research question(s). Include discussion of misclassification bias, unmeasured confounding, missing data, and changing eligibility over time, as they pertain to the study being reported.	Lines 405-421
Interpretation	20	Give a cautious overall interpretation of results considering objectives, limitations, multiplicity of analyses, results from similar studies, and other relevant evidence			Lines 333-394
Generalisability	21	Discuss the generalisability (external validity) of the study results			Lines 396-400
**Other Information**					
Funding	22	Give the source of funding and the role of the funders for the present study and, if applicable, for the original study on which the present article is based			Line 451
Accessibility of protocol, raw data, and programming code		..		RECORD 22.1: Authors should provide information on how to access any supplemental information such as the study protocol, raw data, or programming code.	Lines 185, 188, 200-201<br><br>https://pubmed.ncbi.nlm.nih.gov/33973865/

*Reference: Benchimol EI, Smeeth L, Guttmann A, Harron K, Moher D, Petersen I, Sørensen HT, von Elm E, Langan SM, the RECORD Working Committee. The REporting of studies Conducted using Observational Routinely-collected health Data (RECORD) Statement. *PLoS Medicine* 2015; in press.

*Checklist is protected under Creative Commons Attribution (CC BY) license.

For peer review only - http://bmjopen.bmj.com/site/about/guidelines.xhtml

BMJ Open

Unplanned nursing home admission among discharged polymedicated older inpatients: a single-centre, registry-based study in Switzerland

Journal:	*BMJ Open*
Manuscript ID	bmjopen-2021-057444.R2
Article Type:	Original research
Date Submitted by the Author:	16-Feb-2022

Complete List of Authors:	Pereira, Filipa; University of Porto, Institute of Biomedical Sciences Abel Salazar; HES-SO Valais Wallis, School of Health Sciences<br>Verloo, Henk; HES-SO Valais Wallis, School of Health Sciences<br>von Gunten, Armin; Centre Hospitalier Universitaire Vaudois Département de Psychiatrie<br>del Río Carral, María; University of Lausanne, Institute of Psychology, Research Center for the Psychology of Health, Aging and Sports Examination<br>Meyer-Massetti, Carla; University of Bern, Institute for Primary Health Care (BIHAM)<br>Martins, Maria Manuela; Escola Superior de Enfermagem do Porto, Formação&gestão<br>Wernli, Boris; University of Lausanne, FORS, Swiss Centre of Expertise in the Social Sciences

**Primary Subject Heading**:	Geriatric medicine
Secondary Subject Heading:	Pharmacology and therapeutics, Health informatics
Keywords:	GERIATRIC MEDICINE, CLINICAL PHARMACOLOGY, Health informatics < BIOTECHNOLOGY & BIOINFORMATICS, EPIDEMIOLOGY

SCHOLARONE™
Manuscripts

I, the Submitting Author has the right to grant and does grant on behalf of all authors of the Work (as defined in the below author licence), an exclusive licence and/or a non-exclusive licence for contributions from authors who are: i) UK Crown employees; ii) where BMJ has agreed a CC-BY licence shall apply, and/or iii) in accordance with the terms applicable for US Federal Government officers or employees acting as part of their official duties; on a worldwide, perpetual, irrevocable, royalty-free basis to BMJ Publishing Group Ltd ("BMJ") its licensees and where the relevant Journal is co-owned by BMJ to the co-owners of the Journal, to publish the Work in this journal and any other BMJ products and to exploit all rights, as set out in our licence.

The Submitting Author accepts and understands that any supply made under these terms is made by BMJ to the Submitting Author unless you are acting as an employee on behalf of your employer or a postgraduate student of an affiliated institution which is paying any applicable article publishing charge ("APC") for Open Access articles. Where the Submitting Author wishes to make the Work available on an Open Access basis (and intends to pay the relevant APC), the terms of reuse of such Open Access shall be governed by a Creative Commons licence – details of these licences and which Creative Commons licence will apply to this Work are set out in our licence referred to above.

Other than as permitted in any relevant BMJ Author's Self Archiving Policies, I confirm this Work has not been accepted for publication elsewhere, is not being considered for publication elsewhere and does not duplicate material already published. I confirm all authors consent to publication of this Work and authorise the granting of this licence.

For peer review only

Unplanned nursing home admission among discharged polymedicated older inpatients: a single-centre, registry-based study in Switzerland

Filipa Pereira[1,2], Henk Verloo[1,3], Armin von Gunten[c], María del Río Carral[4], Carla Meyer-Massetti[5], Maria Manuela Martins[6], Boris Wernli[7]

[1] School of Health Sciences, HES-SO Valais/ Wallis, University of Applied Sciences and Arts Western Switzerland, Sion, Switzerland

[2] Institute of Biomedical Sciences Abel Salazar, University of Porto, Porto, Portugal

[3] Service of Old Age Psychiatry, Lausanne University Hospital, Lausanne, Switzerland

[4] Institute of Psychology, Research Center for the Psychology of Health, Aging and Sports Examination, University of Lausanne, Lausanne, Switzerland

[5] Institute for Primary Health Care (BIHAM), University of Bern, Bern, Switzerland

[6] Higher School of Nursing of Porto, Porto, Portugal

[7] FORS, Swiss Centre of Expertise in the Social Sciences, University of Lausanne, Switzerland

Corresponding Author:

Filipa Pereira

School of Health Sciences, HES-SO Valais / Wallis

University of Applied Sciences and Arts Western Switzerland

5 Chemin de l'Agasse, CH-1950 Sion, Switzerland

Institute of Biomedical Sciences Abel Salazar, University of Porto

228 Rua de Jorge Viterbo Ferreira, 4050-313 Porto, Portugal

Phone: +41 78 666 17 00

Email: filipa.pereira@hevs.ch

ORCID: https://orcid.org/0000-0001-9207-4856

For peer review only - http://bmjopen.bmj.com/site/about/guidelines.xhtml

Abstract

Objective: To investigate patient characteristics and the available health and drug data associated with unplanned nursing home admission following an acute hospital admission or readmission.

Design: A population-based hospital registry study.

Setting: A public hospital in southern Switzerland (Valais Hospital).

Participants: We explored a population-based longitudinal dataset of 14,705 hospital admissions from 2015–2018.

Outcome measures: Sociodemographic, health and drug data, and their interactions predicting the risk of unplanned nursing home admission.

Results: The mean prevalence of unplanned nursing home admission after hospital discharge was 6.1% (n = 903/N = 14,705). Our predictive analysis revealed that the oldest adults (OR = 1.07 for each additional year of age; 95%CI 1.05 to 1.08) presenting with impaired functional mobility (OR = 3.22; 95%CI 2.67 to 3.87), dependency in the activities of daily living (OR = 4.62; 95%CI 3.76 to 5.67), cognitive impairment (OR=3.75; 95%CI 3.06 to 4.59), and traumatic injuries (OR=1.58; 95%CI 1.25 to 2.01) had a higher probability of unplanned nursing home admission. The number of ICD-10 diagnoses had no significant impact on nursing home admissions, contrarily to the number of prescribed drugs (OR=1.17; 95%CI 1.15 to 1.19). Antiemetics/antinauseants (OR=2.53; 95%CI 1.21 to 5.30), digestives (OR=1.78; 95%CI 1.09 to 2.90), psycholeptics (OR=1.76; 95%CI 1.60 to 1.93), antiepileptics (OR=1.49; 95%CI 1.25 to 1.79) and anti-Parkinson's drugs (OR=1.40; 95%CI 1.12 to 1.75) were strongly linked to unplanned nursing home admission.

Conclusions: Numerous risk factors for unplanned nursing home admission were identified. To prevent the adverse health outcomes that precipitate acute hospitalisations and unplanned nursing home admissions, ambulatory-care providers should consider these risk factors in their care planning for older adults before they reach a state requiring hospitalisation.

Keywords: population-based sample; functional decline; hospital discharge; risk factors; nursing home

Strengths and limitations of this study:

For peer review only - http://bmjopen.bmj.com/site/about/guidelines.xhtml

- A hospital registry of 14,705 hospital admissions, involving 9,430 different polymedicated older adults admitted from their homes, was analysed to determine the risk of unplanned nursing home admission.
- Bivariate analyses were conducted on independent variables, and generalised estimating equations were computed to predict how sets of predictors influenced the adjusted probability of unplanned nursing home admission.
- Causality analysis was not feasible based on the nature of the routinely collected data.
- Although the study considered statistical associations between drugs and unplanned nursing home admission, it did not use clinically diagnosed drug–drug interactions.
- Our data were unable to identify hospitalisations that might have been triggered by limited home-care options or those that became necessary while older adults awaited a place in a nursing home.

Introduction

The hospitalisation of home-dwelling older adults, for any reason and even for a short admission, can lead to substantial functional decline [1, 2]. Both their health disorder itself and the hospital environment can foster such functional decline, increase the risk of future illness and irreversibly diminish their quality of life [1, 2]. Most hospitalised older adult inpatients wish to return home and continue their everyday life as before. However, these different factors may hinder this wish at discharge [3, 4]. The unmet patient needs related to functional decline and safety after returning home can lead to a higher risk of hospital and emergency department readmissions and thus to subsequent unplanned nursing home admission [5]. After hospitalisation, an unplanned nursing home admission can be a devastating and overwhelming experience for older adults and their relatives, and it increases overall healthcare system costs [6].

Whether planned or unplanned, nursing home admission commonly follows two paths: (i) within the community, directly from home, or (ii) from hospital, directly transitioning from hospital discharge [2]. In the community, transitions to nursing homes are generally the result of thoughtful decisions made by home-dwelling older adults, their families, and health- and social-care providers based on the evolution of the person's long-term health and functional state or on an acute decline and corresponding increase in care needs that cannot be met at home. Recent findings have suggested that the predictors of nursing home admission are mainly based on underlying cognitive and functional impairments combined with a lack of support and assistance in daily living at home [7].

For peer review only - http://bmjopen.bmj.com/site/about/guidelines.xhtml

The causes of unplanned nursing home admission directly after acute hospital discharge are heterogeneous. There are several reasons why older adults may require long-term care—that cannot be provided in a community setting—following acute hospital admission, e.g. a new medical problem or the worsening of existing chronic disease(s) entailing dependency and requiring complex forms of care. Furthermore, there may be a breakdown of family circumstances and/or lack of social support.

Bellelli *et al*. showed that advanced age (OR = 4.8; 95% CI 2.6 to 8.9, $p < 0.001$), cognitive impairment (OR = 2.3; 95% CI 1.4 to 3.9, $p < 0.001$) and poor functional status (OR = 10.2; 95% CI 4.7 to 22.5, $p < 001$) at discharge from a rehabilitation unit were the main predictors of subsequent nursing home admission [8]. The integrative review by Fogg *et al*. found a similar result for cognitive impairment (OR = 2.14; 95% CI 1.24 to 3.70, $p < 0.001$) [9]. A randomised controlled trial by Landefeld *et al*. found that older inpatients in an acute care medical unit with a decline in their ability to perform one or more of the basic activities of daily living (ADL) were more often discharged to a nursing home than those with less functional decline (22% and 14 %, respectively; $p < 0.01$) [10]. Ferrucci *et al*. identified stroke, cancer, congestive heart failure, pneumonia, coronary heart disease and hip fractures as the leading medical precipitators of functional decline and nursing home admission [11]. Older adult inpatients are frequently subject to iatrogenic events during hospitalisation, including adverse drug reactions, nosocomial infections, and the consequences of falls, fractures, and using chemical or physical restraints [12]. Such events can lengthen hospitalisation, produce cognitive changes and lessen the ability to perform the ADL, all potentially leading to unplanned nursing home admission [12]. Indeed, hospitalisation causes an increased risk of the onset of acute cognitive decline in the form of delirium, with a prevalence of up to 60% on some surgical wards [13], often leading to unplanned nursing home admission [14]. Dementia, Parkinson’s disease and its associated risk of falls, and behavioural changes are common reasons for deciding to transfer inpatients from hospital to long-term care [15, 16].

Polypharmacy has been associated with adverse health outcomes among home-dwelling older adults [17]. Some prospective studies with small samples have established relationships between drug treatments during acute hospitalisation and unplanned nursing home admission [18]. Cardiovascular drugs (particularly vasodilators, diuretics and anticoagulants), drugs against diabetes, steroids, non-steroidal anti-inflammatory drugs, opiates, antibiotics, anticholinergics and benzodiazepines have all been associated with unplanned nursing home admission [18].

To the best of our knowledge, and despite more frequent post-discharge nursing home admissions in Switzerland than in other countries, there is scarce research exploring how unplanned admissions to nursing homes are related to prior hospitalisation [19]. The present study aimed to investigate the

For peer review only - http://bmjopen.bmj.com/site/about/guidelines.xhtml

associations between polymedicated older inpatients' sociodemographic and clinical characteristics, drug data and their interactions, and their unplanned nursing home admission following an acute care hospital stay.

For peer review only

For peer review only - http://bmjopen.bmj.com/site/about/guidelines.xhtml

Methods

Study design

The present population-based hospital registry study was conducted with close regard to the REporting of studies Conducted using Observational Routinely collected health Data (RECORD) statement.

Population and data collection

Our four-year, longitudinal, population-based hospital registry of electronic health records included polymedicated (five and more drugs prescribed) home-dwelling older adults admitted and readmitted to the Valais hospital, a multisite public teaching hospital (1,074 beds) in southern Switzerland with a mean annual number of hospitalisations of approximately 39,000. This registry continues to be analysed as part of a larger project [20]. Ethical approval was obtained from the Human Research Ethics Committee of the Canton of Vaud (2018-02196), and this permitted the partnering hospital's data warehouse to provide the appropriate dataset. Our study defined 'unplanned nursing home admission' as the impossibility for a formerly home-dwelling older adult inpatient to return there after hospital discharge, and this included any new admission to a nursing home following an acute care admission [2]. All the patients included in the study followed a home to hospital to nursing home pathway. Nursing homes do not expect their residents to return to independent living in the community. The extracted patient data contained sociodemographic characteristics, medical and surgical diagnoses, routinely assessed clinical data (such as gait, falls risk, hearing or pain) and the drugs prescribed. The medical and surgical diagnoses encoded diagnostic data using the WHO International Classification of Diseases, tenth version (ICD-10), and the Swiss Classification of Surgical Interventions (CHOP) [21]. The hospital dataset showed that discharged patients had been prescribed 2,370 different medicines. The Anatomical Therapeutic Chemical (ATC) classification system's 14 top-level codes were used to structure that dataset of prescribed medicines [22]. The extracted data, from multiple dataset sources, were transformed and synthesised using best practices [23]. Our dataset was composed of 14,705 hospital admissions from home settings between 2015 and 2018. Data were without missing values, and there were similar numbers of annual hospital admissions: 3,777, 3,534, 3,724 and 3,670, respectively.

Patient and public involvement

Patients were not involved in the development of the research questions, study design, outcome measures, or the conduct of the study.

Dataset customising for predictive analysis

For peer review only - http://bmjopen.bmj.com/site/about/guidelines.xhtml

Synthesising the extracted data

Since where patients had arrived from and where they were discharged to were two distinct variables, the dataset was recoded and customised to identify the number of older adult inpatients admitted straight from their homes and then discharged to a nursing home (n = 903) or returning to their homes (n = 13,802), as presented in a previous paper [24]. Therefore, older adults who died during hospitalisation (as assessed by the Valais Hospital's healthcare staff) were automatically excluded (n = 131). Each subject's unique identifier was used to distinguish between different observations from 2015 to 2018 and to account for hospital readmissions. Cases involved 9,430 different older adults, with an average of 1.56 hospital stays per person. Sociodemographic and clinical data were considered independent variables and used to compute the predictive models [24]. Unplanned nursing home admission after discharge from our participating hospital between 2015 and 2018 was identified by the difference between the original abode (home) and the destination at discharge (a nursing home or their own home), and this was used as the dependent variable of interest.

Sociodemographic and Hospital Variables

The analysis included two sociodemographic control variables: age and sex. Age was considered a continuous variable; its progressive impact was conclusive in preliminary investigations and previous studies [25].

Health Variables

Numerous variables were used to describe older adults' health status at the end of their hospital stay. The modelling analysis included three of the six hierarchical clusters preliminarily computed as being variables significantly associated with more unplanned nursing home admissions in the descriptive analysis: mobility, dependency in the ADL and cognitive status [24]. Cognitive status was measured at an ordinal level using five categorical variables (perception–alertness, orientation, attention, decision-making process and ability to learn). Finally, the year of hospitalisation was introduced as a control variable, based on the fact that hospital admissions occurring earlier in the four-year study were associated with a higher probability of unplanned nursing home admission [24].

Drugs

The WHO ATC Classification System [22] was used to select frequently prescribed drugs at discharge as independent variables for the predictive model. The selection of drug class interactions was based on a literature review and expert opinions [26]. A cut-off point of at least 30 subjects per drug category prescribed was necessary to have a critical mass of data for computing robust statistics. The number of drugs prescribed at hospital discharge was considered continuous.

For peer review only - http://bmjopen.bmj.com/site/about/guidelines.xhtml

Data analysis strategy

Data were extracted into a Microsoft Excel spreadsheet (Microsoft, Redmond, Washington, United States) and subsequently imported into SPSS software, version 26.0 (IBM Corp, Armonk, New York, United States). Associations with unplanned nursing home admission were examined based on previous studies: patient age and sex, hospital length of stay, the principal and secondary ICD-10 diagnoses, surgical interventions (CHOP) and prescribed drugs. No causality analyses were possible because data analysis was retrospective and based on routine data: there was no way of knowing medication regimens or functional status before hospitalisation and how these might be associated with unplanned nursing home admission. A series of unadjusted bivariate analyses using cross-tabulations were conducted to investigate whether the sociodemographic, health and drugs data (more than one independent variable) were statistically significantly associated with unplanned nursing home admission from 2015 to 2018 (our single dichotomous outcome). In a second stage, a series of generalised estimating equations (GEE or population-averaged logistic regression models) were computed to predict how sets of predictors influenced the probability of unplanned nursing home admission. The variables entered at the first stage were derived from the significant associations between sociodemographic characteristics, clinical and medical conditions and unplanned nursing home admission (Table 1). The multivariable analysis model included 52 Level 2 ATC drug classes, respecting the good practices for logistical regressions involving large population-based samples [27]. This adjusted baseline model was then completed by adding drugs that were found to be significantly associated with unplanned nursing home admissions in the previous analysis. Lastly, based on our literature review, known drug–drug interactions between different ATC drug classes were added to the baseline model. The model estimated each predictor's impact, other things being equal, by estimating its net impact controlling for confounding factors (adjusted odds ratios).. Since the data are based on a whole population, not a sample, the odds ratios' confidence intervals and statistical tests were used to indicate the robustness of relationships since they normally only make sense for statistical inference.

Results

Population description

Fifty-five per cent of the population sample were men, and the total sample's mean age was 78.16 years old (SD = 7.65). Mean hospital length of stay was 8.63 days (SD = 7.58). The mean number of drugs prescribed at hospital discharge was 9.07 (SD = 3.32), with means of 10.91 (SD = 3.89) drugs for

For peer review only - http://bmjopen.bmj.com/site/about/guidelines.xhtml

patients discharged to a nursing home versus 8.95 (SD = 3.24) for those discharged home. Supplementary Tables 1 and 2 present descriptive statistics of the older adult inpatients' health statuses and drugs prescribed at discharge.

Associations between unplanned nursing home admission, sociodemographic characteristics, and the prevalence of clinical and medical conditions

We found a prevalence of older adults discharged to unplanned nursing home admission of 6.1% (n = 903/N = 14,705) over the whole time period, with a slight decrease in prevalence going forward [7.3% (n = 276) in 2015 to 5.9% (n = 217) in 2018]. Bivariate associations showed that men had a lower prevalence of unplanned nursing home admission than women [4.0% (n = 328) vs 8.8% (n = 575)], as did 65–69-year-old subjects (2.2%; n = 49) compared with those 70–79 years old, 80–89 years old, and especially the oldest group, aged 90 or more [3.2% (n = 192), 8.3% (n = 437) and 19.7% (n = 225), respectively].

Being concomitantly affected by several diseases increased the prevalence of unplanned nursing home admission, from 1.8% (n = 5) for older adults with a single disease (ICD-10) to 6.8% (n = 797) for those with five or more diseases. Furthermore, the number of surgical interventions was negatively associated with the prevalence of unplanned nursing home admission. Patients who had not undergone surgery showed a higher probability of unplanned nursing home admission (7.8%; n = 379) than those who had undergone several interventions [3.5% (n = 39) for four interventions, 4.2% (n = 84) for five interventions] (Table 1). The number of drugs prescribed at hospital discharge showed a positive linear relationship with unplanned nursing home admission (gamma = 0.368) (Figure 1).

[Insert Table 1]

[Insert Figure 1]

Associations between unplanned nursing home admission and drugs

Bivariate associations showed that drugs were also related to unplanned nursing home admission (Table 2). On average, home-dwelling older adults discharged to a nursing home had more prescribed drugs than those returning to their home [10.9 (SD = 3.9) drugs vs 8.9 (SD = 3.2)]. Psycholeptics (antipsychotics, anxiolytics, hypnotics and sedatives) and psychoanaleptic drugs (antidepressants, psychostimulants, nootropics and anti-dementia drugs), antiemetics and antinauseants, anti-Parkinson's disease drugs, and drugs treating constipation and the sensory organs were significantly associated with unplanned nursing home admission. On the contrary, patients taking lipid-modifying agents were less prone to unplanned nursing home admission.

For peer review only - http://bmjopen.bmj.com/site/about/guidelines.xhtml

[Insert Table 2]

Multivariate baseline model

A baseline, GEE logistic regression model, including sociodemographic information, clinical data, and diseases, was computed to predict unplanned nursing home admission among discharged polymedicated older adult patients (Figure 2 and Supplementary Table 3); prescribed drugs at hospital discharge were not included. If the 95% confidence interval (CI) does not overlap the null value (e.g. OR = 1), then the higher the odds ratio, the more the variable contributes to unplanned nursing home admission. Men had a lower probability of unplanned nursing home admission than women (OR = 0.62; 95% CI 0.52 to 0.73). Patients' probability of unplanned nursing home admission increased with age (OR = 1.07 for each additional year of age; 95% CI 1.05 to 1.08). Impaired mobility, dependency in the ADL and cognitive impairment revealed their substantial impacts on unplanned nursing home admission (OR = 3.22; 95% CI 2.67 to 3.87; OR = 4.62; 95% CI 3.76 to 5.67; and OR = 3.75; 95% CI 3.06 to 4.59, respectively). Circulatory and infectious diseases were related to lower probabilities of unplanned nursing home admission (OR = 0.78; 95% CI 0.63 to 0.98, and OR = 0.38; 95% CI 0.20 to 0.70, respectively), whereas traumatic injuries were related to higher probabilities (OR = 1.58; 95% CI 1.25 to 2.01). The number of ICD-10 diagnoses alone had no significant impact on the odds of unplanned nursing home admission (OR = 1.11; 95% CI 0.98 to 1.24), in contrast to the number of surgical interventions undergone (CHOP), which was a protective factor against unplanned hospitalisation (OR = 0.95; 95% CI 0.90 to 0.99). The year of hospital stay also had a significant impact, with more recent stays having lower probabilities of unplanned nursing home admission (OR = 0.88; 95% CI 0.82 to 0.94, per ensuing year).

[Insert Figure 2]

Prediction of unplanned nursing home admission and drug prescription

A higher number of prescribed drugs was associated with a higher probability of unplanned nursing home admission (OR = 1.17; 95% CI 1.15 to 1.19). Figure 3 and Supplementary Table 4 present the baseline GEE logistic regression model shown in Figure 2 completed with those drugs prescribed to older adults at discharge that had a significant statistical association ($p < 0.05$) with unplanned nursing home admission. Drugs without a significant statistical association are not presented in Figure 3 for simplification purposes. Antiemetics and antinauseants (OR = 2.53; 95% CI 1.21 to 5.30 for each additional unit), digestives (OR = 1.78; 95% CI 1.09 to 2.90), psycholeptics (OR = 1.76; 95% CI 1.60 to 1.93), antiepileptics (OR = 1.49; 95% CI 1.25 to 1.79) and anti-Parkinson's disease drugs (OR = 1.40; 95% CI 1.12 to 1.75) were strongly linked to unplanned nursing home admission after controlling for

For peer review only - http://bmjopen.bmj.com/site/about/guidelines.xhtml

other parameters. On the contrary, taking lipid-metabolism-modifying agents was associated with lower probabilities of unplanned nursing home admission (OR = 0.73; 95% CI 0.60 to 0.90, for each extra drug from this class prescribed).

[Insert Figure 3]

Combined drug intake and probabilities of unplanned nursing home admission

To reduce collinearity and simplify the results, the combined intake of different ATC drug classes was recoded as a dichotomised variable for each drug pairing and added to the previous model [22]. Only the drugs and drug combinations prescribed to older adults at discharge that had significant associations ($p < 0.05$) with unplanned nursing home admission are presented. The combined intake of cardiac therapy and psychoanaleptic drugs was significantly associated with unplanned nursing home admission (OR = 1.87; 95% CI 1.11 to 3.16), as were psychoanaleptics and diabetes drugs combined (OR = 1.75; 95% CI 1.03 to 2.98), and psycholeptic drugs and vitamins combined (OR = 1.71; 95% CI 1.03 to 2.84). On the contrary, the combined intake of beta-blocking agents and antiepileptics strongly diminished the odds of unplanned nursing home admission (OR = 0.39; 95% CI 0.23 to 0.67).

We also investigated the risk of unplanned nursing home admission for combined drug intake within the same drug class. The combined intake of two or more antiemetic and antinauseants (OR = 2.65; 95% CI 1.26 to 5.58), psycholeptics (OR = 1.64; 95% CI 1.46 to 1.85), antiepileptics (OR = 1.55; 95% CI 1.23 to 1.96) or anti-Parkinson's disease drugs (OR = 1.44; 95% CI 1.13 to 1.83) were strongly associated with a higher probability of unplanned nursing home admission.

Supplementary Table 5 summarises the main findings from our predictive analysis.

Discussion

This population-based hospital registry study used longitudinal data to examine the unplanned nursing home admission of hospitalised polymedicated older inpatients, revealing a 6.1% prevalence rate over the four-year dataset, in agreement with previous work by Luppa *et al*. (men: 5.4%; women: 6.0%) and Goodwin *et al*. (5.5%) [7, 28]. The slight decrease in prevalence over the four years of the study may be explained by improvements in the regional home-care services' contribution to maintaining older adults at home, but also to planned nursing home admissions without the requirement for intermediate hospitalisation [29]. Furthermore, the number of places in the region's nursing homes increased in that period [30], allowing people for whom care at home became impossible to be admitted to a nursing home more promptly.

For peer review only - http://bmjopen.bmj.com/site/about/guidelines.xhtml

Our predictive analysis revealed that the group of the oldest adults, presenting functional mobility impairments, dependency in the ADL and cognitive impairment, was also at a high risk of unplanned nursing home admission, which is consistent with previous retrospective and prospective studies [31, 32]. Very old inpatients (≥ 90 years old) were much more likely to have an unplanned nursing home admission than those aged 65–69 (19.7% vs 2.2%). This finding was expected and matched with previous research [33], bearing in mind that the very oldest group presented with a high prevalence of multimorbidity and advanced functional and cognitive impairments. Unexpectedly, regardless of age, our results showed that older women had a higher prevalence and probability of unplanned nursing home admission than men [34]. Yet, our data could not entirely explain this result. Previous publications have indicated that social and life-expectancy factors may play roles in the different rates of nursing home admission between older adult men and women [34].

Our findings highlighted that functional and cognitive impairments were strong risk factors for unplanned nursing home admission, which is in line with the studies by Luppa *et al*. and Goodwin *et al*. [28, 34]. Likewise, our results emphasised a high risk of unplanned nursing home admission among non-surgically treated and trauma patients. This could be explained by the relationship between orthopaedic guidelines on traumatic injuries among older adults that suggest avoiding surgery, for several medical reasons (number and severity of multimorbidities), and which may lead to increased functional impairment and unplanned nursing home admission, as suggested by Gardner *et al*. and Cutugno [35, 36].

As might be expected, older adults who underwent an unplanned nursing home admission had more prescribed drugs than those returning home. Our results were in line with the retrospective study by Lucchetti *et al*., which demonstrated a relationship between the prescription of cardiovascular, gastrointestinal, and metabolic drugs and unplanned nursing home admission [37].

Our findings indicated that patients prescribed more than one drug from the same class of drugs—from the classes of antiemetics and antinauseants, psycholeptics, antiepileptics, or anti-Parkinson's disease drugs—had a higher risk of discharge to a nursing home. Although this phenomenon is still under-investigated, our findings are not in line with the few existing studies in this area, which have presented no significant relationships between drug interactions and unplanned nursing home admission [38]. However, in hospital settings, a recent systematic review reported drug–drug interactions among 80% or more of older inpatients [39]. Since polymedicated older inpatients should be considered as a population at a high risk of adverse outcomes, further studies should investigate how drug–drug interactions might predict the risks of nursing home admission.

For peer review only - http://bmjopen.bmj.com/site/about/guidelines.xhtml

Our findings undeniably mirrored existing evidence that chronic conditions and debilitating comorbidities are significant risk factors for unplanned nursing home admission [3, 7]. However, they also raised questions regarding hospitalisation's effects on the individual ageing process, which likely interact to produce a cascade of factors towards functional decline and dependency [1]. The adverse effects of hospitalisation begin immediately and progress rapidly [1]. Harrison *et al*. and Haaksma *et al*. described ways in which acute and exacerbated acute and chronic disorders, reinforced by existing undiagnosed geriatric syndromes (frailty, delirium, pressure sores, functional incontinence), contributed to hospitalised older patients being unable to return home and needing to be discharged to a nursing home [2, 16]. Previous studies suggested that silent geriatric syndromes such as frailty and functional decline, together with polypharmacy, are not only clinically characteristic of older adults but also potential predictors of being at risk of a further loss of independence and subsequent nursing home admission. Montes *et al*. pointed out the dramatic rise in numbers of frail, hospitalised older adults. This increase generates concerns about whether nursing homes—already suffering from long admission waiting lists of home-dwelling older adults—will be able to cope with older adults' complex care needs [40].

Although some of the predisposing predictors identified cannot be treated (i.e. sex, age), they may still contribute to an older adult's risk of being discharged to a nursing home and subsequently exacerbate their situation there. Given that hospitalisation introduces stressors that may increase the chances of unplanned nursing home admission [41, 42], using patients' electronic hospital data could help identify the high-risk older adults who would benefit from specific preventive interventions. Being able to rapidly identify inpatients at a high risk of unplanned nursing home admission may help professional caregivers to provide them with the appropriate community-health resources, such as community-based rehabilitation programmes. This would help older people to remain in their community for longer.

Study strengths and limitations

Although our population-based study's findings could be generalised to other regions of Switzerland, any interpretations should be made with caution. The Swiss Federal Statistical Office collects minimal annual data from public and private hospitals (number of hospitalisations, ICD-diagnoses, length of stay, place of discharge, age and sex), but these indicated that our data were similar to those from other cantons with analogous healthcare structures [43]. However, we did not have access to more detailed data with which to compare with our dataset and explore potential biases or significant differences. Nevertheless, the Valais Hospital is the third largest hospital in Switzerland with more than 1,000 beds and over 35,000 hospitalisations per year. Therefore, our findings could provide

information to help better define which integrated healthcare approaches could be implemented to attenuate the risk factors associated with unplanned nursing home admission following an acute hospital admission or readmission. The numerous predictors revealed in our study enabled us to conceptualise an overview of hospitalised older adults' health conditions before their unplanned nursing home admission. As healthcare moves towards ever-more personalised medicine, this result could help create more refined, tailored, future interventions via 'risk profiles' defined using each older adult's personal predictors.

Our study had some limitations. The absence of data on patients' functional status before hospital admission meant that we could not assess changes to that status during hospitalisation, such as the influence of the development or deterioration of functional and cognitive impairment. We did not compute analysis on specific disorders such as neurodegenerative diseases like dementia and Parkinson's disease because this was beyond the scope of our study protocol. However, further analyses could confirm earlier studies showing that these diseases significantly affect a person's risk of nursing home admission after hospitalisation, with almost 90% of patients with dementia being admitted into a nursing home before dying [15, 16]. Additionally, our dataset was based on routinely collected data, and we were unable to control for potential data assessment errors made by the Valais Hospital's healthcare staff at discharge. Moreover, we were unable to assess deceased patients' death certificates as these were unavailable and beyond the scope of our study. Although the study considered statistical associations between drugs and unplanned nursing home admission, it did not use clinically diagnosed drug–drug interactions. Lastly, our data were unable to identify hospitalisations that might have been triggered by limited care options at home or hospitalisations that were necessary while awaiting a place in a nursing home. These cases of planned nursing home admissions could not be distinguished from the unplanned nursing home admissions considered in the study. In addition, some patients may not have been transferred directly from hospital to nursing homes and may have had to stay in an intermediate structure while awaiting a place. These patients were not included in the study due to the unavailability of this information in the database.

Conclusion

The sociodemographic characteristics of hospitalised older inpatients, together with their clinical and medical conditions and their prescribed drugs, can provide us with a significant set of risk factors for unplanned nursing home admission, sustaining our stated hypotheses. Identifying these risk factors for unplanned nursing home admission could be of great assistance in developing predictive tools and tailored intervention programmes aimed at reducing the number of older adults placed in nursing homes. Our results showed that the patient-related risk factors leading to nursing home admission

For peer review only - http://bmjopen.bmj.com/site/about/guidelines.xhtml

were based on declines in physical and cognitive function. Treatment with single drugs and combinations of drugs were also associated with unplanned nursing home admission, indicating that multiple chronic health conditions are important risk factors of a non-return home. Our findings may help to identify those older inpatients at the greatest risk of unplanned nursing home admission, enabling their care to be optimised by counterbalancing those risk factors. Further research is required across large samples of older inpatients to investigate whether tailored interventions at early stages in chronic diseases could delay physical and cognitive dysfunction and reduce unplanned nursing home admissions among this growing segment of the population.

Acknowledgement

The authors thank the partner hospital, including the hospital's data warehouse, for its valuable contributions.

Ethics approval and patient consent

Ethical approval was obtained from the Human Research Ethics Committee of the Canton of Vaud (CER-VD, 2018-02196), thus permitting our partner hospital's data warehouse to provide the appropriate dataset. Given the retrospective data source, obtaining consent from the patients concerned was impossible or posed disproportionate difficulties. The present study respects the legal requirements for research projects involving data re-use without consent, as set out in Art. 34 from the Swiss Human Research Act (HTA).

Conflict of Interest Statement

The authors have no conflicts of interest to declare.

Funding Sources

This work was supported by Swiss National Science Foundation grant number 407440_183434/1.

Author Contributions

BW, FP, and HV had the original idea. BW, MdRC, MMM and HV provided conceptual and methodological expertise to the study design and BW, FP, CMM, AvG, and HV to data analysis and interpretation. BW, FP, and HV were major contributors to writing the manuscript. All authors read, edited, and approved the final manuscript.

Data Availability Statement

As part of the Data Use Agreement, authors are not allowed to provide raw data. Upon a reasonable request, the corresponding author will provide statistical programming code used to generate results.

For peer review only - http://bmjopen.bmj.com/site/about/guidelines.xhtml

445

Figure Legends

Figure 1. Relationship between unplanned nursing home admission and number of prescribed drugs at discharge.

Figure 2. Baseline GEE logistic regression model with unplanned nursing home admission as the dependent variable associated with sociodemographic, hospitalisation, and independent clinical and medical variables (N = 14,705 observations for 9,430 different subjects).

Figure 3. The GEE logistic regression model of the drugs prescribed to older adults at discharge with significant predictive values (odds ratios) for unplanned nursing home admission (N = 14,705 observations for 9,430 different subjects)—controlled for the parameters of the baseline model.

446

For peer review only

For peer review only - http://bmjopen.bmj.com/site/about/guidelines.xhtml

References

1. Tasheva P, Vollenweider P, Kraege V, Roulet G, Lamy O, Marques-Vidal P, et al. Association Between Physical Activity Levels in the Hospital Setting and Hospital-Acquired Functional Decline in Elderly Patients. JAMA Netw Open. 2020;3(1):e1920185.
2. Harrison JK, Garrido AG, Rhynas SJ, Logan G, MacLullich AM, MacArthur J, et al. New institutionalisation following acute hospital admission: a retrospective cohort study. Age Ageing. 2017;46(2):238-44.
3. Gaugler JE, Duval S, Anderson KA, Kane RL. Predicting nursing home admission in the U.S: a meta-analysis. BMC Geriatr. 2007;7:13.
4. Oseran AS, Lage DE, Jernigan MC, Metlay JP, Shah SJ. A "Hospital-Day-1" Model to Predict the Risk of Discharge to a Skilled Nursing Facility. J Am Med Dir Assoc. 2019;20(6):689-95 e5.
5. Huang H-H, Chang JC-Y, Tseng C-C, Yang Y-J, Fan J-S, Chen Y-C, et al. Comprehensive geriatric assessment in the emergency department for the prediction of readmission among older patients: A 3-month follow-up study. Archives of Gerontology and Geriatrics. 2021;92:104255.
6. Middleton A, Li S, Kuo YF, Ottenbacher KJ, Goodwin JS. New Institutionalization in Long-Term Care After Hospital Discharge to Skilled Nursing Facility. J Am Geriatr Soc. 2018;66(1):56-63.
7. Luppa M, Luck T, Weyerer S, Konig HH, Brahler E, Riedel-Heller SG. Prediction of institutionalization in the elderly. A systematic review. Age Ageing. 2010;39(1):31-8.
8. Bellelli G, Magnifico F, Trabucchi M. Outcomes at 12 months in a population of elderly patients discharged from a rehabilitation unit. J Am Med Dir Assoc. 2008;9(1):55-64.
9. Fogg C, Griffiths P, Meredith P, Bridges J. Hospital outcomes of older people with cognitive impairment: An integrative review. Int J Geriatr Psychiatry. 2018;33(9):1177-97.
10. Landefeld CS, Palmer RM, Kresevic DM, Fortinsky RH, Kowal J. A randomized trial of care in a hospital medical unit especially designed to improve the functional outcomes of acutely ill older patients. N Engl J Med. 1995;332(20):1338-44.
11. Ferrucci L, Guralnik JM, Pahor M, Corti MC, Havlik RJ. Hospital diagnoses, Medicare charges, and nursing home admissions in the year when older persons become severely disabled. JAMA. 1997;277(9):728-34.
12. Ferrucci L, Bandinelli S, Benvenuti E, Di Iorio A, Macchi C, Harris TB, et al. Subsystems contributing to the decline in ability to walk: bridging the gap between epidemiology and geriatric practice in the InCHIANTI study. J Am Geriatr Soc. 2000;48(12):1618-25.
13. Koirala B, Hansen BR, Hosie A, Budhathoki C, Seal S, Beaman A, et al. Delirium point prevalence studies in inpatient settings: A systematic review and meta-analysis. Journal of clinical nursing. 2020;29(13-14):2083-92.
14. Welch C, McCluskey L, Wilson D, Chapman GE, Jackson TA, Treml J, et al. Delirium is prevalent in older hospital inpatients and associated with adverse outcomes: results of a prospective multi-centre study on World Delirium Awareness Day. BMC Medicine. 2019;17(1):229.
15. Zekry D, Herrmann FR, Grandjean R, Vitale AM, De Pinho MF, Michel JP, et al. Does dementia predict adverse hospitalization outcomes? A prospective study in aged inpatients. Int J Geriatr Psychiatry. 2009;24(3):283-91.
16. Haaksma ML, Rizzuto D, Ramakers I, Garcia-Ptacek S, Marengoni A, van der Flier WM, et al. The Impact of Frailty and Comorbidity on Institutionalization and Mortality in Persons With Dementia: A Prospective Cohort Study. J Am Med Dir Assoc. 2019;20(2):165-70 e2.
17. Fried TR, O'Leary J, Towle V, Goldstein MK, Trentalange M, Martin DK. Health outcomes associated with polypharmacy in community-dwelling older adults: a systematic review. Journal of the American Geriatrics Society. 2014;62(12):2261-72.
18. Wimmer BC, Dent E, Visvanathan R, Wiese MD, Johnell K, Chapman I, et al. Polypharmacy and medication regimen complexity as factors associated with hospital discharge destination among older people: a prospective cohort study. Drugs Aging. 2014;31(8):623-30.

For peer review only

For peer review only - http://bmjopen.bmj.com/site/about/guidelines.xhtml

19. Koppitz AL, Dreizler J, Altherr J, Bosshard G, Naef R, Imhof L. Relocation experiences with unplanned admission to a nursing home: a qualitative study. Int Psychogeriatr. 2017;29(3):517-27.
20. Pereira F, Roux P, Santiago-Delefosse M, von Gunten A, Wernli B, Martins MM, et al. Optimising medication management for polymedicated home-dwelling older adults with multiple chronic conditions: a mixed-methods study protocol. BMJ Open. 2019;9(10):e030030.
21. Swiss Federal Statistical Office. Swiss Classification of Surgical Procedures (CHOP). 2017. Report No.: 9783303142851.
22. WHO. The Anatomical Therapeutic Chemical Classification System with Defined Daily Doses (ATC/DDD) Geneva: World Health Organization; 2014 [24 March 2020]. Available from: http://www.who.int/classifications/atcddd/en/.
23. Olsen J. Register-based research: some methodological considerations. Scand J Public Health. 2011;39(3):225-9.
24. Taushanov Z, Verloo H, Wernli B, Giovanni SD, Gunten Av, Pereira F. Transforming a Patient Registry Into a Customized Data Set for the Advanced Statistical Analysis of Health Risk Factors and for Medication-Related Hospitalization Research: Retrospective Hospital Patient Registry Study. JMIR Medical Informatics. 2021;9(5).
25. Andrade C. Age as a variable: Continuous or categorical? Indian J Psychiatry. 2017;59(4):524-5.
26. Haider SI, Johnell K, Thorslund M, Fastbom J. Trends in polypharmacy and potential drug-drug interactions across educational groups in elderly patients in Sweden for the period 1992 - 2002. International journal of clinical pharmacology and therapeutics. 2007;45(12):643-53.
27. Bujang MA, Sa'at N, Bakar TMITA. Sample size guidelines for logistic regression from observational studies with large population: emphasis on the accuracy between statistics and parameters based on real life clinical data. The Malaysian journal of medical sciences: MJMS. 2018;25(4):122.
28. Goodwin JS, Howrey B, Zhang DD, Kuo YF. Risk of continued institutionalization after hospitalization in older adults. J Gerontol A Biol Sci Med Sci. 2011;66(12):1321-7.
29. Gillès De Pélichy E, Verloo H, Gasser J, Monod S. Intérêts et nécessité du renfort des soins à domicile dans le cadre des situations de crise psychique-Le résultat d'une politique de santé publique forte. Rev Med Suisse. 2020:1741-4.
30. Observatoire valaisan de la santé. Statistique cantonale des établissements médico-sociaux (EMS). OVS; 2020.
31. Salminen M, Laine J, Vahlberg T, Viikari P, Wuorela M, Viitanen M, et al. Factors associated with institutionalization among home-dwelling patients of Urgent Geriatric Outpatient Clinic: a 3-year follow-up study. Eur Geriatr Med. 2020;11(5):745-51.
32. Yoo JW, Nakagawa S, Kim S. Delirium and transition to a nursing home of hospitalized older adults: a controlled trial of assessing the interdisciplinary team-based "geriatric" care and care coordination by non-geriatrics specialist physicians. Geriatr Gerontol Int. 2013;13(2):342-50.
33. Del Ducal GF, da Silva SG, Thumél E, Santos IS, Hallal PC. Predictive factors for institutionalization of the elderly: a case-control study. Rev Saúde Pública 2012;46(1).
34. Luppa M, Luck T, Weyerer S, Konig HH, Riedel-Heller SG. Gender differences in predictors of nursing home placement in the elderly: a systematic review. Int Psychogeriatr. 2009;21(6):1015-25.
35. Gardner RL, Harris F, Vittinghoff E, Cummings SR. The risk of fracture following hospitalization in older women and men. Arch Intern Med. 2008;168(15):1671-7.
36. Cutugno CL. The 'Graying' of Trauma Care: Addressing Traumatic Injury in Older Adults. Am J Nurs. 2011;111(11):40-8.
37. Lucchetti G, Granero AL, Pires SL, Gorzoni ML. Fatores associados à polifarmácia em idosos institucionalizados. Revista Brasileira de Geriatria e Gerontologia. 2010;13:51-8.

For peer review only

For peer review only - http://bmjopen.bmj.com/site/about/guidelines.xhtml

38. Castilho ECD, Reis A, Borges T, Siqueira L, Miasso A. Potential drug–drug interactions and polypharmacy in institutionalized elderly patients in a public hospital in Brazil. Journal of psychiatric and mental health nursing. 2018;25(1):3-13.
39. de Oliveira LM, Diel JdAC, Nunes A, Dal Pizzol TdS. Prevalence of drug interactions in hospitalised elderly patients: a systematic review. European Journal of Hospital Pharmacy. 2021;28(1):4-9.
40. Montes Reula L, Cañete Lairla M, Navarro López J, Pelegrín Valero C, Galindo Ortiz de Landázuri J, Marijuán Fernández P, et al. Predominant factors of institutionalization in the elderly: a comparative study between home nursing and community dwelling. Working with Older People. 2021;25(1):58-72.
41. Carvalho TC, Valle APd, Jacinto AF, Mayoral VFdS, Boas PJFV. Impact of hospitalization on the functional capacity of the elderly: A cohort study. Revista Brasileira de Geriatria e Gerontologia. 2018;21(2):134-42.
42. Covinsky KE, Pierluissi E, Johnston CB. Hospitalization-associated disability:“She was probably able to ambulate, but I’m not sure”. Jama. 2011;306(16):1782-93.
43. Marquis J-F, Andreani T. Santé: Statistique de poche 2020. Neuchâtel: Office fédéral de la statistique (OFS); 2020 2021. Contract No.: 1541-2000.

For peer review only

For peer review only - http://bmjopen.bmj.com/site/about/guidelines.xhtml

Table 1. Prevalence of unplanned nursing home admissions with regards to associations with sociodemographic characteristics and clinical and medical conditions among polymedicated hospitalised older adults (N = 14,705)

Variables	Unplanned nursing home admission, n (%)	*p*-value
Overall sample of older adults (n = 14,705)	903 (6.1)	
Sex		< 0.001
Female/Male	575 (8.8)/328 (4.0)	
Age in years		< 0.001
65–69 years	49 (2.2)	
70–79 years	192 (3.2)	
80–89 years	437 (8.3)	
90 years or more	225 (19.7)	
Mobility		< 0.001
Full ability (0) / impairment (1)	214 (2.0)/689 (16.7)	
Dependence in the activities of daily living		< 0.001
Full ability (0)/impairment (1)	472 (3.4)/431 (44.8)	
Mental status		< 0.001
Full ability (0)/impairment (1)	531 (3.8)/ 372 (41.3)	
ICD-10 principal diagnosis: circulatory problems		< 0.001
No (0)/Yes (1)	752 (6.7)/ 151 (4.3)	
ICD-10 principal diagnosis: infection		0.003
No (0)/Yes (1)	892 (6.2)/ 11 (2.7)	
ICD-10 principal diagnosis: respiratory problems		0.226
No (0)/Yes (1)	797 (6.1)/106 (6.8)	
ICD-10 principal diagnosis: traumatic injuries		< 0.001
No (0)/Yes (1)	720 (5.3)/183 (14.9)	
ICD-10 principal diagnosis: tumour		0.001
No (0)/Yes (1)	835 (6.4)/ 68 (4.3)	
Number of ICD-10 diseases		< 0.001
1	5 (1.8)	
2	17 (2.9)	
3	37 (3.9)	
4	47 (3.9)	
5 or more	797 (6.8)	
Number of surgical interventions (CHOP)		< 0.001
0	379 (7.8)	
1	187 (6.4)	
2	135 (5.8)	
3	79 (5.2)	
4	39 (3.5)	
5 or more	84 (4.2)	
Year of hospitalisation		0.002
2015	276 (7.3)	
2016	216 (6.1)	
2017	194 (5.2)	
2018	217 (5.9)	

For peer review only - http://bmjopen.bmj.com/site/about/guidelines.xhtml

Number of drugs at hospital discharge	10.91 (SD = 3.89)	< 0.001*

Note. * Mann–Whitney U test

Table 2. Prevalence of unplanned nursing home admission among polymedicated hospitalised older adults (N = 14,705) with regards to associations with different classes of prescribed drugs

**Drugs (ATC code)**	**Unplanned nursing home admission**		
	**No drugs in this class n (%)**	**Drugs in this class n (%)**	***p*-value**
First level, main anatomical group			
Blood and blood-forming organ drugs (B)	180 (5.4)	723 (6.4)	0.050
Dermatologicals (D)	828 (5.8)	75 (14.1)	< 0.001
Genito-urinary system and sex hormones (G)	737 (6.1)	6.3%	0.699
Systemic hormonal preparations, excluding sex hormones and insulins (H)	737 (6.1)	6.5%	0.403
Anti-infectives for systemic use (J)	736 (6.4)	167 (5.3)	0.020
Antineoplastic and immunomodulating agents (L)	881 (6.3)	22 (3.5)	0.005
Drugs for the musculoskeletal system (M)	815 (6.4)	88 (4.3)	< .001
Antiparasitic products, insecticides and repellents (P)	893 (6.2)	10 (4.0)	0.144
Respiratory system drugs (R)	771 (6.3)	132 (5.5)	0.147
Sensory organ drugs (S)	752 (5.5)	151 (13.4)	< 0.001
Second level, therapeutic subgroup			
Stomatological preparations (A01)	899 (6.1)	4 (7.5)	0.669
Drugs for acid-related disorders (A02)	384 (5.8)	519 (6.4)	0.136
Drugs for functional gastrointestinal disorders (A03	805 (5.9)	98 (9.8)	< 0.001
Antiemetics and antinauseants (A04)	884 (6.1)	19 (18.6)	< 0.001
Bile and liver therapy drugs (A05)	900 (6.1)	3 (7.9)	0.652
Drugs for constipation (A06)	605 (4.8)	298 (13.5)	< 0.001
Antidiarrheals, intestinal anti-inflammatory/anti-infective agents (A07)	863 (6.0)	40 (9.4)	0.005
Digestives, including enzymes (A09)	883 (6.1)	20 (8.4)	0.148
Diabetes drugs (A10)	804 (6.6)	99 (3.9)	< 0.001
Vitamins (A11)	801 (6.2)	102 (5.9)	0.629
Mineral supplements (A12)	513 (4.8)	390 (9.6)	< 0.001
Other alimentary tract and metabolism products (A16)	901 (6.1)	2 (5.9)	0.950
Cardiac therapy drugs (C01)	792 (6.1)	111 (6.3)	0.792
Antihypertensives (C02)	888 (6.2)	15 (4.6)	0.237
Diuretics (C03)	621 (5.5)	282 (8.1)	< 0.001
Peripheral vasodilators (C04)	901 (6.1)	2 (4.2)	0.568
Vasoprotectives (C05)	884 (6.1)	19 (7.2)	0.471
Beta blocking agents (C07)	588 (7.2)	315 (4.8)	< 0.001
Calcium channel blockers (C08)	762 (6.1)	141 (6.1)	0.964
Agents acting on the renin-angiotensin system (C09)	472 (7.2)	431 (5.3)	< 0.001
Lipid-modifying agents (C10)	720 (8.2)	183 (3.1)	< 0.001
Anaesthetics (N01)	898 (6.1)	5 (13.5)	0.061
Analgesics (N02)	158 (3.6)	745 (7.2)	< 0.001
Antiepileptics (N03)	753 (5.7)	150 (10.3)	< 0.001
Drugs against Parkinson's disease (N04)	814 (5.7)	89 (18.1)	< 0.001
Psycholeptics (N05)	201 (2.4)	702 (11.0)	< 0.001
Psychoanaleptics (N06)	565 (4.8)	338 (11.9)	< 0.001

For peer review only

For peer review only - http://bmjopen.bmj.com/site/about/guidelines.xhtml

Other nervous system drugs (N07)	881 (6.1)	22 (5.9)	0.813

For peer review only

For peer review only - http://bmjopen.bmj.com/site/about/guidelines.xhtml

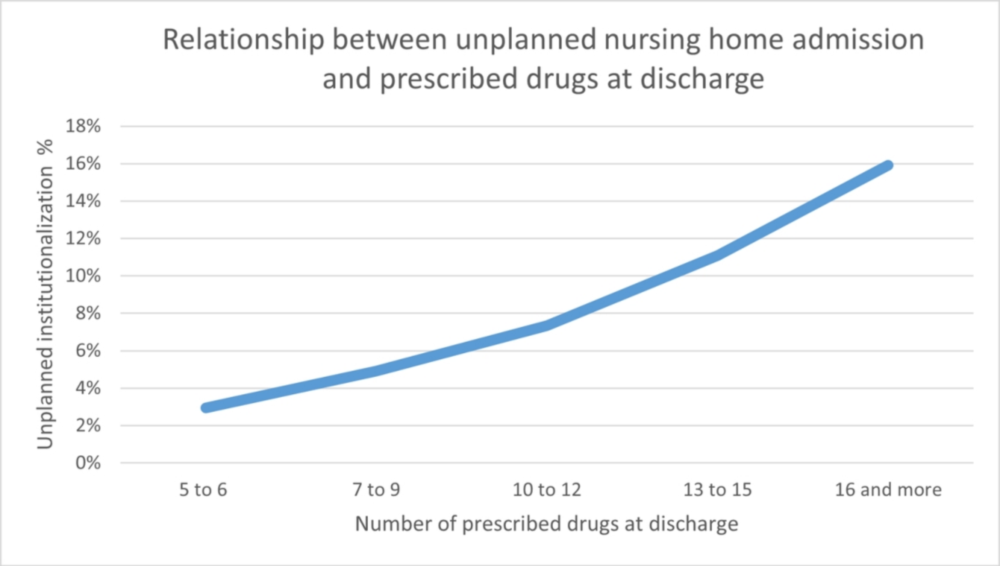

Relationship between unplanned nursing home admission and number of prescribed drugs at discharge.

125x71mm (300 x 300 DPI)

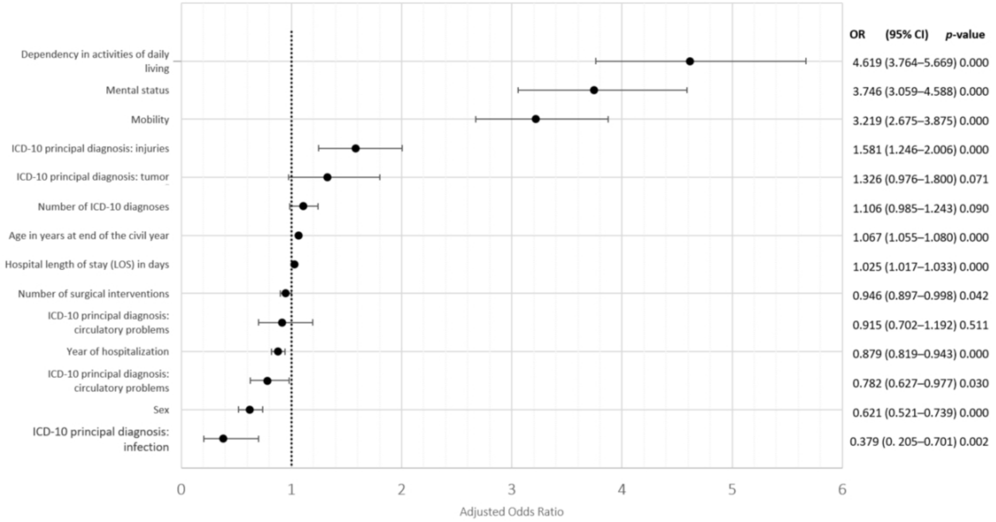

Baseline GEE logistic regression model with unplanned nursing home admission as the dependent variable associated with sociodemographic, hospitalisation, and independent clinical and medical variables (N = 14,705 observations for 9,430 different subjects).

107x56mm (300 x 300 DPI)

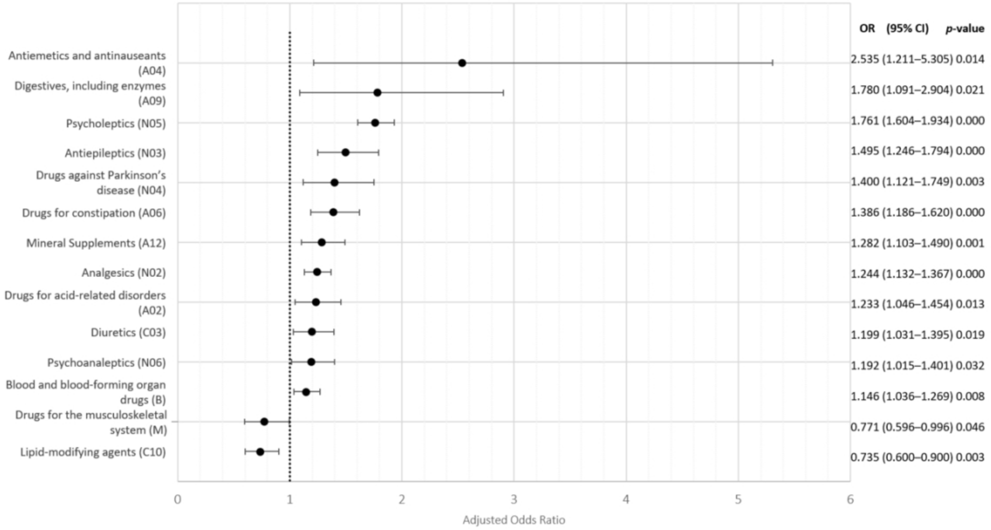

The GEE logistic regression model of the drugs prescribed to older adults at discharge with significant predictive values (odds ratios) for unplanned nursing home admission (N = 14,705 observations for 9,430 different subjects).

109x57mm (300 x 300 DPI)

Supplementary Table 1. Descriptive statistics of the older adult inpatients' health status (N = 14,705 observations for 9,430 different subjects).

**Variables**	**Population description** (N = 14,705)
Sex	
Men n (%)	8,088 (55)
Women n (%)	6,617 (45)
Age (years)	
Mean (SD)	78.16 (7.65)
Hospital length of stay (days)	
Mean (SD)	8.63 (7.58)
Mobility	
Full ability n (%)	6,825 (63.2)
Impairment n (%)	7,880 (36.8)
Activities of Daily Living	
Full ability n (%)	12,131 (87.4)
Impairment n (%)	2,574 (12.6)
Cognitive status	
Full ability n (%)	12,622 (89.8)
Impairment n (%)	2,083 (10.2)
ICD-10 diseases (number)[1]	
Mean (SD)	4.59 (0.91)
Surgical interventions performed (CHOP) Mean (SD)	1.80 (1.77)
Most prevalent ICD-10	
Circulatory diseases n (%)	4,788 (23.4)
Infectious n (%)	559 (2.7)
Respiratory diseases n (%)	2,111 (10.3)
Traumatic injuries n (%)	2,385 (11.7)
Tumours n (%)	2,041 (10.0)

[1] Each older adult's number of ICD-10 diseases was entered into the model as a proxy for multimorbidity.

Supplementary Table 2. Descriptive statistics of prescribed drugs at discharge based on the ATC among the polymedicated older inpatients (N = 14,705 observations for 9,430 different subjects).

Drugs by ATC, level 2	Number of drugs per patient	
	Min–Max	Mean (SD)
First level, anatomical main group		
Blood and blood-forming organ drugs (B)	0–6	1.16 (0.86)
Dermatologicals (D)	0–3	0.04 (0.22)
Genito urinary system and sex hormones (G)	0–4	0.21 (0.47)
Systemic hormonal preparations, excl. sex hormones and insulins (H)	0–4	0.20 (0.46)
Anti-infective for systemic use (J)	0–4	0.23 (0.46)
Antineoplastic and immunomodulating agents (L)	0–5	0.05 (0.23)
Musculo skeletal system drugs (M)	0–3	0.15 (0.39)
Antiparasitic products, insecticides and repellents (P)	0–2	0.02 (0.13)
Respiratory system drugs (R)	0–7	0.27 (0.72)
Sensory organ drugs (S)	0–6	0.10 (0.40)
Second level, therapeutic subgroup		
Stomatological preparations (A01)	0–1	0.01 (0.06)
Drugs for acid related disorders (A02)	0–3	0.56 (0.52)
Drugs for functional gastrointestinal disorders (A03)	0–3	0.07 (0.28)
Antiemetics and antinauseants (A04)	0–1	0.01 (0.08)
Bile and liver therapy drugs (A05)	0–1	0.01 (0.05)
Drugs for constipation (A06)	0–4	0.17 (0.42)
Anti-diarrhoeal, intestinal anti-inflammatory/anti-infective agents (A07)	0–2	0.03 (0.18)
Digestives, incl. enzymes (A09)	0–2	0.02 (0.13)
Drugs used in diabetes (A10)	0–5	0.25 (0.63)
Vitamins (A11)	0–4	0.15 (0.44)
Mineral supplements (A12)	0–3	0.30 (0.51)
Other alimentary tract and metabolism products (A16)	0–1	0.01 (0.05)
Cardiac therapy drugs (C01)	0–4	0.14 (0.41)
Antihypertensives (C02)	0–2	0.02 (0.17)
Diuretics (C03)	0–3	0.28 (0.54)
Peripheral vasodilators (C04)	0–1	0.01 (0.06)
Vaso-protectives (C05)	0–3	0.02 (0.14)
Beta-blocking agents (C07)	0–2	0.45 (0.51)
Calcium channel blockers (C08)	0–2	0.16 (0.37)
Agents acting on the Renin-Angiotensin system (C09)	0–3	0.63 (0.62)
Lipid Modifying agents (C10)	0-3	0.41 (0.52)
Anaesthetics (N01)	0–1	0.01 (0.05)
Analgesics (N02)	0–7	1.03 (0.91)
Antiepileptics (N03)	0–5	0.11 (0.36)
Anti-Parkinson drugs (N04)	0–5	0.04 (0.25)
Psycholeptics (N05)	0–7	0.57 (0.77)
Psychoanaleptics (N06)	0–3	0.21 (0.45)
Other nervous system drugs (N07)	0–3	0.03 (0.19)
Total number of drugs	5–32	9.07 (3.32)
N valid - listwise		14.70

For peer review only

Supplementary Table 3. Baseline, GEE logistic regression model with unplanned nursing home admission as the dependent variable associated with sociodemographic, hospitalisation, and independent clinical and medical variables (N = 14,705 observations for 9,430 different subjects).

Variables	Odds Ratio	*p* > z	95%Confidence Interval
Sex [1]	0.62	< 0.000	0.52–0.74
Age in years	1.07	< 0.000	1.05–1.08
Hospital length of stay (LOS) in days	1.02	< 0.000	1.02–1.03
Mobility [2]	3.22	< 0.000	2.67–3.87
Dependency in the activities of daily living [2]	4.62	< 0.000	3.76–5.67
Mental status [2]	3.75	< 0.000	3.06–4.59
ICD-10 principal diagnosis: circulatory problems [3]	0.78	0.030	0.63–0.98
ICD-10 principal diagnosis: infection [3]	0.38	0.002	0.20–0.70
ICD-10 principal diagnosis: respiratory problems [3]	0.91	0.511	0.70–1.19
ICD-10 principal diagnosis: injuries [3]	1.58	< 0.000	1.25–2.01
ICD-10 principal diagnosis: tumour [3]	1.33	0.071	0.98–1.80
Number of ICD-10 diagnoses	1.11	0.090	0.98–1.24
Number of surgical interventions (CHOP)	0.95	0.042	0.90–0.99
Number of prescribed drugs	1.17	0.000	1.15–1.19
Year of hospitalisation: 2015 to 2018	0.88	< 0.000	0.82–0.94

Note. 1: 0 = woman, 1 = man; 2: 0 = normal status, 1 = poor status; 3: 0 = no, 1 = yes

For peer review only

Supplementary Table 4. GEE logistic regression model of the drugs prescribed to older adults at discharge with significant predictive values (odds ratios) for unplanned nursing home admission (N = 14,705 observations for 9,430 different subjects).

**Drugs**	**Odds Ratio**	***p* > z**	**95% Confidence Interval**
Antiemetics and antinauseants (A04)	2.53	0.014	1.21–5.30
Digestives, including enzymes (A09)	1.78	0.021	1.09–2.90
Psycholeptics (N05)	1.76	0.000	1.60–1.93
Antiepileptics (N03)	1.49	0.000	1.25–1.79
Anti-Parkinson drugs (N04)	1.40	0.003	1.12–1.75
Drugs for constipation (A06)	1.39	0.000	1.19–1.62
Mineral Supplements (A12)	1.28	0.001	1.10–1.49
Analgesics (N02)	1.24	0.000	1.13–1.37
Drugs for acid-related disorders (A02)	1.23	0.013	1.05–1.45
Diuretics (C03)	1.20	0.019	1.03–1.39
Psychoanaleptics (N06)	1.19	0.032	1.01–1.40
Blood and blood-forming organ drugs (B)	1.15	0.008	1.04–1.27
Drugs for the musculoskeletal system (M)	0.77	0.046	0.60–0.99
Lipid-modifying agents (C10)	0.73	0.003	0.60–0.90

For peer review only

Supplementary Table 5. Factors associated with a higher and lower probability of unplanned nursing home admission among polymedicated hospitalised older adults (N = 14,705): summary of the predictive analysis.

**Risk factors for a higher probability of unplanned nursing home admission**
- Dependency in the activities of daily living (OR = 4.62, 95% CI: 3.76–5.67)
- Cognitive impairment (OR = 3.75, 95% CI: 3.06–4.59)
- Functional mobility impairment (OR = 3.22, 95% CI: 2.67–3.87)
- Antiemetics/antinauseants (OR = 2.53, 95% CI: 1.21–5.30)
- Digestives (OR = 1.78, 95% CI: 1.09–2.90)
- Psycholeptics (OR = 1.76, 95% CI: 1.60–1.93)
- Injuries (OR = 1.58, 95% CI: 1.25–2.01)
- Antiepileptics (OR = 1.49, 95% CI: 1.25–1.79)
- Anti-Parkinson's drugs (OR = 1.40, 95% CI: 1.12–1.75)
- Number of drugs prescribed (OR = 1.17, 95% CI: 1.15–1.19)
- Older age (OR = 1.07, 95% CI: 1.05–1.08)
Combined intake of:<br>- cardiac and psychoanaleptic drugs (OR = 1.87, 95% CI: 1.11–3.16)<br>- psychoanaleptic and diabetes drugs (OR = 1.75, 95% CI: 1.03–2.98)<br>- psycholeptic drugs and vitamins (OR = 1.71, 95% CI: 1.03–2.84)
Combined intake of two or more:<br>- antiemetics and antinauseants (OR = 2.65, 95% CI: 1.26–5.58)<br>- psycholeptics (OR = 1.64, 95% CI: 1.46–1.85)<br>- antiepileptics (OR = 1.55, 95% CI: 1.23–1.96)<br>- anti-Parkinson's drugs (OR = 1.44, 95% CI: 1.13–1.83)
**Protective factors for a lower probability of unplanned nursing home admission**
- Surgical interventions (OR = 0.95, 95% CI: 0.90–0.99)
- Circulatory diseases (OR = 0.78, 95% CI: 0.63–0.98)
- Lipid metabolism modifying agents (OR = 0.73, 95% CI: 0.60–0.90)
- Male sex (OR = 0.62; 95% CI: 0.52–0.73)
- Combined intake of beta-blocking agents and antiepileptics (OR = 0.39, 95% CI: 0.23–0.67)
- Infectious diseases (OR = 0.38, 95% CI: 0.20–0.70)

For peer review only

The RECORD statement – checklist of items, extended from the STROBE statement, that should be reported in observational studies using routinely collected health data.

	Item No.	STROBE items	Location in manuscript where items are reported	RECORD items	Location in manuscript where items are reported
**Title and abstract**					
	1	(a) Indicate the study's design with a commonly used term in the title or the abstract (b) Provide in the abstract an informative and balanced summary of what was done and what was found		RECORD 1.1: The type of data used should be specified in the title or abstract. When possible, the name of the databases used should be included.<br><br>RECORD 1.2: If applicable, the geographic region and timeframe within which the study took place should be reported in the title or abstract.<br><br>RECORD 1.3: If linkage between databases was conducted for the study, this should be clearly stated in the title or abstract.	Title<br>Abstract (line 34)<br><br>Line 6<br><br>Lines 35<br><br>Not applicable, only one hospital register
**Introduction**					
Background rationale	2	Explain the scientific background and rationale for the investigation being reported			Lines 75-148
Objectives	3	State specific objectives, including any prespecified hypotheses			Lines 145-148
**Methods**					
Study Design	4	Present key elements of study design early in the paper			Lines 151-153
Setting	5	Describe the setting, locations, and relevant dates, including periods of recruitment, exposure, follow-up, and data collection			Lines 155-177

For peer review only

Participants	6	*(a) Cohort study* - Give the eligibility criteria, and the sources and methods of selection of participants. Describe methods of follow-up<br>*Case-control study* - Give the eligibility criteria, and the sources and methods of case ascertainment and control selection. Give the rationale for the choice of cases and controls<br>*Cross-sectional study* - Give the eligibility criteria, and the sources and methods of selection of participants<br><br>*(b) Cohort study* - For matched studies, give matching criteria and number of exposed and unexposed<br>*Case-control study* - For matched studies, give matching criteria and the number of controls per case		RECORD 6.1: The methods of study population selection (such as codes or algorithms used to identify subjects) should be listed in detail. If this is not possible, an explanation should be provided.<br><br>RECORD 6.2: Any validation studies of the codes or algorithms used to select the population should be referenced. If validation was conducted for this study and not published elsewhere, detailed methods and results should be provided.<br><br>RECORD 6.3: If the study involved linkage of databases, consider use of a flow diagram or other graphical display to demonstrate the data linkage process, including the number of individuals with linked data at each stage.	Reported in a previous study: https://pubmed.ncbi.nlm.nih.gov/33973865/<br><br>Reported in a previous study: https://pubmed.ncbi.nlm.nih.gov/33973865/<br><br>Not applicable, only one hospital register
Variables	7	Clearly define all outcomes, exposures, predictors, potential confounders, and effect modifiers. Give diagnostic criteria, if applicable.		RECORD 7.1: A complete list of codes and algorithms used to classify exposures, outcomes, confounders, and effect modifiers should be provided. If these cannot be reported, an explanation should be provided.	Lines 181-222 and reported in a previous study: https://pubmed.ncbi.nlm.nih.gov/33973865/
Data sources/ measurement	8	For each variable of interest, give sources of data and details of methods of assessment (measurement).			Lines 181-222 and reported in a previous study:

For peer review only

		Describe comparability of assessment methods if there is more than one group			https://pubmed.ncbi.nlm.nih.gov/33973865/
Bias	9	Describe any efforts to address potential sources of bias			Lines 224-241 and reported in a previous study: https://pubmed.ncbi.nlm.nih.gov/33973865/
Study size	10	Explain how the study size was arrived at			Lines 155-177
Quantitative variables	11	Explain how quantitative variables were handled in the analyses. If applicable, describe which groupings were chosen, and why			Lines 224-241 and reported in a previous study: https://pubmed.ncbi.nlm.nih.gov/33973865/
Statistical methods	12	(a) Describe all statistical methods, including those used to control for confounding<br>(b) Describe any methods used to examine subgroups and interactions<br>(c) Explain how missing data were addressed<br>(d) *Cohort study* - If applicable, explain how loss to follow-up was addressed<br>*Case-control study* - If applicable, explain how matching of cases and controls was addressed<br>*Cross-sectional study* - If applicable, describe analytical			Lines 224-241

For peer review only

		methods taking account of sampling strategy<br>(e) Describe any sensitivity analyses			
Data access and cleaning methods		..		RECORD 12.1: Authors should describe the extent to which the investigators had access to the database population used to create the study population.<br><br>RECORD 12.2: Authors should provide information on the data cleaning methods used in the study.	Lines 224-241 and reported in a previous study: https://pubmed.ncbi.nlm.nih.gov/33973865/
Linkage		..		RECORD 12.3: State whether the study included person-level, institutional-level, or other data linkage across two or more databases. The methods of linkage and methods of linkage quality evaluation should be provided.	Reported in a previous study: https://pubmed.ncbi.nlm.nih.gov/33973865/
**Results**					
Participants	13	(a) Report the numbers of individuals at each stage of the study (*e.g.*, numbers potentially eligible, examined for eligibility, confirmed eligible, included in the study, completing follow-up, and analysed)<br>(b) Give reasons for non-participation at each stage.<br>(c) Consider use of a flow diagram		RECORD 13.1: Describe in detail the selection of the persons included in the study (*i.e.,* study population selection) including filtering based on data quality, data availability and linkage. The selection of included persons can be described in the text and/or by means of the study flow diagram.	Lines 170-177
Descriptive data	14	(a) Give characteristics of study participants (*e.g.*, demographic, clinical, social) and information on exposures and potential confounders			Lines 243-264

For peer review only - http://bmjopen.bmj.com/site/about/guidelines.xhtml

		(b) Indicate the number of participants with missing data for each variable of interest<br>(c) *Cohort study* - summarise follow-up time (*e.g.*, average and total amount)			
Outcome data	15	*Cohort study* - Report numbers of outcome events or summary measures over time<br>*Case-control study* - Report numbers in each exposure category, or summary measures of exposure<br>*Cross-sectional study* - Report numbers of outcome events or summary measures			Lines 243-264
Main results	16	(a) Give unadjusted estimates and, if applicable, confounder-adjusted estimates and their precision (e.g., 95% confidence interval). Make clear which confounders were adjusted for and why they were included<br>(b) Report category boundaries when continuous variables were categorized<br>(c) If relevant, consider translating estimates of relative risk into absolute risk for a meaningful time period			Lines 277-327
Other analyses	17	Report other analyses done—e.g., analyses of subgroups and interactions, and sensitivity analyses			Lines 268-280
**Discussion**					
Key results	18	Summarise key results with reference to study objectives			Lines 339-342

For peer review only

Limitations	19	Discuss limitations of the study, taking into account sources of potential bias or imprecision. Discuss both direction and magnitude of any potential bias		RECORD 19.1: Discuss the implications of using data that were not created or collected to answer the specific research question(s). Include discussion of misclassification bias, unmeasured confounding, missing data, and changing eligibility over time, as they pertain to the study being reported.	Lines 405-421
Interpretation	20	Give a cautious overall interpretation of results considering objectives, limitations, multiplicity of analyses, results from similar studies, and other relevant evidence			Lines 333-394
Generalisability	21	Discuss the generalisability (external validity) of the study results			Lines 396-400
**Other Information**					
Funding	22	Give the source of funding and the role of the funders for the present study and, if applicable, for the original study on which the present article is based			Line 451
Accessibility of protocol, raw data, and programming code		..		RECORD 22.1: Authors should provide information on how to access any supplemental information such as the study protocol, raw data, or programming code.	Lines 185, 188, 200-201<br><br>https://pubmed.ncbi.nlm.nih.gov/33973865/

*Reference: Benchimol EI, Smeeth L, Guttmann A, Harron K, Moher D, Petersen I, Sørensen HT, von Elm E, Langan SM, the RECORD Working Committee. The REporting of studies Conducted using Observational Routinely-collected health Data (RECORD) Statement. *PLoS Medicine* 2015; in press.

*Checklist is protected under Creative Commons Attribution (CC BY) license.

For peer review only - http://bmjopen.bmj.com/site/about/guidelines.xhtml
